# Integrating serial block-face SEM with voxel-based finite element analysis for high-fidelity micromechanical modelling of anisotropic soft tissues: application to human dermis

**DOI:** 10.1007/s10237-026-02090-6

**Published:** 2026-06-26

**Authors:** Jia Li, Orestis L. Katsamenis, Georges Limbert

**Affiliations:** 1 National Centre for Advanced Tribology at Southampton, Department of Mechanical Engineering, Faculty of Engineering and Physical Sciences, University of Southampton, University Road, Southampton, SO17 1BJ UK; 2 μ-VIS X-Ray Imaging Centre, Faculty of Engineering and Physical Sciences, University of Southampton, University Road, Southampton, SO17 1BJ UK; 3 Bioengineering Science Research Group, Department of Mechanical Engineering, Faculty of Engineering and Physical Sciences, University of Southampton, University Road, Southampton, SO17 1BJ UK; 4 Laboratory of Biomechanics and Mechanobiology, Division of Biomedical Engineering, Department of Human Biology, Faculty of Health Sciences, University of Cape Town, Cape Town, 7935 Observatory South Africa; 5 School of Mechanics and Aerospace Engineering, Dalian University of Technology, 2 Linggong Road, Dalian, 116024 People’s Republic of China

**Keywords:** Skin, Collagen, Fibre, Micromechanics, Voxel-based, Finite element, SBF-SEM

## Abstract

While continuum fibre-reinforced constitutive models of collagen-rich soft tissues incorporate microstructural information via structure tensor invariants, most of their numerical implementations assume spatially uniform fibre orientations. This study examined how spatially heterogeneous orientations, captured by high-resolution imaging and embedded in image-based finite element models, could provide novel mechanistic insights into tissue micromechanics. Serial block-face scanning electron microscopy (SBF-SEM) captured collagen architecture from fresh human skin dermis. Voxel-level 3D fibre orientations were extracted via structure tensor analysis. Voxel-based hexahedral meshes with element-level orientations were implemented in Abaqus/Standard with custom UMATs® user subroutines for invariant-based transversely isotropic hyperelasticity, and compared to classical models with spatially uniform fibre distributions under various loading conditions. Spatial heterogeneity significantly altered micromechanical responses. Under pseudo-homogeneous uniaxial extension aligned with the mean fibre orientation, Models 1A (uniform orientation, no dispersion), 1B (uniform orientation with dispersion), and 1C (spatially heterogeneous orientations) yielded nominal stresses at 44.6% Green–Lagrange strain differing by a factor of four (0.5, 0.8 and 2.1 MPa, respectively). Model 1C's maximum principal logarithmic strain showed a broader range with a secondary peak at 0.5 versus unimodal peaks at 0.3 for Models 1A/B. With a purely quadratic fibre energy, Model 1C recovered exponential-like macroscopic stiffening for fibre moduli of 25–500 MPa, confirming the J-shape arises from microstructural mechanism rather than through fibre material nonlinearity. The methodology delivers quantitative mechanistic insights into dermal micromechanics and generalises to a wide range of soft tissues from cornea and cartilage to arteries and lungs.

**Supplementary Information:**

The online version contains supplementary material available at https://doi.org/10.1007/s10237-026-02090-6.

## Introduction

As the most abundant protein in nature, collagen plays a fundamental structural and functional role in many biological classes from *Actinopterygii* (e.g. ray-finned fish) through *Mammalia* (i.e. mammalian species) to *Sipuncula* (e.g. peanut worms). Of particular significance to humans, collagen is a key element in maintaining skin mechanical integrity over the life course (He et al. 2023; Limbert et al. 2019; Naylor et al. 2011; Pissarenko and Meyers 2019). The chemo-mechanobiological evolution of dermal collagen and that of its hierarchical structure in response to intrinsic and extrinsic ageing is central to many aspects of skin health, wound healing, mental well-being, and, more generally, to how people interact with their physical and social environments (Limbert et al. 2019). Understanding the links between ageing-induced alterations of collagen multiscale structure and the mechanical response of skin is therefore a topic of significant interest, particularly in the light of the ageing of global biophysically-diverse populations.

At the structural level, the various types of collagen found in skin (e.g. Types I, III, VII) exhibit complex hierarchical architectures with distinct structural forms, including tropocollagen molecules as the basic building block (1.5 nm in diameter) (Buehler 2006b; Gautieri et al. 2011; Orgel et al. 2006), microfibrils (3.5–4 nm in diameter) (Gautieri et al. 2011; Orgel et al. 2006), fibril (12–500 nm, tissue-dependent) (Kadler 2017; Ricard-Blum 2011; Sherman, et al. 2015), and fibre (1–20 µm) (Sherman, et al. 2015). It is relevant to point out that in the dermatology literature fibrils are often termed “fibres” (Barton and Marks 1984). Fibres in turn associate into fibre bundles of 2–15 µm in cross section in the reticular dermis, organised in the well-documented basket-weave pattern (Brown 1972; Clemons et al. 2018; Meyer, et al. 2007; Pissarenko, et al. 2019; Starborg et al. 2013; Ueda et al. 2019; van Zuijlen et al. 2003). This structural arrangement provides resistance to shear and tensile forces while also enabling large structural deformations (Pissarenko and Meyers 2019). In general, as elemental building blocks, biological fibres, such as collagen fibres, endow their (macroscopic) assemblies with strongly directional properties which have been shown to be non-local (Krasny, et al. 2017). The mechanical response of these multiscale structures is thus conditioned by the local orientation of collagen fibres which give rise to inhomogeneous deformation modes.

Physics-based modelling of skin and its underlying collagen networks has been the subject of many investigative studies as this approach offers the ability to design hypothesis-driven research studies and identify or test mechanistic causal chains responsible for particular observed constitutive characteristics of skin mechanics (Benítez and Montáns 2017; Limbert 2017). From a constitutive modelling perspective, incorporating information about spatially-dependent fibre orientation (Alberini, et al. 2024a, 2024b) is essential for faithfully simulating the micromechanical behaviour of collagen-rich tissues.

There are multiple imaging techniques that offer the ability to image collagenous tissues with sufficient resolution to observe individual collagen fibres and bundles such as 2D histology, microcomputed X-ray tomography (micro-CT), and serial block-face scanning electron microscopy (SBF-SEM) (Denk and Horstmann 2004). 2D histology technique has been applied to observe the dermal architectures of biological tissues such as human skin (Hong et al. 2020) and porcine skin (Jor et al. 2011) at microscopic level. This method was then extended to three-dimensional (3D) histology with a combination of micro-CT imaging technique (Katsamenis, et al. 2019; Wang et al. 2015), enabling reconstruction of the tissue’s microstructures. As a non-destructive imaging technique, micro-CT offers submicron resolutions (Keklikoglou et al. 2021). Due to the low X-ray attenuation characteristic of soft tissues, contrast agents are commonly required to enhance the optical separation of internal structures. In the study by Dwivedi et al. (Dwivedi et al. 2022), micro-CT imaging was applied to examine the native arrangement and the orientation of collagen fibre bundles in pig skin. In addition, SBF-SEM and atomic force microscopy (AFM) were employed to study collagen fibre organisation and to characterise collagen fibrils at higher resolutions, respectively (Dwivedi et al. 2022). SBF-SEM imaging offers much higher in-plane and through-the-thickness resolution than micro-CT but this apparent advantage is mitigated by the anisotropic nature of voxel size which is limited by the slice thickness (25 to 50 nm in the best case scenario while the in-plane resolution can go down to 7 nm). Imaging of human skin dermis using SBF-SEM is reported in the work of Limbert (Limbert 2019a).

Moreover, unlike micro-CT, SBF-SEM is a destructive technique as the sample block is serially sectioned with a diamond knife. Combining micro-CT and SBF-SEM as part of the same imaging workflow is a powerful technique to conduct correlative imaging investigations (Laundon, et al., 2023). Apart from these high-resolution imaging techniques, other methods can provide microscopic level resolutions without the need for staining but the focus is typically on quantifying fibre orientation distribution through 2D images. For instance, second-harmonic generation (SHG) was employed by Freund et al. (Dudenkova et al. 2019; Freund et al. 1986) to visualise collagen in rat tail tendon with a spatial resolution of about 50 μm. Subsequently, SHG was used to analyse collagen fibre angles in soft tissues, revealing the influence of fibre-like structures on tissue function (Liu et al. 2017). This technique is particularly powerful for in vivo visualisation of collagen fibre arrangements particularly for age- or disease-related changes in human skin (Ogura et al. 2019) or in situ visualisation of collagen fibre reorientation under macroscopic loads (Allain et al. 2019; Jayyosi et al. 2017). SHG imaging penetration depth is limited, typically ranging from 100 to 200 μm in ex vivo samples and only a few tens of micrometres in vivo (Limbert 2019b).

In the last two decades, considerable research efforts have been devoted to the development of microstructurally based anisotropic continuum constitutive models of biological soft tissues (Holzapfel et al. 2025; Humphrey 2003). These formulations typically rely on the definition of one or more vector fields representing the local orientation of a family of biological fibres, usually, collagen fibres. Each discrete vector is associated with a corresponding structure tensor which is used to defined an anisotropic strain invariant representing the squared uniaxial stretch along the local fibre direction in the continuum (Humphrey and Yin 1987; Spencer 1984; Weiss et al. 1996). Methods to incorporate such structural information from imaging modalities into anatomically realistic micromechanical finite element (FE) models of soft tissues such as skin are still lagging behind, particularly when it comes to models aiming to capture the complex local three-dimensional (3D) architecture of the collagen fibres network. With few exceptions (Alberini, et al. 2024b; He et al. 2024), current state-of-the-art models of soft tissues only account for local fibre orientation in an averaged (i.e. statistical) sense (Gasser, et al. 2006) or are restricted to a two-dimensional (2D) setting (Ní Annaidh et al. 2012a, 2012b). More often than not, FE models of soft tissues in general, and those of skin in particular, featuring invariant-based fibre-reinforced continuum constitutive formulations, assume spatially invariant statistical distributions of fibre orientation. These distributions could be uniform (no fibre dispersion) or non-uniform (mean direction with fibre dispersion) such as the ubiquitous von Mises–Fisher (Gasser et al. 2006) and Bingham (Alastrué, et al. 2010; Gasser et al. 2012) distributions.

As the complex 3D architecture of collagen fibres is key in conditioning the mechanics of biological tissues, it is essential to integrate such critical information into the next generation of mechanistic biophysical models to support basic discovery research and a wide range of industrial and clinical applications from medical devices and consumer goods to virtual surgery simulators (Holzapfel, et al. 2025; Limbert 2017). Therefore, there is a need to develop robust methods and methodologies to seamlessly integrate the microstructural characteristics of the collagen network in biological tissues from high-resolution 3D imaging modalities.

In this paper, we report the development of such an approach combining SBF-SEM and imaging of human dermis, fibre orientation image analysis, voxel-based mesh generation, and micromechanical FE analyses. SBF-SEM was employed to resolve individual collagen fibres and sub-bundle architecture at nanometre to micrometre scales, which lie below the effective spatial and contrast resolution of laboratory X-ray-based techniques for soft tissues. The destructive and anisotropic nature of SBF-SEM was accepted as a necessary trade-off to achieve ultrastructural resolution and volumetric continuity within the imaged regions, enabling detailed 3D characterisation of collagen organisation at the fibre and bundle level. Besides the technological aspects of this work, the main scientific objective of this research was to quantify the effects of accounting for the spatially-varying mean orientation of collagen fibres on the micromechanics of a representative volume element (RVE) of human dermis. The paper is organised as follows. The next section presents the skin sample preparation and imaging protocols. In Sect. 3, the fibre tractography extraction method based on structure tensor analysis and its validation is presented. Section 4 describes the process for generating voxel-based 8-noded hexahedral FE mesh with assignment of local fibre orientation at element centroid, node, and integration point levels. Considering the potential very large number of Fes, a downsampling method is proposed and subsequently tested for accuracy when compared to full-resolution voxel-based mesh.

Constitutive equations and FE implementation aspects are presented in Sect. 5. In Sect. 6, a series of image-based 3D FE models of human dermis are constructed and tested under several types of loading scenarios. The mechanical response of models featuring locally assigned fibre orientation and globally assigned fibre orientation (i.e. all elements of the RVE are assigned the same mean orientation) with/without dispersion is quantitatively compared. This is achieved through a numerical homogenisation procedure to compute the equivalent stress–strain curve of their RVE. Sections 7 and 8, respectively, present and discuss the results of these numerical analyses and the general integrative approach proposed in this paper. Finally, the paper ends with concluding remarks.

## Sample preparation and imaging protocols

**Fig. 1:**
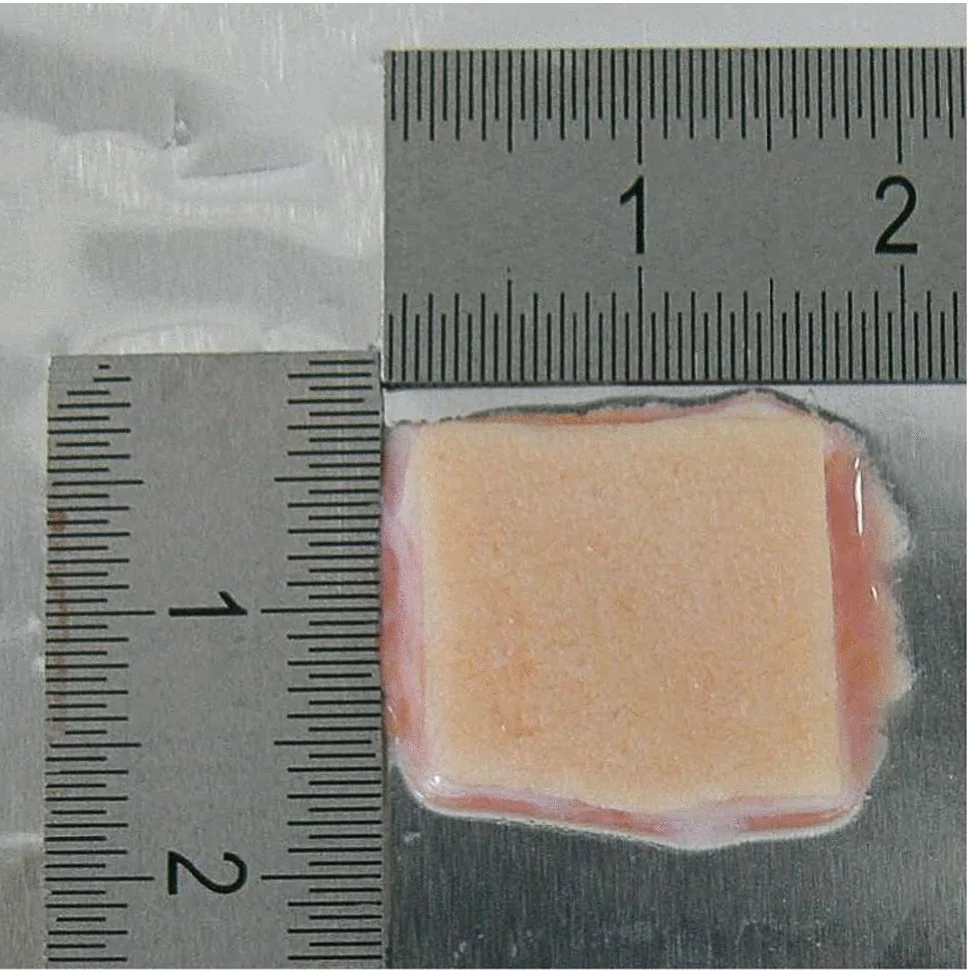
Full-thickness fresh abdominal skin sample (scale: centimetres)

**Fig. 2:**
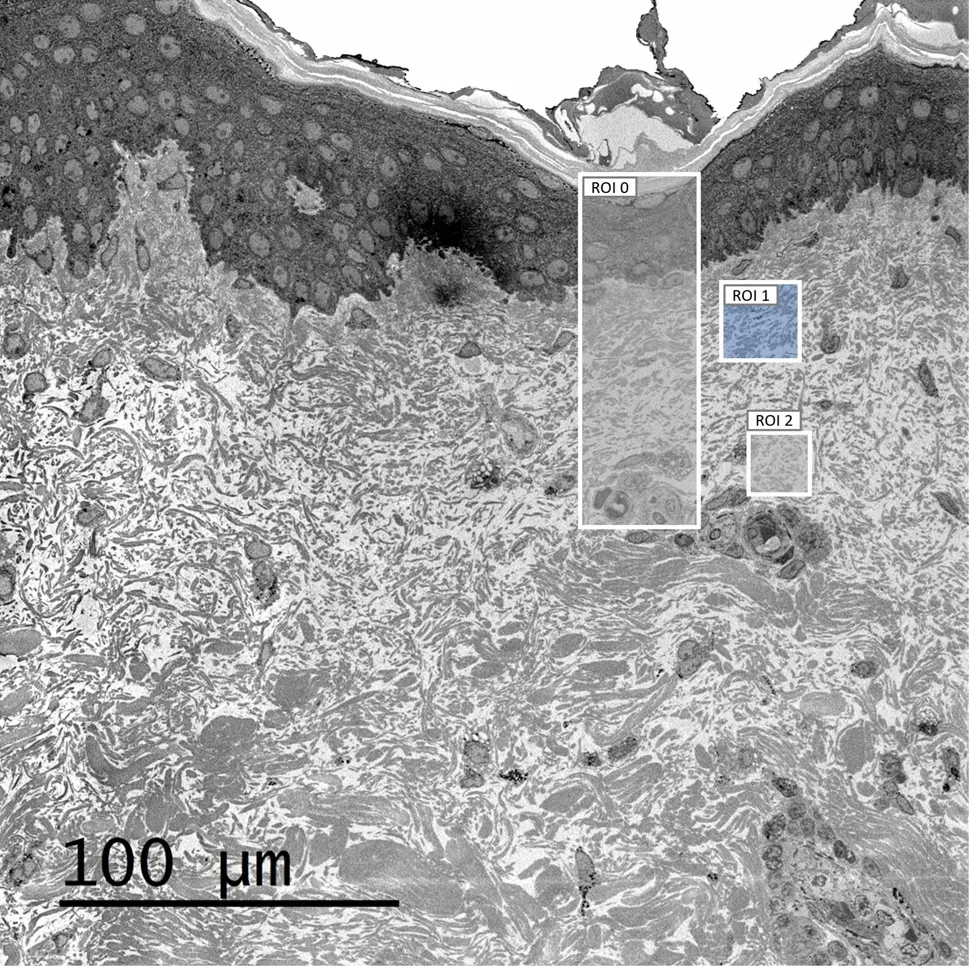
Regions of interest (ROI) identified at the surface of the prepared cubic skin sample (areas inside the white squares)

**Fig. 3:**
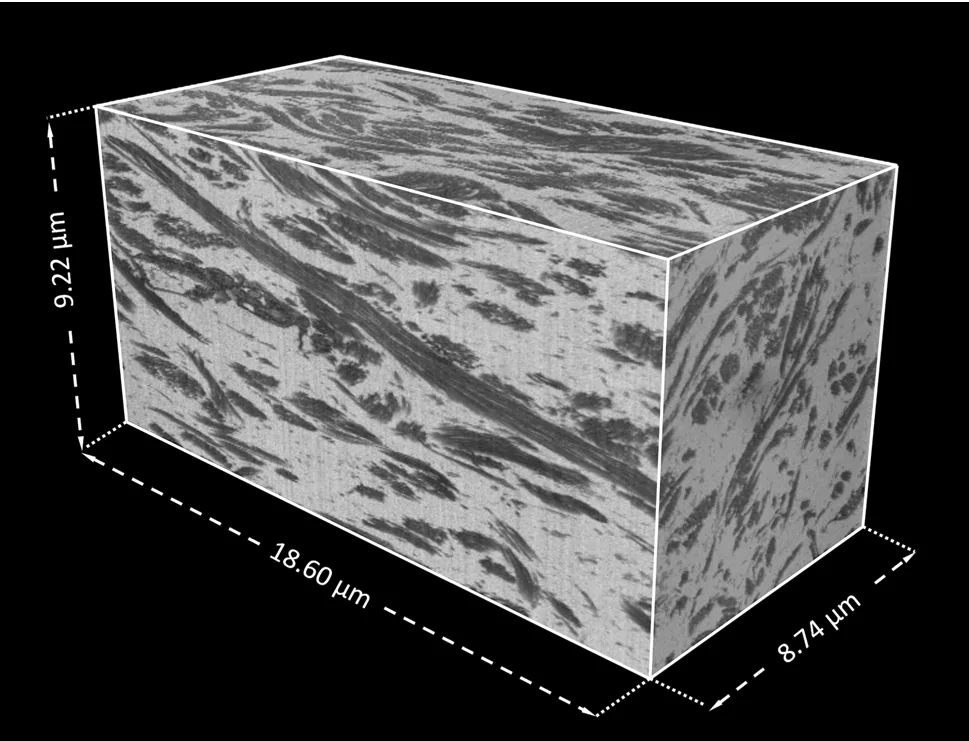
Visualisation of the 3D skin image stack resulting from the stacking of 373 images along the z-direction (50 nm spacing). The width, height, and length of the image stack are 8.74 µm, 9.22 µm, and 18.6 µm, respectively, while voxel dimensions along the *x*-, *y*-, and *z*-direction are 8 nm, 8 nm, and 50 nm, respectively

**Fig. 4:**
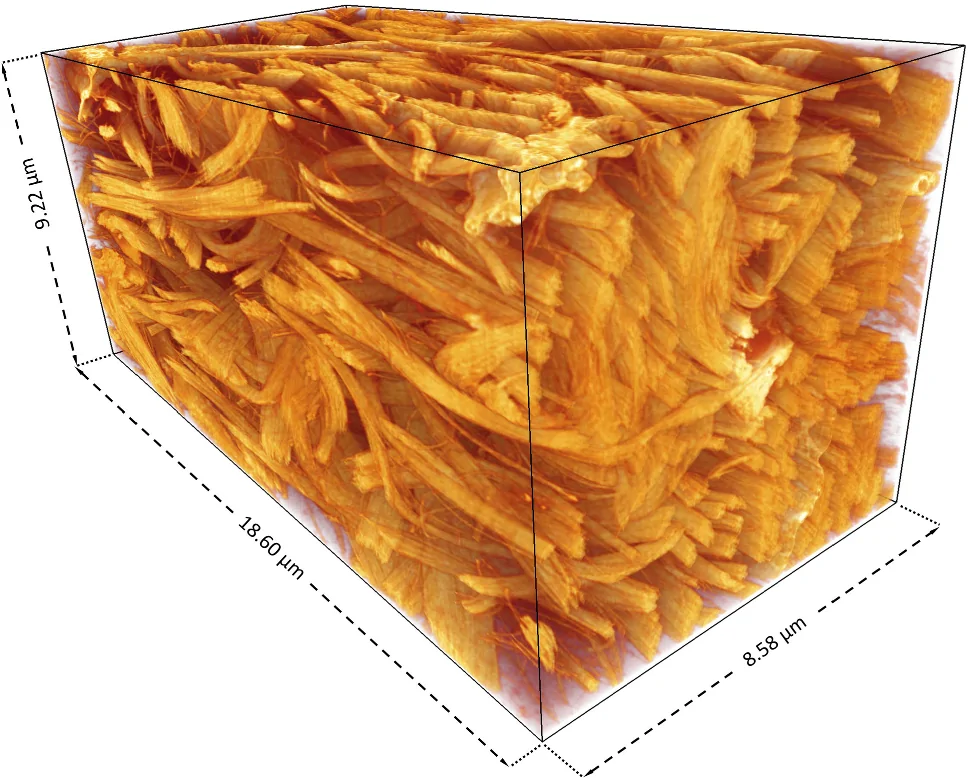
Volume rendering of the collagen fibre network within the reticular dermis layer of human skin (from the image stack depicted in Fig. 3 but using a different viewing angle and a slightly cropped volume). Three hierarchical levels are visible: (i) micron-scale fibre bundles, 1–3 µm in cross section, running across the volume at multiple non-parallel orientations consistent with a basket-weave organisation (Brown 1972; Pissarenko and Meyers 2019); (ii) within and at the edge of each bundle, individual fibres of ≈50–100 nm cross section appear as fine longitudinal striations, in agreement with the 56–63 nm fibre diameters reported for human dermis (Barton and Marks 1984); and (iii) inter-bundle voids occupied in vivo by proteoglycan-rich ground substance, here replaced by Spurr resin following ethanol dehydration. Microfibrils (~ 4 nm) and tropocollagen molecules (~ 1.5 nm) lie below the in-plane voxel size of 8 nm and are not resolved. Note that the apparent high collagen density is a consequence of the sample-preparation protocol: ethanol dehydration and resin embedding have replaced the aqueous ground substance (~ 70% by volume in vivo) with epoxy resin, so the visualised volume fraction of the collagenous phase is substantially higher than its native value

**Fig. 5:**
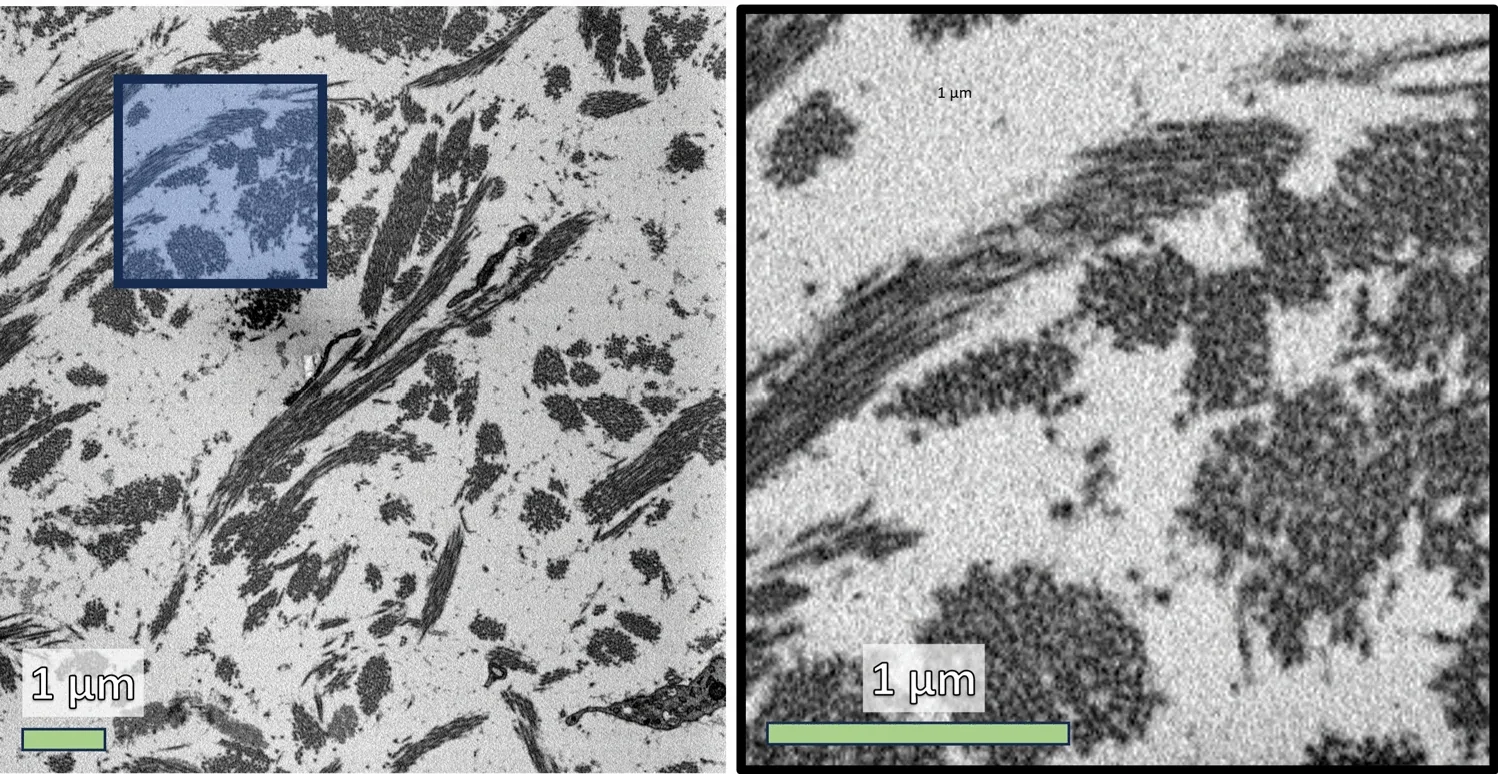
Single image part of the 373 pictures stack presented in Fig. 3. Left: full image (dimensions: 8.74 µm × 9.22 µm); right: zoomed-in view highlighting individual collagen fibres in- and out-of-plane

Ethically approved fresh abdominal skin extracted during a cosmetic surgery procedure (abdominoplasty) on a 39-year-old female Caucasian patient, with a body mass index of 25 and with no known medical conditions, was commercially obtained (TCS Cellworks, Buckingham, UK). Chlorhexidine was used for skin decontamination prior to surgery. The abdominal skin sample was harvested immediately post-excision and placed in ice-cold phosphate-buffered saline prior to. From this, a 1.5 × 1.5 cm full-thickness block (Fig. 1) was obtained by scalpel dissection perpendicular to the skin surface and shipped to our laboratory. The block was then cut into 1 mm-thick slices using a fresh double-edged razor blade and an aligned dermatome-style cutting guide (to preserve orthogonal orientation relative to the epidermis). For fixation, we followed the NCMIR (National Center for Microscopy and Imaging Research) rOTO (reduced Osmium, Thiocarbohydrazide, and Osmium tetroxide) protocol develop by Thomas Deerinck et al. (Deerinck et al., 2010) for high-contrast SBF-SEM sample preparation. The original protocol document is no longer available on the NCMIR’s web site but an updated version could be found (Deerinck et al., 2010; Denk and Horstmann 2004). This staining technique that improves tissue conductivity and membrane staining is standard for TEM/SBF-SEM collagen imaging (Courson, et al., 2021, Goggin et al. 2020, Laundon et al., 2023, Starborg, et al. 2013). After dehydration, the sample was embedded in Spurr resin (Agar Scientific, Stansted, UK). Trimming to a pyramidal block, gluing to the SBF-SEM aluminium pin with conductive silver epoxy, and gold/palladium sputter coating (~ 5 nm coat, Polaron SC7640, 2 × 30 s at 15 mA) were carried out following the Gatan 3View® manufacturer guidelines (Gatan, Inc., Pleasanton, CA, USA). These standard procedures for trimming, gluing, dehydration, resin embedding, and sputter coating can be found in the work of Laundon et al. (Laundon et al., 2023). To ensure the correct area was selected for the 3View, silver sections were cut on a Leica OMU 3 ultramicrotome (Leica Microsystems (UK) Ltd, Milton Keynes, UK), and viewed on a Hitachi H7000 transmission electron microscope (Hitachi High-Technologies Europe GmbH, Maidenhead, UK). The tissue block was then imaged in the serial block-face imaging system 3View® mounted inside a Zeiss Sigma VP field emission scanning electron microscope with variable pressure mode (Carl Zeiss Microscopy GmbH, Jena, Germany) and imaged at 2.5 kV. The acquisition was done as a multiregion of interest (ROI) with three areas at different defined sampling XY resolutions (Fig. 2). A total of 376 images with a 50 nm spacing along the z-direction (direction orthogonal to the imaging plane). ROI 0 was 7500 × 2500 pixels at 13 nm pixel size, ROI 1 was 2500 × 2500 pixels at 8 nm pixel size, and ROI 2 was 2500 × 2500 pixels at 6 nm pixel size. ROI 1 was the area selected for the first phase of the present study. The reconstructed volume corresponding to ROI 1 is depicted in Fig. 3. Figure 4 shows a volume rendering of the collagen fibre network within the reticular dermis of human skin, derived from this image stack (Fig. 3). Figure 5 presents a single image selected from the same image stack.

The SBF-SEM contrast readily distinguishes keratinised structures (hair follicles, ~ 70–100 µm) and lipid-filled sebocytes (sebaceous glands, ~ 100–500 µm) (Paus and Cotsarelis 1999; Schneider and Paus 2014) from the uniformly staining collagenous dermis. No such features were present in the subsequent ROIs selected for the analyses presented in this paper.

The generated skin image stack was pre-processed in Fiji/ImageJ using an in-house image denoise workflow that incorporates the XRH-Enhance routine from the XRH Processing Toolbox (Williams, et al. 2024), originally developed for X-ray histology applications (available at: https://zenodo.org/records/8253271). Images were first converted to 32-bit. An initial enhancement step was then applied using XRH-Enhance script with a (Gaussian radius = 1 pixel; 3D median filter diameter = 2 pixels; CLAHE (Contrast-Limited Adaptive Histogram Equalisation) disabled to reduce noise before further processing. To correct the pronounced brightness slope present in the serial block-face SEM stack, the enhanced volume was duplicated, blurred in 3D (10, 10, 10 pixels), gamma-corrected (0.5), and then used as a background estimate for division-based background correction of the original enhanced stack. The background-corrected volume was then duplicated and smoothed in 2D (Gaussian sigma = 1 pixel), and the smoothed image was subtracted from the original to perform unsharp masking and enhance local edge contrast. The processed images were then exported as 8-bit TIFF files.

The acquisition voxel size is anisotropic, 8 nm in-plane and 50 nm in the slicing direction. The in-plane sampling is well below fibre cross-sectional diameter, so individual fibres are resolved by 6–12 pixels in cross section and are not aliased; the 50 nm *z*-spacing is comparable to fibre diameter and constitutes the limit for separating two adjacent parallel fibres in the slicing direction, but it remains one to two orders of magnitude below the cross-sectional dimensions of the fibre bundles (1–15 µm) whose 3D geometry and orientation are the focus of the present modelling work. Fibre *morphology* (D-banding, microfibril packing, fibril-surface texture) is not part of this focus. Characterising those features would require subfibre (≲5 nm) resolution and is the domain of TEM tomography or cryo-electron tomography (Hulmes 2008; Orgel et al. 2006).

## Fibre orientation extraction and analysis

Early algorithms to extract fibre orientation in biological soft tissues focussed on quantifying in-plane averaged fibre orientation from 2D images, either by detecting spatial pixel intensity variations between fibrous structures and the surrounding matrix or by analysing orientation dependent patterns in the frequency domain. For example, the OrientationJ (Rezakhaniha, et al. 2012) plugin in Fiji (Schindelin et al. 2012) enables the characterisation of the local orientation of a region of interest (ROI) in an image based on 2D structure tensor analysis (Levillain et al. 2016; Püspöki et al. 2016; Rezakhaniha et al. 2012). Each pixel-based 2 × 2 structure tensor is derived from the image gradient and then decomposed to compute eigenvectors and eigenvalues. The eigenvector corresponding to the smallest eigenvalue indicates the direction of minimal intensity variation, which aligns with the local fibre orientation. 2D fast Fourier transform (FFT) (Schriefl et al. 2012) converts the image to the frequency domain and then identifies regular patterns formed by collagen fibres based on power spectrum. By applying a wedge-shaped orientation filter, the power spectrum within individual wedges such as 1° wedge width is summed to obtain corresponding amplitudes. For each ROI or the entire image, a discrete distribution of relative amplitudes as a function of angle is generated. The angles corresponding to peaks in the distribution are then shifted back by 90° to align with the preferred collagen fibre orientation in the real image. Compared to 2D pixel-wise Fourier analysis, the 2D weighted orientation vector summation algorithm (Quinn and Georgakoudi 2013) enables faster pixel-specific fibre orientation extraction with an accuracy of 2 to 3 degree. This method averages all vectors passing through the central pixel within a local window, weighting each vector by two factors related to pixel intensity fluctuations and the inverse of the vector length. Although fibre orientation information extracted by these 2D algorithms has been applied in computational modelling and potential disease diagnostics, their inherent limitation to two dimensions hinders their abilities to fully capture spatial variations in fibre structures. Notably, some studies have revealed the presence of out-of-plane fibres, highlighting the need for methods that can characterise 3D fibre architecture (Jor et al. 2011; Ní Annaidh et al. 2012a). Considering the intricate collagen network architectures observed in biological soft tissues and their influence on the mechanics of these tissues, it was only a matter of time before 2D algorithms would be extended to 3D. In that spirit, researchers (Lau, et al. 2012; Lee, et al. 2020) used 3D FFT for characterising fibre orientation metrics in porcine tendons. 3D FFT is a generalisation of 2D FFT but applies a filter bank consisting of 3D orientation filters constructed in the Fourier space to analyse the power spectrum. The orientation filter with the maximum correlation between filter banks and results of Fourier transform represents the primary fibre orientation for the tested volume of interest (VOI). However, this algorithm mainly targets the overall fibre orientation of each VOI; therefore, the information on fibre distribution within each volume is lost when multiple fibre families exist. To address this problem, Alberini et al. (Alberini, et al. 2024a, 2024b) recently proposed a novel 3D FFT-based algorithm that generates a discrete-fibre orientation distribution (dFOD) for all fibre families. By utilising a combination of bivariate von Mises probability density function to fit the deconvoluted dFOD, the number of fibre families and their corresponding mean fibre angle, in-plane, and out-of-plane fibre dispersion are computed. Since the analysis occurs in the frequency domain, this method is less impacted by overlapping fibres. Nevertheless, the selection of VOI size remains a trade-off between spatial resolution and computational efficiency (Lau, et al. 2012). To rapidly quantify voxel-based fibre orientation from images, a 3D weighted orientation vector summation algorithm was proposed (Liu, et al. 2017, 2015). It is based on the projection of 3D fibres within a local window onto three orthogonal planes whereby the angle of fibre projection relative to the horizontal axis within each plane is computed. Using a mathematical relationship between these projection angles, the fibre azimuth and inclination angles are generated for each voxel. Given that each image stack can be regarded as a 3D array storing pixel intensity values (and an associated slice thickness value), it is possible to calculate intensity gradients along the three spatial directions. 3D structure tensor analysis enables to characterise local fibre orientation at each point (i.e. voxel location) through an eigendecomposition of the local structure tensor (Karjalainen, et al. 2021). Such a computing method is generally time-consuming, particularly for large volumetric datasets.

Recent developments such as a Python-based implementation (Jeppesen, et al. 2020, 2021) have optimised the process by utilising the separability of 3D Gaussian kernel that can be expressed as a product of three 1D Gaussian kernels and parallel computations on multicore CPUs and GPUs, significantly improving the analysis speed for extracting voxel-based fibre orientation from CT images. In the study reported in this paper, we followed a 3D structure tensor approach to determine local orientations of collagen fibres at individual voxel level. Details of this approach are presented in the next section.

### Structure tensor image analysis—methodology

Structure tensor analysis (Jor, et al. 2011; Karjalainen, et al. 2021) has proved to be an effective mathematical technique in image processing and computer vision in to determine local orientation of fibre-like structures. This approach works equally well in 2D and 3D. Here, we follow the implementation of Jeppesen et al. (Jeppesen, et al. 2021). Without loss of generality, we present the general concept and equations of structure tensor analysis. An image can be viewed as a 2D array of pixel intensity values while a stack of 2D images featuring a specific spacing along the third dimension becomes a 3D array of pixel intensity values I(x,y,z), indexed by spatial position ${\boldsymbol{p}} = (x,y,z)$. Pixel intensity values can be defined per each colour channel (e.g. red, green, and blue for RGB images) or, as a single grey level value for SBF-SEM and micro-CT images. The matrix representation of a 3D structure tensor describes the local orientation properties in the neighbouring area (i.e. a group or window of voxels the size of which must be selected) of a specific point in a space ${\boldsymbol{p}}_{0} = (x_{0} ,y_{0} ,z_{0} )$. The *continuous* (as opposed to *discrete*) structure tensor is derived from the gradient of the image intensity:

1 $${\boldsymbol{G}} = \nabla I(x,y,z) = \left[ {\begin{array}{*{20}c} {\frac{\partial I}{{\partial x}}} & {\frac{\partial I}{{\partial y}}} & {\frac{\partial I}{{\partial z}}} \\ \end{array} } \right]^{{\mathrm{T}}}$$

which leads to the definition of the continuous structure tensor via the use of the tensor outer product operator denoted by the symbol ⊗:

2 $${\boldsymbol{S}} = {\boldsymbol{S}}(x,y,z) = {\boldsymbol{G}}(x,y,z) \otimes {\boldsymbol{G}}(x,y,z)$$

The continuous local structure tensor is thus explicitly defined as:

3 $${\boldsymbol{S}} = \left( {\frac{\partial I}{{\partial x}}} \right)^{2} {\boldsymbol{B}}_{xx} + \left( {\frac{\partial I}{{\partial y}}} \right)^{2} {\boldsymbol{B}}_{yy} + \left( {\frac{\partial I}{{\partial z}}} \right)^{2} {\boldsymbol{B}}_{zz} + \frac{\partial I}{{\partial x}}\frac{\partial I}{{\partial y}}\left( {{\boldsymbol{B}}_{xy} + {\boldsymbol{B}}_{yx} } \right) + \frac{\partial I}{{\partial x}}\frac{\partial I}{{\partial z}}\left( {{\boldsymbol{B}}_{xz} + {\boldsymbol{B}}_{zx} } \right) + \frac{\partial I}{{\partial y}}\frac{\partial I}{{\partial z}}\left( {{\boldsymbol{B}}_{yz} + {\boldsymbol{B}}_{zy} } \right)$$

where $\left\{ {{\boldsymbol{B}}_{ij} } \right\} = \left\{ {{\boldsymbol{e}}_{i} \otimes {\boldsymbol{e}}_{j} } \right\}\left( {i,j = x,y,z} \right)$ are tensor bases defined through the orthonormal unit vectors $\left\{ {{\boldsymbol{e}}_{i} } \right\}\left( {i = x,y,z} \right)$.

Calculation of the gradient of an image can be done using a finite difference scheme or through the use of a derivative filter. Noise filtering is typically applied prior to the computation of gradients. This can be achieved by convolving the image (I(x,y,z)) with a Gaussian filter with standard deviation σ, and by definition, zero mean. The associated Gaussian functions in 2D and 3D are, respectively:

4 $${\mathcal{G}}^{{{\mathrm{2D}}}} (x,y,\sigma ) = \frac{1}{{2\pi \sigma^{2} }}\exp \left( { - \frac{{x^{2} + y^{2} }}{{2\sigma^{2} }}} \right)$$

and

5 $${\mathcal{G}}^{{{\mathrm{3D}}}} (x,y,z,\sigma ) = \frac{1}{{2\sqrt 2 \pi^{2} \sigma^{3} }}\exp \left( { - \frac{{x^{2} + y^{2} + z^{2} }}{{2\sigma^{2} }}} \right)$$

It follows that:

6 $$I^{\sigma } (x,y,z,\sigma ) = {\mathcal{G}}^{{{\mathrm{3D}}}} (x,y,z,\sigma )*I(x,y,z) = \sum\nolimits_{i,j,k} {{\mathcal{G}}^{{{\mathrm{3D}}}} (i,j,k,\sigma )} I(x - i,y - j,z - k)$$

leading to the denoised image gradient ${\boldsymbol{G}}^{\sigma } (x,y,z,\sigma ) = \nabla I^{\sigma } (x,y,z,\sigma )$ and the corresponding structure tensor ${\boldsymbol{S}}^{\sigma }$:

7 $${\boldsymbol{S}}^{\sigma } = {\boldsymbol{S}}^{\sigma } (x,y,z,\sigma ) = {\boldsymbol{G}}^{\sigma } (x,y,z,\sigma ) \otimes {\boldsymbol{G}}^{\sigma } (x,y,z,\sigma )$$

Application of structure tensor analysis for the detection of oriented features in an image or a stack of images requires the consideration of a “window” which represents a local surface or volume neighbourhood (i.e. a group of pixels/voxels) around a given pixel/voxel. This can be viewed as a scale factor characterising the domain of integration in the convolution operation used to smooth out the structure tensor components. The structure tensor obtained after the denoising procedure, ${\boldsymbol{S}}^{\sigma }$, is then convolved with a Gaussian kernel of standard deviation ρ which leads to the final structure tensor ${\boldsymbol{S}}^{\sigma |\rho } = {\boldsymbol{S}}^{\sigma |\rho } (x,y,z,\sigma ,\rho )$:

8 $${\boldsymbol{S}}^{\sigma |\rho } = {\boldsymbol{S}}^{\sigma |\rho } (x,y,z,\sigma ,\rho ) = {\mathcal{G}}^{{{\mathrm{3D}}}} (x,y,z,\rho )*{\boldsymbol{S}}^{\sigma } (x,y,z,\sigma ) = \sum\nolimits_{i,j,k} {{\mathcal{G}}^{{{\mathrm{3D}}}} (i,j,k,\rho )\,} I(x - i,y - j,z - k,\sigma )$$

For a given pixel/voxel neighbourhood (or window), the two convolution operations effectively blur the image and components of the structure tensor, thus reducing noise and integrating information about the microstructure and its internal length scale. Both Gaussian parameters ρ and σ condition the scale of features detected and robustness to the presence of noise in the images. In the context of fibrous materials, Auenhammer et al. (Auenhammer, et al. 2022) recommend to select these parameters as a function of the mean radius of fibres $r_{fibre}$, measured in voxel unit:

9 $$\sigma _{{fibre}} = \frac{{r_{{fibre}} }}{{\sqrt 2 }};\,\,\rho _{{fibre}} = 4\sigma _{{fibre}} = 2\sqrt 2 r_{{fibre}}$$

Once the structure tensors have been calculated at every voxel, their eigenvalues and eigenvectors are calculated. The eigenvector associated with the smallest eigenvalue corresponds to the orientation of least change in intensity which is therefore the orientation aligned with the local structural feature (i.e. fibre). It is worth noting that we speak about orientation and not direction which endows an orientation with a positive or negative sense. Structure tensors are *even* functions of orientation.

Considering that the fibre orientation field used as part of the finite element-level constitutive properties is not extracted voxel-by-voxel it is important to highlight the following point: the structure tensor is integrated over an outer Gaussian neighbourhood whose support is set deliberately above fibre diameter, so that the effective representative volume element for orientation extraction encompasses many fibres within a bundle, in line with the structure tensor framework used for soft tissue mechanics in other studies (Niestrawska, et al. 2016). The 50 nm figure is therefore the acquisition voxel size; the orientation-sampling RVE is one to two orders of magnitude larger and lies, by construction, well above fibre diameter as required for a representative directional measure.

The open-source Python code developed by Jeppesen and colleagues (Jeppesen, et al. 2020, 2021) was employed and extended to implement the 3D structure tensor analysis of our skin image data. This code was selected for its transparent and simple Python implementation that exploits efficient GPU computing architecture available on modern graphics cards.

**Fig. 6:**
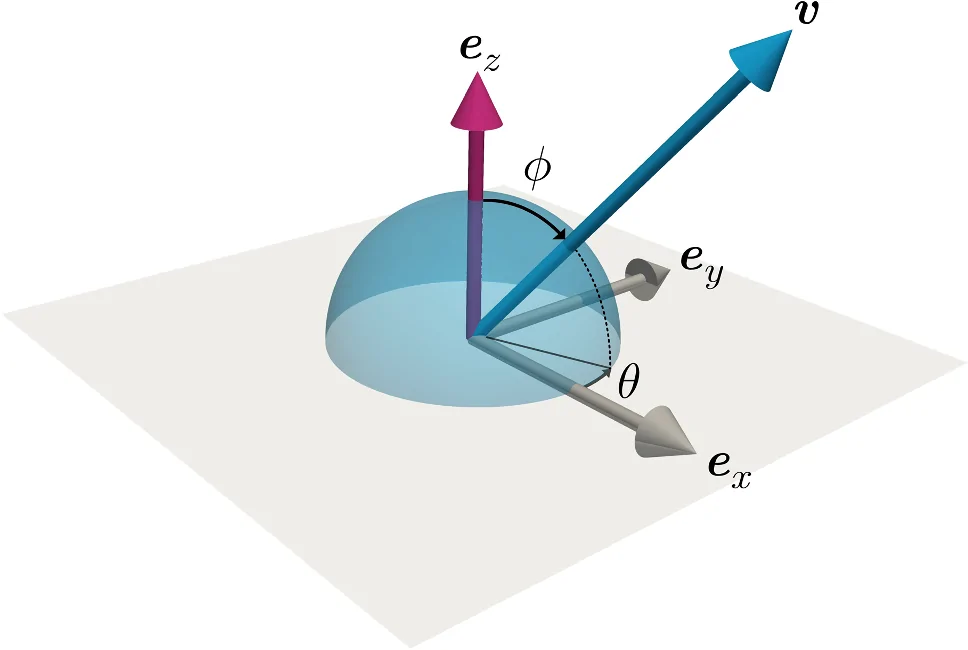
Schematic describing the conventions and representation of a (fibre orientation) vector ${\boldsymbol{v}}$ embedded into an hemisphere, defined by an azimuthal angle θ and an inclination angle ϕ referring to a rectangular Cartesian coordinate system defined by the unit basis vectors $\{ {\boldsymbol{e}}_{x} ,{\boldsymbol{e}}_{y} ,{\boldsymbol{e}}_{z} \}$

Depending on the implementation of the eigensolver, an eigenvector ${\boldsymbol{v}}$ may be computed as either ${\boldsymbol{v}}$ or as its reflection across the plane normal to its orientation, $- {\boldsymbol{v}}$ (Jeppesen, et al. 2021). In order to standardise our eigenvector calculations and the subsequent generation of voxel-based FE meshes, all eigenvectors were constrained to lay in the hemisphere defined by the positive direction along the ${\boldsymbol{e}}_{z}$ basis vector (Fig. 6). It means that the Cartesian *z* components of (fibre) eigenvectors are always positive, thus leading to the following azimuthal and inclination angle ranges: $\theta \in [ - \pi ,\pi ]$ and $\phi \in [0,\pi /2]$.

The 3D fibre vector extract method was validated for various synthetically generated volumes containing single or multiple fibres (see Sect. 13. Supplementary material 1—Validation of fibre extraction method).

## Generation of voxel-based finite element mesh with local fibre orientation

At this stage, it is worth reiterating that in our modelling approach collagen fibres are not modelled *explicitly* as *per* their real complex geometry extracted from images but, *implicitly*, through the definition of an anisotropic elastic energy contribution. This energy which must satisfy the principle of material frame indifference is an even function of the fibre stretch along the fibre longitudinal axis (Holzapfel 2000). Fibre geometric characteristics are therefore encoded through the definition of a vector field. Details of the microstructurally based continuum constitutive formulation are provided in Sect. 5.

The idea behind voxel-based mesh generation is to simply convert voxels from an image stack into hexahedral finite elements. In the last three decades, this approach has been widely used in bone mechanics research (Sas, et al. 2020; van Rietbergen, et al. 1995) but presents challenges such as the production of non-smooth boundaries without employing hybrid tetrahedral/hexahedral meshing, and, of particular relevance in the present study, the generation of very large element/node counts for high-resolution images. Therefore, it is essential to consider downsampling procedures to minimise the size of subsequent finite element models. This will be discussed later in this section.

**Fig. 7:**
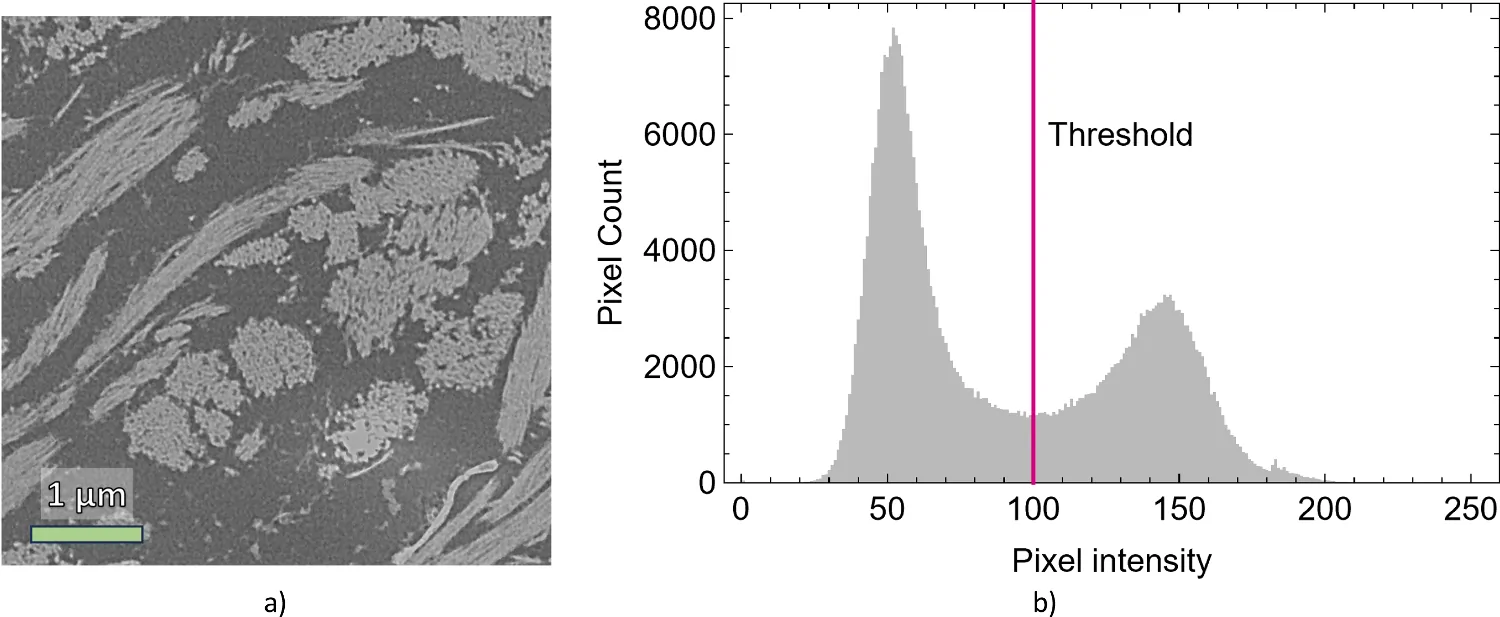
**a** SBF-SEM image of the dermis following basic pre-processing (denoising and level normalisation) and **b** its corresponding intensity histogram where the vertical red line defines the threshold value determined using the Otsu’s method (Otsu 1975)

The structure tensor extraction methodology described in the previous section outputs a single fibre vector for each voxel of the image stack. It is worth pointing out that in general SBF-SEM images may contain other structures besides collagen fibres and the isotropic matrix such as hair follicles or sebaceous glands. The ROIs selected for the analyses presented here only contained collagen fibre and matrix phases. Voxels encompassing the fibre phase have higher intensities than those of the matrix in the SBF-SEM volume. Therefore, Otsu’s method (Otsu 1975) was applied to automatically determine the image thresholding value required to separate fibre and matrix phases. This technique works by maximising the intra-class intensity variance and performs well when image intensities exhibit a bimodal distribution characterised by two peaks separated by a deep valley. Once the image threshold value is determined, voxels with higher intensity than the threshold are considered to be fibre elements while voxels featuring an intensity lower than the threshold are deemed to be matrix elements (Fig. 7).

**Fig. 8:**
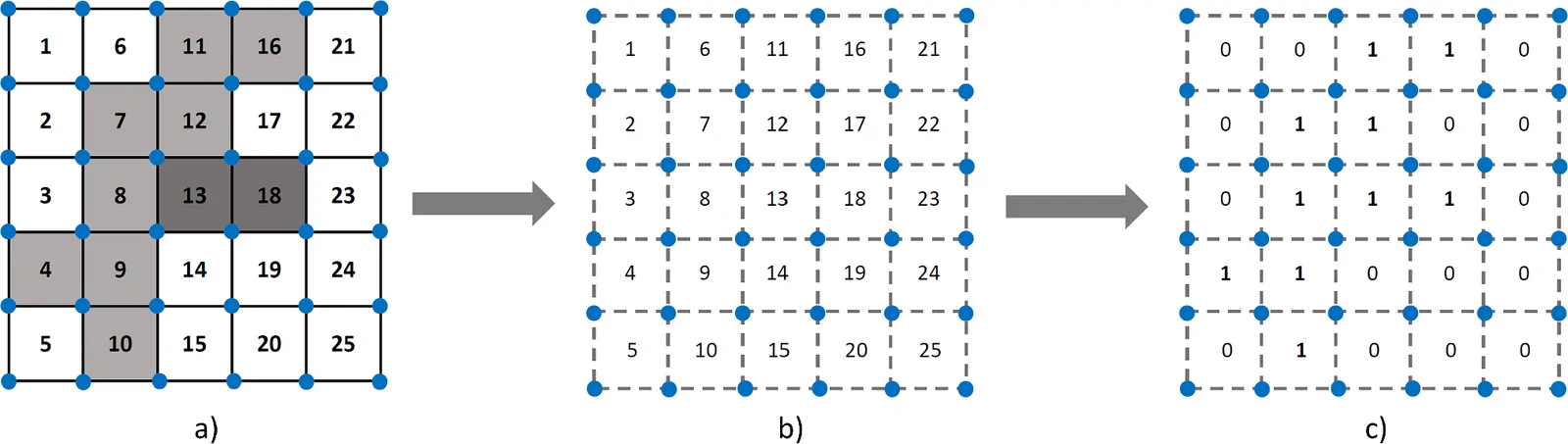
Schematic representation of the voxel-based mesh generation process with assignment of fibre and matrix phases to each finite element. For sake of clarity, the schematic is restricted to 2D: pixels can be viewed as voxel with zero third dimension. Each pixel/voxel is endowed with its unique fibre vector

In our Python programme, after execution of the structure tensor extraction script, an ASCII file is generated. This file contained each voxel id, its *index* coordinates in the image stack, its 8 vertex *physical* coordinates calculated from the index coordinates, its vertex connectivity table, the pixel dimensions and image thickness, the three physical coordinates of its normalised fibre vector, and an integer flag specifying whether the voxel belongs to the fibre phase (flag = 1) or the matrix phase (flag = 0) (Fig. 8).

In a finite element context, fibre vector information could be specified at element (Sect. 4.1), node (Sect. 0), or integration point level (Sect. 4.3). In the case of a Lobatto integration scheme, non-internal integration points are collocated with nodes.

### Fibre vector assignment at element centroid

**Fig. 9:**
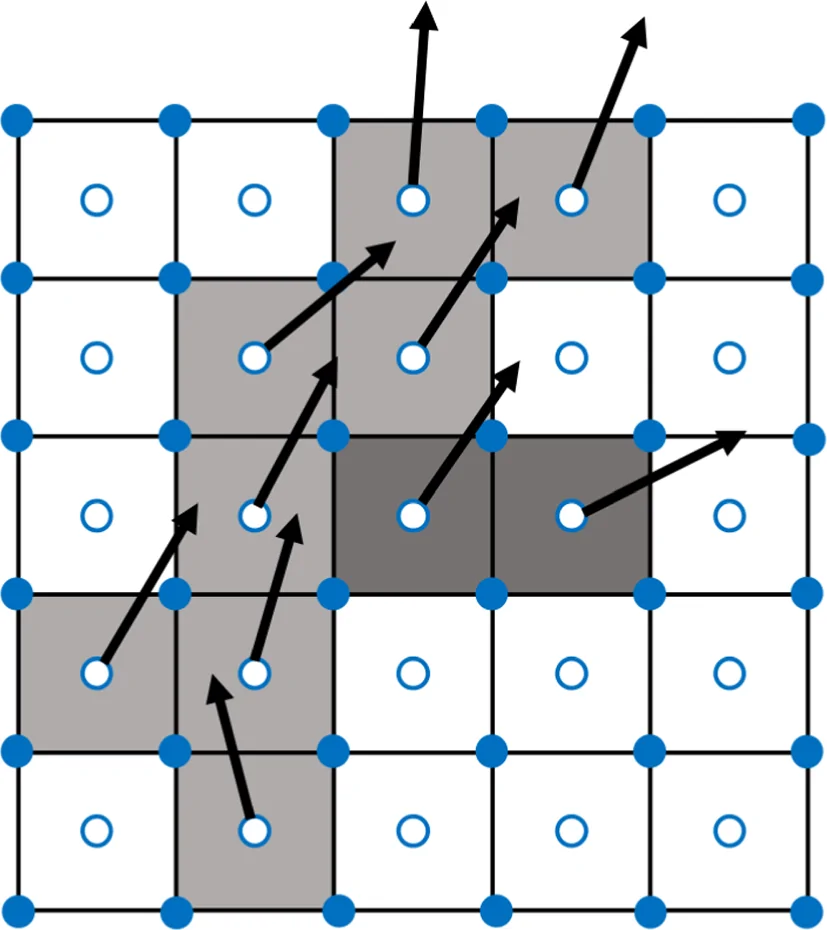
Schematic representation of the assignment of fibre vector process as part of the voxel-based mesh generation. For sake of clarity, the schematic is restricted to 2D: pixels can be viewed as voxel with zero third dimension. Each pixel/voxel is endowed with its unique fibre vector which is constant everywhere inside the element

As the raw output of the structure tensor extraction method consists of a unique fibre vector assigned to each voxel, it is straightforward to create a voxel-based mesh made of 8-noded hexahedrons which mirror their topologically equivalent voxels. The procedure is illustrated in Fig. 9 but, for sake of clarity and simplicity, presented for the 2D case only.

Our Python application enables the export of the mesh and its attributes (fibre vector and phase flag) as a VTK file and Abaqus® mesh file (Simulia, Dassault Systèmes, Providence, RI, USA). In both cases, mesh attributes are defined at element level (i.e. cell level). Export to the VTK format was implemented to enable advanced visualisation options using the open-source application ParaView (Kitware, Inc., New York, USA) (Ahrens, et al. 2001).

### Fibre vector assignment at nodes

**Fig. 10:**
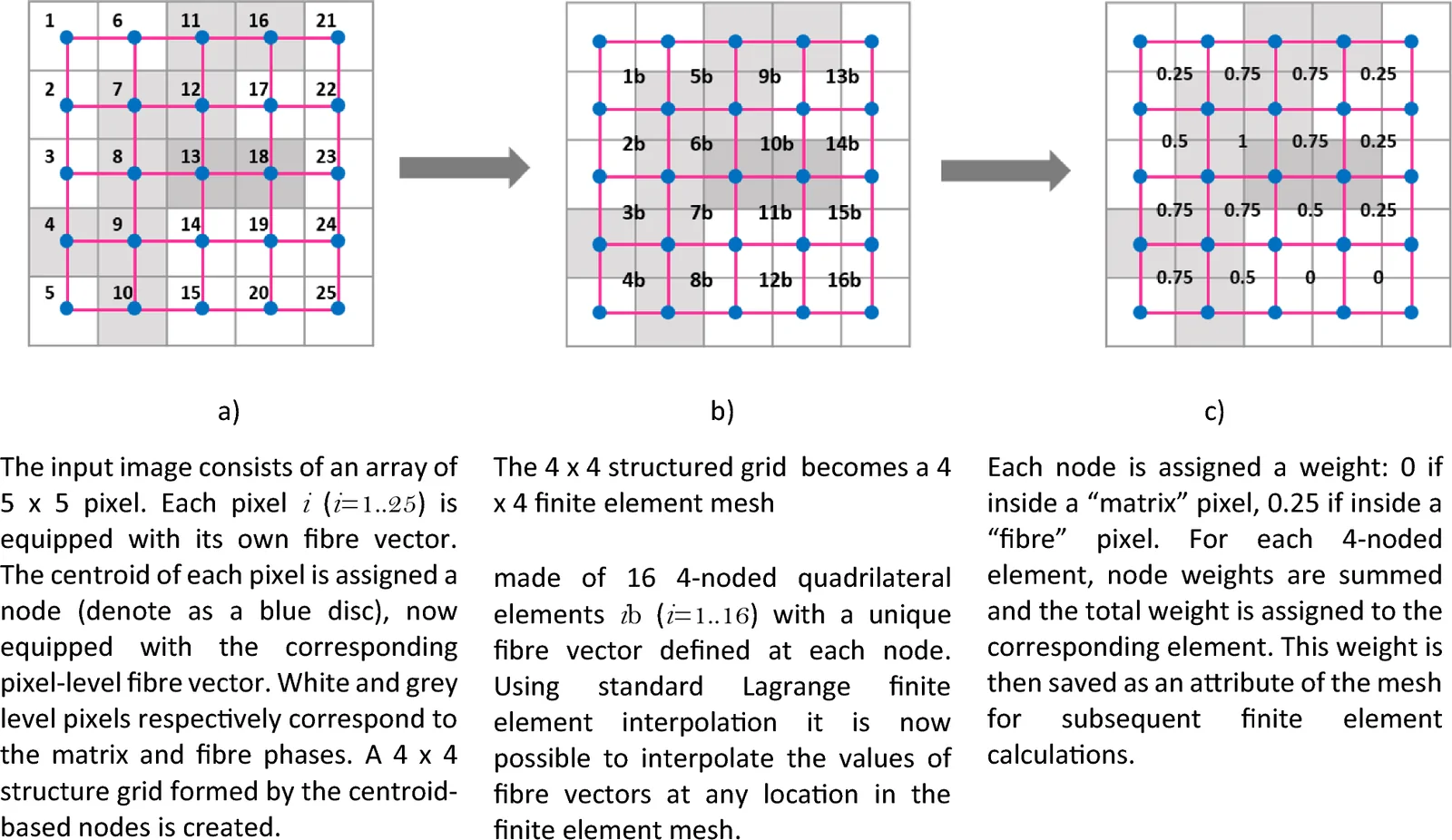
Schematic representation of the voxel-based mesh generation process with assignment of fibre and matrix phases to each finite element. For sake of clarity, the schematic is restricted to 2D: pixels can be viewed as voxel with zero length third dimension. Each pixel/voxel is endowed with its unique fibre vector

In a finite element context, the displacement field is continuous across elements. However, stress and strain fields are calculated at integration points. Therefore, it is sensible to define the fibre vector field at integration points in an element, and not simply as identical discrete values for all integration points like what is done in the method described in the previous section. In this section, a simple approach is proposed to first define fibre vectors at each node of a finite element mesh and then use these nodal vectors to calculate unique fibre vectors at each integration point. The idea is to construct a grid using the element centroids (as defined in Sect. 4.1) as nodes of the new finite element mesh. The principle of the procedure is illustrated in Fig. 10 but using a 2D analogy for sake of simplicity. Instead of considering 8-noded hexahedral elements, 4-noded quadrilateral elements are used. The method reduces the size of the image stack by a single pixel in each directions which is clearly negligible for our purpose.

In order to offer flexibility in how our algorithm determines whether a voxel is a “fibre” or “matrix” voxel, it was decided to calculate a *fibre pixel ratio*. This metrics was defined as follows: each finite element node was assigned a weight: 0 if inside a “matrix” pixel, 0.25 if inside a “fibre” pixel (intensity thresholding determined using the Otsu’s method (Otsu 1975)). For each 4-noded element, node weights were summed and the total weight was assigned to the corresponding element. This weight, or fibre pixel ratio, is then saved as an attribute of the mesh for subsequent finite element calculations. By specifying and varying a fibre pixel ratio threshold above which a pixel (and its associated voxel) is considered to be “fibre” pixel, it is possible to explore the effects of this parameter on the resulting micromechanics of the dermis.

The nodal values of each fibre vector $\boldsymbol{v}^{{(i)}} = [v_{x}^{{(i)}} ,v_{y}^{{(i)}} ,v_{z}^{{(i)}} ]^{{\mathrm{T}}}$ (defined at node i), generated through the structure tensor analysis presented in Sect. 3, can be used to interpolate nodal values anywhere in the corresponding finite element. This is the approach that was used to determine fibre vectors at integration point level (see next section). A simple linear Lagrange interpolation scheme (Liu and Quek 2013) is used such that the interpolated fibre vector $\boldsymbol{v} = [v_{x} ,v_{y} ,v_{z} ]^{{\mathrm{T}}}$ is obtained as:

10 $${\boldsymbol{v}} = N_{i} {\boldsymbol{v}}^{(i)} = N_{i} [v_{x}^{(i)} ,v_{y}^{(i)} ,v_{z}^{(i)} ]^{{\mathrm{T}}}$$

using the standard summation convention if an index is repeated (here, for a linear hexahedron i varies from 1 to 8). The shape functions are provided in Sect. 14. Supplementary material 2—Finite element Lagrange shape functions). In 3D, 2 images are necessary to define an array of voxels/3D finite elements. The image thickness (distance between two images of the 3D stack) defines the distance between two opposing faces of an hexahedron. Our Python application enables the export of a voxel-based finite element mesh with node-based fibre vectors to the VTK format for visualisation purpose in ParaView.

### Fibre vector assignment at element integration points

Using the parametric space defined by the unit vectors $(\xi {\boldsymbol{e}}_{x} ,\eta {\boldsymbol{e}}_{y} ,\zeta {\boldsymbol{e}}_{z} )$ and the nodal values of the fibre vector, the interpolated values of the fibre vector at the j-th integration point of the finite element under consideration are:

11 $${\boldsymbol{v}}(\xi_{j} ,\eta_{j} ,\zeta_{j} ) = N_{i} (\xi_{j} ,\eta_{j} ,\zeta_{j} ){\boldsymbol{v}}^{(i)} = N_{i} [v_{x}^{(i)} ,v_{y}^{(i)} ,v_{z}^{(i)} ]^{{\mathrm{T}}} (\xi_{j} {\boldsymbol{e}}_{x} ,\eta_{j} {\boldsymbol{e}}_{y} ,\zeta_{j} {\boldsymbol{e}}_{z} )$$

where $(\xi ,\eta ,\zeta )$ are the parametric coordinates of the integration point of index j. Linear quadrilaterals and hexahedrons are, respectively, equipped with 4 and 8 integration points.

Our Python application can then export the voxel-based mesh with integration point level fibre vector assignment to the VTK and Abaqus® file formats. For the Abaqus® model input file, information about fibre vector information at integration points is written to an ASCII file which can be subsequently read by Abaqus® Fortran user subroutines, namely UEXTERNALDB which has the advantages of featuring file operations compatible with parallel finite element analyses on multiple compute nodes, not simply multiple processors and cores on the same node. This subroutine is called only once, at the beginning of an analysis, from within the user material subroutine UMAT to initialise the state variables that store the components of fibre vectors at each integration point. The subroutine UMAT —to be used with the Abaqus/Standard® implicit solver—allows the coding of arbitrary constitutive behaviour (more details in Sect. 5).

As alluded at the beginning of this section, the combination of high-resolution imaging and voxel-based finite element mesh generation can lead to excessively large models which do not only require large amount of RAM and computing power but also high-capacity permanent storage and high computing requirements for real-time visualisation. These aspects must be considered and this is why, besides the availability of standard image downsampling, we implemented a fibre vector field downsampling algorithm which is described in Sect. 4.4.

One should point out that typical SBF-SEM acquisition leads to anisotropic voxel dimensions. For example, with a resolution of 8 nm per pixel per image, the third dimension of a voxel which is normal to the image plane is restricted to the physical spacing between sample slices, itself determined by the physical capability of the microtome used (i.e. 50 nm-thick slice). In order to obtain sensibly downsampled image stack, one could simply downsample each image by a factor 50/8. This can be achieved in any relevant image-processing application such as Fiji (Schindelin, et al. 2012) or Dragonfly (Reitbauer, et al. 2023). This would result in isotropic voxels with 50 nm resolution. This approach is straightforward but was found to be inferior to the method described in the next subsection (subSect. 4.4).

### Fibre vector field downsampling

**Fig. 11:**
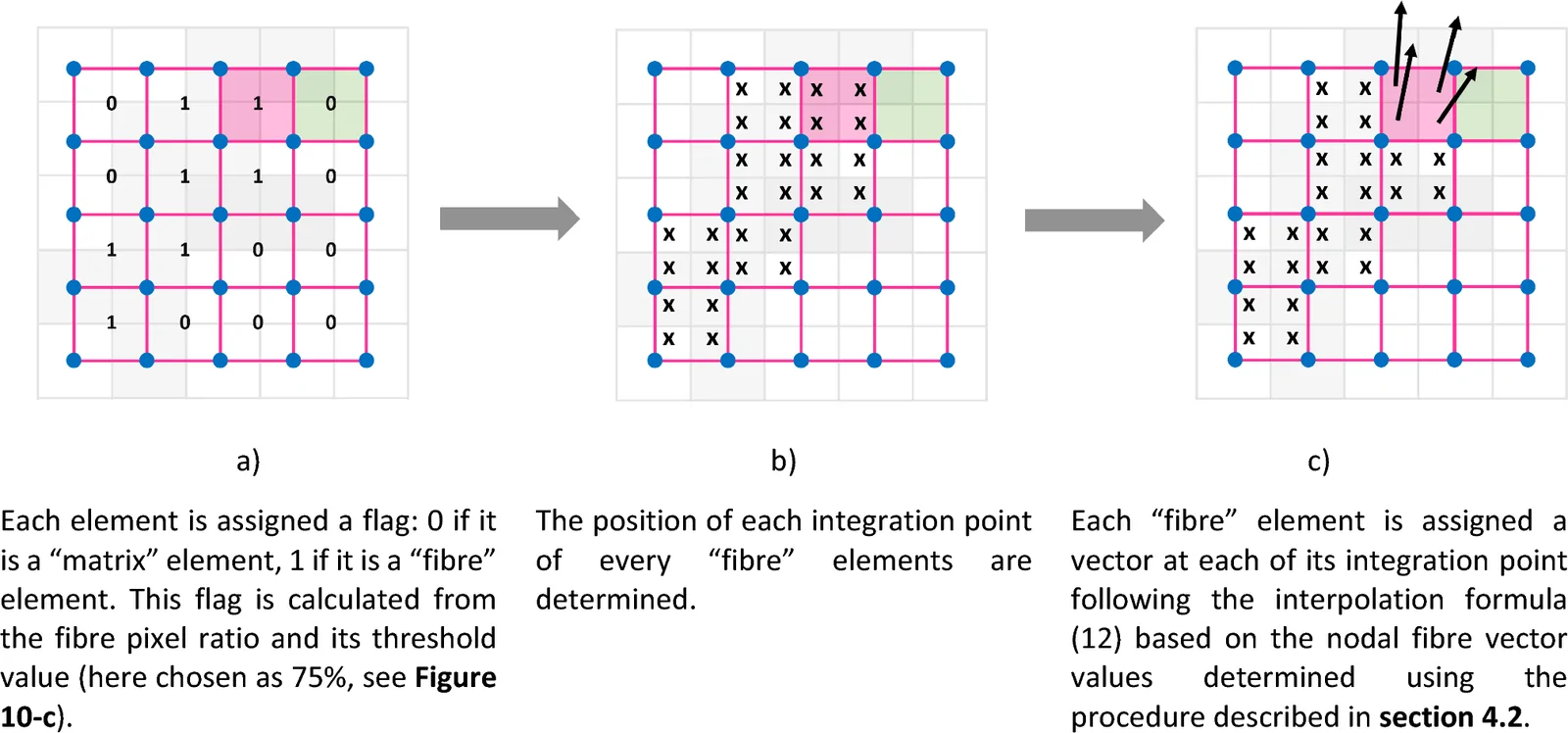
Schematic representation of the assignment of fibre vectors at integration point level. For sake of clarity, the schematic is restricted to 2D: pixels can be viewed as voxels with zero third dimension

**Fig. 12:**
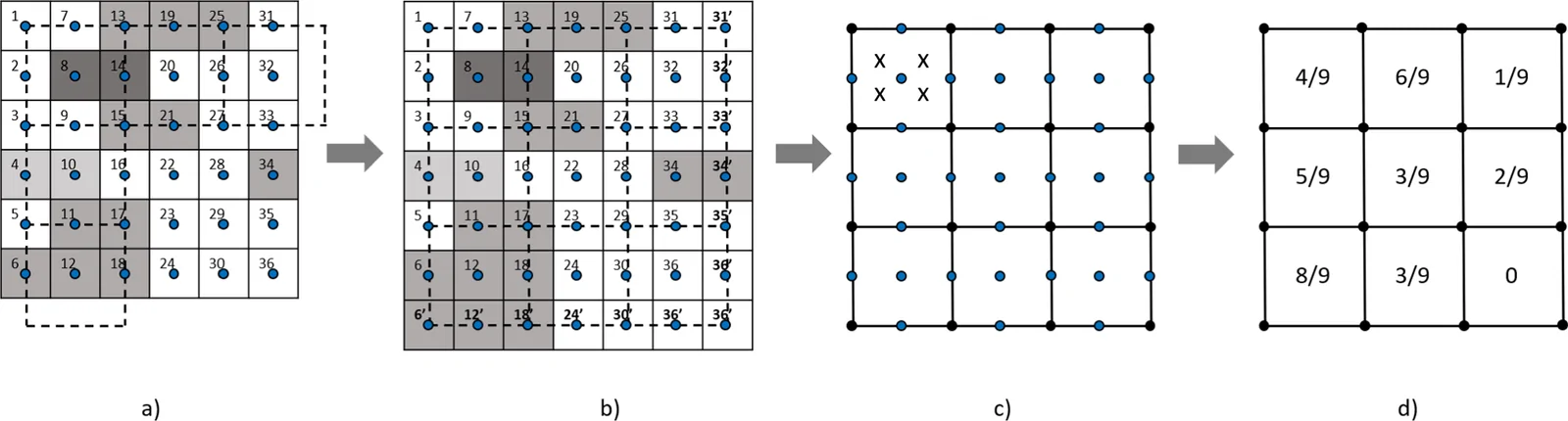
Schematic illustration of the downsampling procedure of the extracted fibre vector field. In this example, the input image is made of 6 × 6 pixels and the fibre phase is represented by grey shades). **a** From the structure tensor extraction procedure, a unique fibre vector is defined at the centroid of each voxel (3D)/pixel (2D). The position of each voxel/pixel centroid becomes the position of a node (blue disc) forming with all other nodes a structure grid; **b** when the number of voxels/pixels along one spatial dimension is an even number ghost nodes are created (denoted by the superscript prime symbol) to enable the creation of quadratic interpolation element boundaries, effectively simulating the creation of an additional row or column of voxels/pixels. For every 9-/27-noded quadrilateral/hexahedral elements quadratically interpolated on can calculate the value of the fibre vector field at every location in that quadrilateral/hexahedral element (see Eq. (12) and Appendix for an explicit definition of interpolation functions). For the 27-node hexahedral elements, the nodes lying in the mid-plane parallel to a pair of images have their fibre vector values calculated by way of linear interpolation between the corresponding nodes that lay on both the axis normal to the images’ plane and the images’ planes; **c**) the value of the fibre vector field is then calculated by interpolation at every integration point of the newly defined and linearly interpolated 4-/8-noded quadrilateral/hexahedral (black nodes);**d**) by counting the number of blue nodes inside the fibre phase, $n^{f}$ (see Fig. 12-a, Fig. 12-b) for every quadratic element one can determine the fibre pixel ratio as $r^{f} = n^{f} /9$. The fibre pixel ratio of every one of the 9 linear elements is indicated inside (0 to 8/9)

The idea behind this method is to first conduct a structure tensor analysis on the full-resolution dataset and then downsample the resulting fibre vector field (Fig. 11). This is achieved by first resampling the vector field from a linear to a quadratic Lagrange interpolation, utilising the shape functions of 8- and 27-noded hexahedral finite (see Sect. 14. Supplementary material 2—Finite element Lagrange shape functions). The principle of the procedure is illustrated in Fig. 12 but restricted to 2D for sake of clarity and easy visualisation. This approach enables the calculation of vector values at each integration points of a linear 4-noded quadrilateral element, itself composed of four 4-noded linear quadrilaterals corresponding to full-resolution pixels (see subSect. 4.2).

Similarly to the procedure described in Sect. 4.3, our Python application enables the export of the downsampled voxel-based mesh with integration point level fibre vector assignment to the VTK and Abaqus® file formats.

## Constitutive modelling

In the section, the basic kinematics and constitutive equations relevant to the modelling of continuum fibre-reinforced biological composites are summarised for sake of completeness.

### Finite deformation kinematics and invariant-based transversely isotropic hyperelasticity

Let us consider a continuum three-dimensional body composed of material particles defined by their *material* and *spatial* positions, respectively, denoted by ${\boldsymbol{X}}$ and ${\boldsymbol{x}} = {\boldsymbol{\chi}}({\boldsymbol{X}},t)$ where ${\boldsymbol{\chi}}$ is the motion and t, the time. The deformation of the body is fully characterised by the deformation gradient ${\boldsymbol{F}}$ (Marsden and Hughes 1994), *i.e.* the material gradient of the motion, defined by ${\boldsymbol{F}}({\boldsymbol{X}},t): = \partial {\boldsymbol{\chi}}({\boldsymbol{X}},t)/\partial {\boldsymbol{X}}$. ${\boldsymbol{F}}$ maps infinitesimal line vectors from the material to the spatial configuration while ${\boldsymbol{F}}^{ * } {\text{ = cofactor}}\left( {\boldsymbol{F}} \right) = J\,{\boldsymbol{F}}^{ - T}$ and $J = {\mathrm{determinant}}\left( {\boldsymbol{F}} \right)$, respectively, maps oriented infinitesimal surface and volume. The right (material) and left (spatial) Cauchy–Green deformation tensors are, respectively, defined as ${\boldsymbol{C}} = {\boldsymbol{F}}^{{\mathrm{T}}} .{\boldsymbol{F}}$ and ${\boldsymbol{b}} = {\boldsymbol{F}}.{\boldsymbol{F}}^{{\mathrm{T}}}$ while the Green–Lagrange strain tensor is defined as ${\boldsymbol{E}} = \,({\boldsymbol{C}} - {\boldsymbol{I}})/2$ where ${\boldsymbol{I}}$ is the second-order identity tensor.

The classical three principal deformation invariants of ${\boldsymbol{C}}$ which encapsulate the isotropic hyperelastic response of a material are defined as follows (Holzapfel 2000; Marsden and Hughes 1994):

12 $$I_{1} = {\mathrm{trace}}\left( {\boldsymbol{C}} \right) = {\boldsymbol{C}}:{\boldsymbol{I}};\,\,\,\,\,\,I_{2} = \frac{1}{2}[I_{1} - {\boldsymbol{C}}^{2} :{\boldsymbol{I}}];\,\,\,\,\,\,I_{3} = {\mathrm{determinant}}\left( {\boldsymbol{C}} \right)$$

where the symbol “:” denotes the dot product of two second-order tensors.

To characterise transversely isotropic material symmetry, one introduces a unit vector ${\boldsymbol{n}}_{0}$ defined in the reference configuration and associated with the principal material direction orthogonal to the plane of material isotropy. Because this unit vector has a sense, one introduces the concept of structure tensor ${\mathbf{\mathcal{S}}}_{0} = {\boldsymbol{n}}_{0} \otimes {\boldsymbol{n}}_{0}$ (Boehler 1978; Holzapfel 2000; Humphrey and Yin 1987; Limbert and Taylor 2002; Spencer 1992; Weiss, et al. 1996) which is an even functions of ${\boldsymbol{n}}_{0}$. The symbol “⊗”denotes the tensor outer product such that, in index notation, $\left( {{\mathbf{\mathcal{S}}}_{0} } \right)_{ij} = ({\boldsymbol{n}}_{0} )_{i} ({\boldsymbol{n}}_{0} )_{j}$. The general formulation used in this paper is based on the constitutive equations developed by Gasser, Ogden, and Holzapfel in their seminal paper on orthotropic hyperelasticity with fibre splay which introduced the concept of generalised structure tensor (Gasser, et al. 2006). From now on, the two-family of fibres hyperelastic model with fibre dispersion will be referred as the GOH model (Gasser, et al. 2006). Here, we particularise this approach to the case of transverse isotropy as only one family of fibres is considered and introduce the generalised structure tensor ${\boldsymbol{H}}$:

13 $${\boldsymbol{H}} = \kappa \,{\boldsymbol{I}} + (1 - 3\kappa )({\boldsymbol{n}}_{0} \otimes {\boldsymbol{n}}_{0} ) = \kappa \,{\boldsymbol{I}} + (1 - 3\kappa )\,{\mathbf{\mathcal{S}}}_{0}$$

where κ is a constitutive parameter that characterises the amount of fibre dispersion around the mean direction $\pm {\boldsymbol{n}}_{0}$. Depending on the value of κ, one recovers pure isotropy (κ=1/3) or transverse isotropy without fibre dispersion (κ=0). The spatial counterpart of ${\boldsymbol{H}}$, ${\boldsymbol{h}}$, is obtained by push-forward operation $\varphi_{*}$:

14 $$h = \varphi _{*} H = F.H.F^{T} = \kappa \,b + (1 - 3\kappa )\,\lambda ^{2} (n \otimes n)$$

where ${\boldsymbol{n}} = {\boldsymbol{F}}.{\boldsymbol{n}}_{0} /\lambda$ is the unit vector along the fibre in the *deformed* configuration and $\lambda = \sqrt {{\boldsymbol{n}}_{0} .{\boldsymbol{C}}.{\boldsymbol{n}}_{0} } = \sqrt {{\boldsymbol{C}}:{\mathbf{\mathcal{S}}}_{0} } = \sqrt {I_{4} }$ is the stretch along the fibre direction which is an anisotropic invariant of ${\boldsymbol{C}}$ and its tensor agency, ${\mathbf{\mathcal{S}}}_{0}$ (Holzapfel 2000). The normalised spatial counterpart of ${\mathbf{\mathcal{S}}}_{0}$ is ${\mathbf{\mathcal{S}\ominus }} = {\boldsymbol{n}} \otimes {\boldsymbol{n}}$.

The existence of a strain energy function $\psi = \psi (I_{1} ,I_{4} )$ is postulated. It follows that the second Piola–Kirchhoff stress tensor—obtained by differentiation of ψ with respect to ${\boldsymbol{C}}$—is expressed as follows:

15 $${\boldsymbol{S}} = 2\frac{\partial \psi }{{\partial {\boldsymbol{C}}}} = 2\left( {\frac{\partial \psi }{{\partial I_{1} }}{\boldsymbol{I}} + \frac{\partial \psi }{{\partial I_{4} }}{\mathbf{\mathcal{S}}}_{0} } \right)$$

The first Piola–Kirchhoff, also known as the nominal stress tensor, ${\boldsymbol{P}}$, is obtained from the second Piola–Kirchhoff as (Holzapfel 2000):

16 $${\boldsymbol{P}} = {\boldsymbol{F}}.{\boldsymbol{S}} = 2\left( {\frac{\partial \psi }{{\partial I_{1} }}{\boldsymbol{F}} + \frac{\partial \psi }{{\partial I_{4} }}{\boldsymbol{F}}.{\mathbf{\mathcal{S}}}_{0} } \right)$$

Following standard usage in the finite element implementation of nearly incompressible materials and to avoid element locking (Simo and Taylor 1991), the elastic strain energy density is split into deviatoric and volumetric functional components. The tensor invariant arguments of these functions are modified invariants defined as:

17 $$\bar{I}_{1} = J^{{ - 2/3}} I_{1} ;\,\,\bar{I}_{4} = J^{{ - 2/3}} I_{4}$$

An additive decomposition of ψ is further assumed so it takes the following form:

18 $$\psi = \psi (\boldsymbol{\overline{C},H},J) = \overline{\psi}_{g} (\overline{\boldsymbol{C}}) + \overline{\psi}_{f} (\overline{\boldsymbol{C}},\boldsymbol{H}) + U(J)$$

where $\overline{\psi}_{g} (\overline{\boldsymbol{C}})$, $\overline{\psi}_{f} (\overline{\boldsymbol{C}},\boldsymbol{H})$, and U(J) are, respectively, the strain energy function associated with the non-collagenous isotropic isochoric matrix in which collagen fibres are embedded, the elastic energy stored in the incompressible fibres, and the isotropic and volumetric strain energy stored in the matrix. For a fully incompressible material J=1 which leads to:

19 $$\psi = \psi (\boldsymbol{\overline{C},H}) = \psi (\boldsymbol{C,H}) = \overline{\psi}_{g} (\boldsymbol{C}) + \overline{\psi}_{f} (\boldsymbol{C},\boldsymbol{H})$$

In its original form, the GOH model (Gasser, et al. 2006) was designed to describe the anisotropic elastic properties of arterial tissues by accounting for collagen fibre dispersion around two main directions corresponding to two individual fibre families.

This constitutive model was used to identify the anisotropic properties of human skin by Ní Annaidh et al. (Ní Annaidh, et al. 2012a, 2012b) and was also implemented in Abaqus/Standard for simulated extension tests (Ní Annaidh, et al. 2012a).

Here, we particularise the GOH model to a single family of fibres and define the following strain energy functions (Gasser, et al. 2006):

20 $$\overline{\psi}_{g} (\overline{\boldsymbol{C}}) = \frac{\mu }{2}(\overline{I}_{1} - 3)$$

21 $$\overline{\psi}_{f} (\overline{\boldsymbol{C}},\boldsymbol{H}) = \frac{{k_{1} }}{{2k_{2} }}\left\{ {\exp \left[ {k_{2} \left\langle {\boldsymbol{H}:\overline{\boldsymbol{C}} - 1} \right\rangle^{2} } \right] - 1} \right\} = \frac{{k_{1} }}{{2k_{2} }}\left\{ {\exp \left[ {k_{2} \left\langle {\kappa \overline{I}_{1} + (1 - 3\kappa )\overline{I}_{4} - 1} \right\rangle^{2} } \right] - 1} \right\}$$

where <> denotes the Macaulay bracket defined as:

22 $$\left\langle \bullet \right\rangle = \left\{ {\frac{{ \bullet + \bullet }}{2}, \bullet \ge 0;\,\,\frac{{ \bullet - \bullet }}{2}, \bullet < 0} \right\}$$

and μ, $k_{1}$, and $k_{2}$ are material parameters, respectively, corresponding to the ground-state shear modulus of the non-collagenous matrix, a stress-like and dimensionless factors capturing the mechanical response of individual fibres in a homogenised sense. The term $\boldsymbol{H}:\overline{\boldsymbol{C}}$ is a deviatoric Green–Lagrange strain-like quantity that captures the extension *along* and *around* the main fibre direction ${\boldsymbol{n}}_{0}$.

The distribution orientation of a single family of fibres can be described by a π-periodic von Mises distribution centred at $\theta = 0^{ \circ }$ which obeys the following density function $\overline{\rho } (\theta )$:

23 $$\rho (\theta ) = \frac{\exp (b\cos (2\theta ))}{{2\pi I_{0} (b)}}, \, I_{0} (b) = \frac{1}{\pi }\int_{0}^{\pi } {\exp (b\cos \theta )} \,{\mathrm{d}}\theta$$

where $I_{0} (x)$ is the modified order-zero Bessel function of the first kind and b is a concentration parameter that characterises fibre dispersion. In order to satisfy the normalisation condition, a modified π-periodic von Mises distribution $\overline{\rho }(\theta )$ (Gasser, et al. 2006) can be defined as:

24 $$\overline{\rho }(\theta ) = 4\sqrt {\frac{b}{2\pi }} \frac{{\exp \{ b[\cos (2\theta ) + 1]\} }}{{{\mathrm{erfi}}(\sqrt {2b} )}},{\text{ erfi}}(x) = - i\frac{2}{\sqrt \pi }\int_{0}^{ix} {e^{ - t} } {\mathrm{d}}t$$

where ${\mathrm{erfi}}$ is the imaginary error function.

The fibre dispersion parameter κ of the structure tensor approach of Gasser et al. (Gasser, et al. 2006) can be expressed as (Ogden 2016):

25 $$\kappa = \frac{1}{4}\int_{0}^{\pi } {\rho (\theta )\sin^{3} \theta } \,{\mathrm{d}}\theta = \frac{1}{2} + \frac{1}{8b} - \frac{1}{4}\sqrt {\frac{2}{\pi b}} \frac{\exp (2b)}{{{\mathrm{erfi}}(\sqrt {2b} )}}$$

### Finite element implementation of constitutive equations

Although Abaqus® provides a built-in GOH material model in its library, this proprietary implementation only allows assignment of mean fibre directions at element (centroid) level, not at integration point level. For sake of flexibility and in order to also implement additional arbitrary constitutive laws for both matrix and fibres, a generalised transversely isotropic hyperelastic material with fibre dispersion was implemented as a modular Abaqus/Standard user material subroutine UMAT coded in Fortran. An open-source Python library (https://codebase.helmholtz.cloud/ingo.scheider/abaqus_plugins) was used to convert Abaqus® result files (ODB) into VTK unstructured mesh data files (VTU) in order to enable the post-processing of finite element results through the advanced visualisation algorithms available in ParaView.

## Finite element modelling of skin micromechanics

### Multiscale modelling of fibrous soft tissues

In the multiscale modelling of biological soft tissues, several theoretical/numerical frameworks have been used, each offering distinct advantages for representing fibrous microstructure and linking microscale behaviour to macroscopic tissue response (Dalbosco, et al. 2024). The RVE framework of FE^2^ methods (Kouznetsova, et al. 2001; Šolinc and Korelc 2015) that couples micromechanical problems to integration points of meso-/macroscopic finite elements has emerged as the predominant multiscale approach for fibrous tissues, providing a natural bridge between microscale fibre mechanics and macroscopic tissue behaviour. (Dalbosco, et al. 2021) demonstrated that RVEs with embedded collagen fibres in arterial tissue require careful consideration of boundary conditions, finding that periodic boundary conditions show fastest convergence and reduced boundary artefacts compared to uniform displacement or traction conditions. Their work revealed considerable microscopic strain heterogeneity, with local strains significantly exceeding macroscopic values—a finding critical for understanding mechanotransduction mechanisms. The RVE size determination remains a critical challenge requiring convergence studies. (Dalbosco, et al. 2024) comprehensively reviewed this issue for arterial tissues, emphasising that scale separation assumptions must be validated and that optimal RVE size depends on fibre dispersion, with highly dispersed architectures requiring larger volumes to achieve statistical representativeness. Embedded element methods represent discrete fibres as one-dimensional (1D) elements within a 3D ground matrix, avoiding the need for conforming meshes between constituents. This technique has proved computationally efficient while preserving essential microstructural details. (Dalbosco, et al. 2021) implemented embedded elements with stiffness correction methods to address redundancy issues arising from independent fibre-matrix discretisation. Their approach successfully modelled out-of-plane fibre distributions using probability density functions, crucial for capturing 3D dispersion in arterial walls. Multiple recent studies employed embedded elements across tissue types. Brain white matter models used this technique to couple axonal fibres with extracellular matrix, implementing recruitment stretch concepts rather than geometric waviness to represent fibre tortuosity—a computationally advantageous choice (Budday, et al. 2020; Mazhari and Shafieian 2024). The two-scale numerical study of abdominal aortic aneurysms by (Dalbosco, et al. 2023) demonstrated that embedded element RVEs can link macroscale tissue mechanics to microscale cellular mechanotransduction, showing 80% strain amplification from macro- to microscales and revealing how diseased tissue establishes new homeostatic states. Experimental validation for structure tensor parameters emerged from multiphoton microscopy-based studies. (Pukaluk, et al. 2023) tracked real-time microstructural changes in human aortic adventitia under biaxial loading, showing marked decreasing in collagen dispersion during stretch and division from one to two fibre families under equibiaxial loading. Multimodal experimental studies increasingly complement computational approaches. (Pukaluk, et al. 2024) reviewed experimental methods for human aortas, emphasising integration of mechanical testing with structural characterisation to validate multiscale models.

In the context of skin biomechanics, Dwivedi et al. (Dwivedi, et al. 2022) conducted an integrative study combining multimodal imaging, physical characterisation (in vivo for human skin and in vitro for porcine skin) and multiscale modelling using a RVE consisting of idealised collagen fibrils embedded in a soft ground matrix. Both phases were assumed to be linear elastic with a 1.5 GPa Young’s modulus for fibrils and 140 kPa modulus for the matrix. The study demonstrated that stress relaxation in skin is driven by interstitial fluid efflux from the tissues and also evidenced auxetic behaviour due to the large rotation of stiff collagen fibres together with their bending and straightening causing large expansion against the low stiffness matrix. Structure tensor approaches have been integrated with mesoscale RVE models to model skin. (Moreno-Flores, et al. 2024) employed the GOH model with fibre orientations aligned to Langer lines, demonstrating that while sinusoidal epidermis-dermis interfaces do not affect homogenised response, they create stress concentrations at rete ridge valleys with implications for mechanosensing. (Sohutskay, et al. 2021) developed a mechanobiological wound model using structure tensors with von Mises distributions, showing that fibre dispersion critically controls wound contraction patterns as scaffolds with increased isotropy ($\kappa \to$ 0.3) exhibited reduced and more uniform contraction compared to the case of aligned configurations.

A fundamental methodological distinction exists between continuum fibre-reinforced models employing generalised structure tensors and direct fibre models explicitly representing individual fibres. (He, et al. 2024) conducted a systematic comparison for the biomechanics of optic nerve head, constructing both a continuum model based on the GOH formulation with fibre dispersion and a direct fibre model with explicit 3D collagen fibre bundles. Despite identical geometry, structural specifications, and boundary conditions, the models diverged substantially in capturing tissue-level and fibre-level mechanics. The direct fibre model accurately replicated experimentally observed depth-dependent radial strain variability, ring-like meridional strain patterns, and radial circumferential strain patterns, whereas the continuum model failed to reproduce these features (He, et al. 2024). Critically, the continuum model disrupted strain transmission along individual fibres, exhibiting high standard deviations in fibre element strains along each fibre trajectory. In contrast, the fibre model maintained smooth strain distributions along fibre paths, consistent with continuous fibre transmission of mechanical loads. The authors attributed these discrepancies to inherent limitations of homogenisation schemes and assumptions of affinity of deformations in continuum frameworks, which cannot capture complex non-affine kinematics and fibre–fibre interactions spanning multiple finite elements (He, et al. 2024).

Here, by design, our approach is restricted to only solving the *micromechanical* problem of voxel-level collagen fibres embedded in the ground substance of the matrix. The ambition is to understand and quantify the effects of non-uniform fibre orientation distribution on the mechanical response of the RVE compared to the response obtained from uniform distributions. Therefore, we depart from classical multiscale models (Kouznetsova, et al. 2001; Šolinc and Korelc 2015) which use this local micromechanical problem to inform constitutive behaviour at the integration points of a macroscopic finite element mesh. As a consequence, we will not consider periodic boundary conditions for our RVE.

In the next sections, we present the methodologies to explore the mechanical response of an RVE containing oriented collagen fibres extracted from SBF-SEM images and assess the influence of modelling assumptions about the spatial distribution of fibre volume fraction and orientation (Sect. 6.2), and that of the vector interpolation algorithm in relation to the fibre pixel ratio (Sect. 6.3).

### Homogeneous versus heterogeneous fibre volume fraction and orientation.

**Fig. 13:**
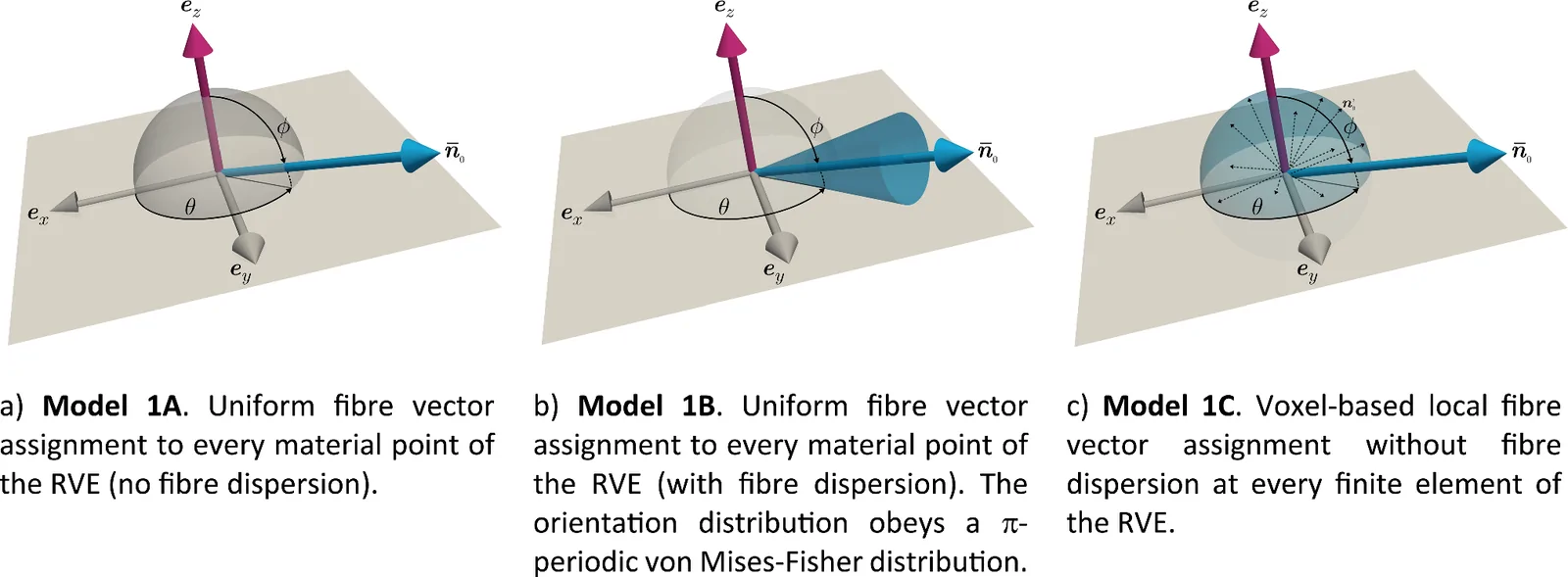
Schematics representing the three distinct conceptual modelling approaches use to represent the mechanics of collagenous-rich human dermis. The differences lay in how fibre vectors are assigned to individual finite elements, at their individual integration points: **a**) and **b**: a mean fibre vector $\overline{\user2{n}}_{0}$ is uniformly applied to every element integration point; c): every element integration point i is assigned a unique local fibre vector ${\boldsymbol{n}}_{0}^{i}$. The preferred mean fibre direction for Models 1A and 1B is identical, with an azimuth angle of 134° and an inclination angle of 75°. Model 1B includes an extra fibre dispersion parameter κ.

**Fig. 14:**
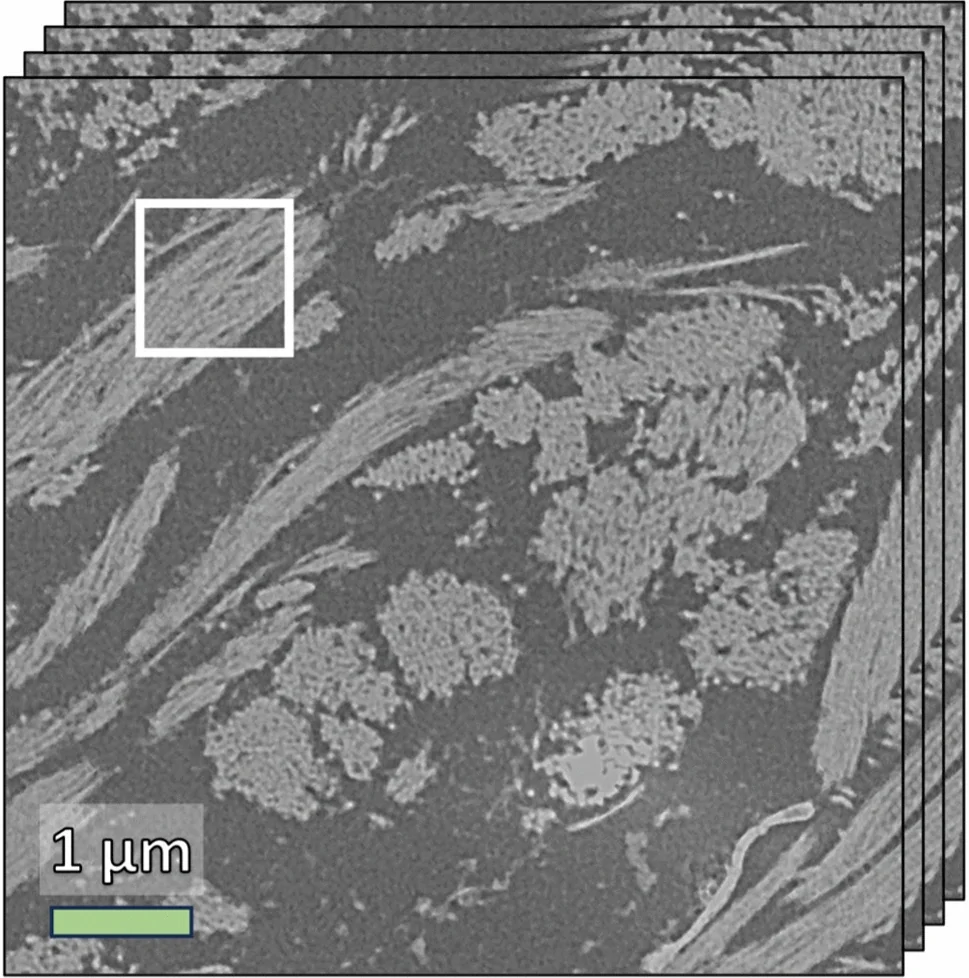
Selection of a volume of interest (VOI), denoted here by a white square. The 101 × 101x16 pixel ROI was extracted from the SBF-SEM images presented in Sect. 2

**Fig. 15:**
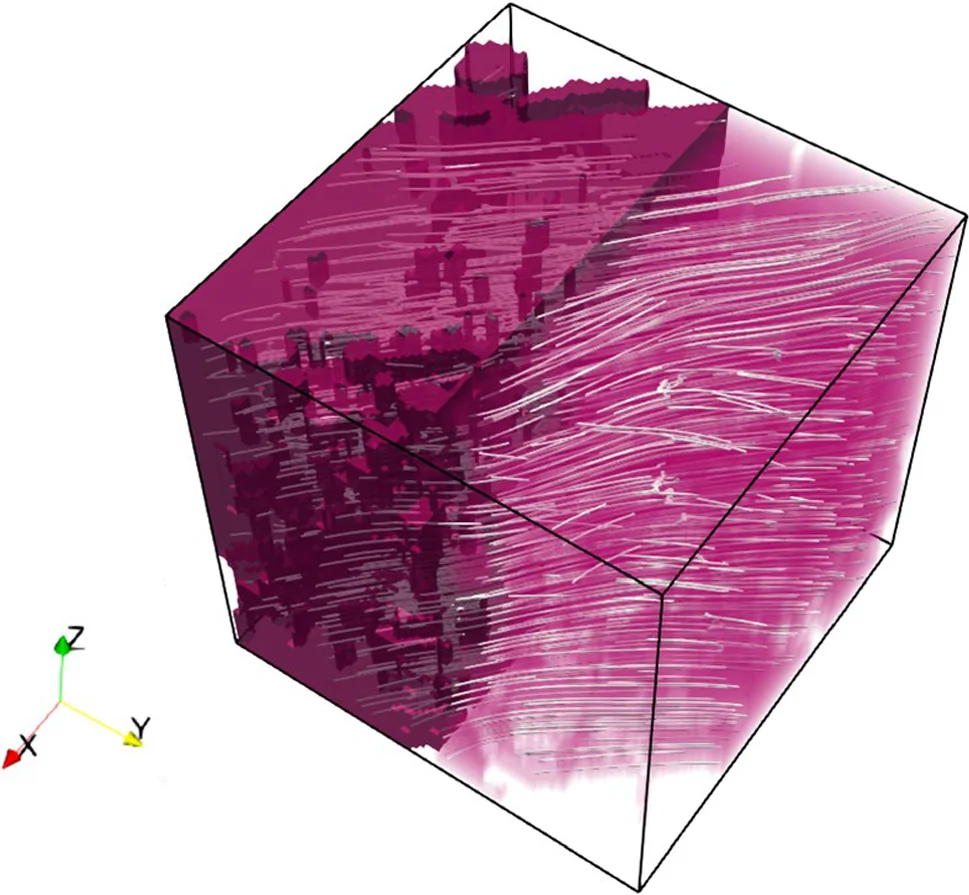
Visualisation of the extracted collagen fibre architecture using streamlines traced by plotting the tangents to the local fibre vectors in ParaView. The fibre extraction algorithm settings were σ=1.5 and $\, \rho = 6$ while the mesh dimensions were 100 × 100 × 16 (0.8 × 0.8 × 0.8 μm^3^). The fibre phase is coloured in mulberry.

**Fig. 16:**
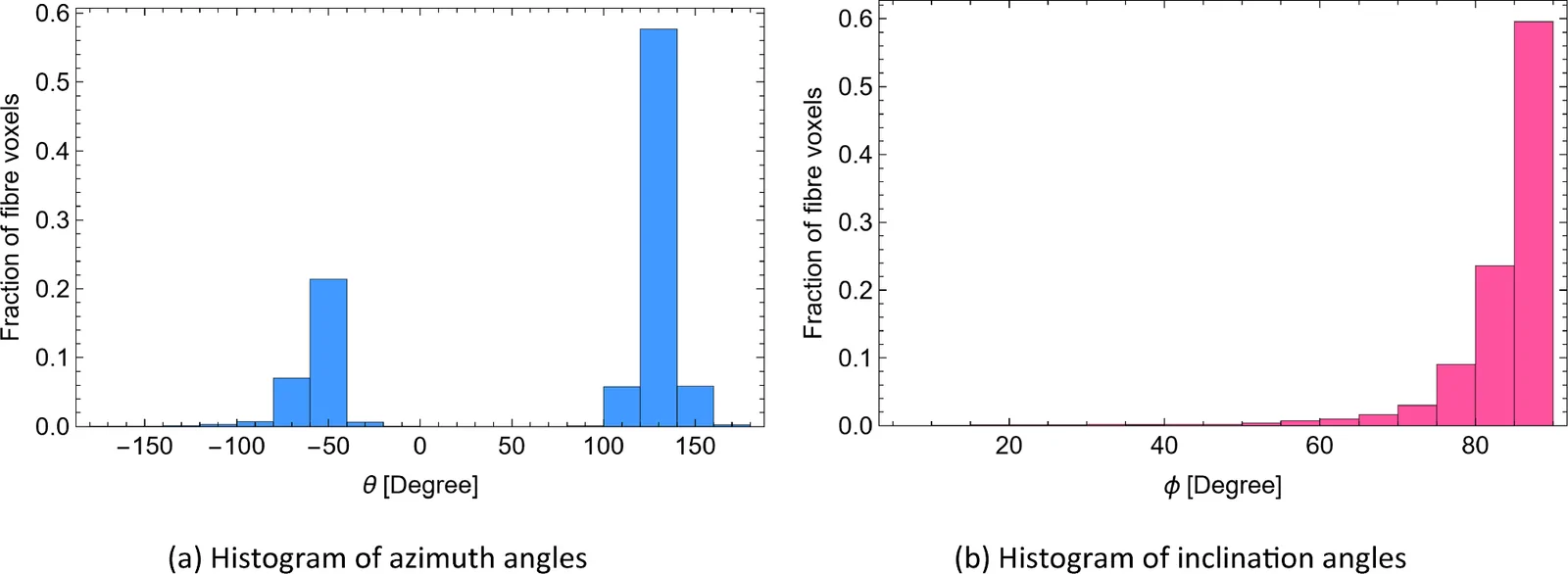
Histogram of the azimuth and elevation fibre angles extracted from the VOI presented in Fig. 14

Here, the objective was to compare the mechanical response of an image-based RVE of human dermis for three different cases and quantify the link between collagen fibre architecture in an RVE and the global mechanical response of such an assembly. In the first two models, model 1A and model 1B, it was assumed that the RVE was a collagen fibre-reinforced continuum, respectively, with a spatially *uniform* and π-periodic von Mises distributions of fibre orientation (Fig. 13-a-b). These distributions were assumed to be homogeneously distributed in the RVE. The third model, model 1C, accounted for the non-uniform local orientation of collagen fibres and also the distinct matrix and fibre phases, unlike the two other models (Fig. 13-c) which, by design, had unit volume fraction for both matrix and fibres. The RVE considered was extracted from the stack of SBF-SEM images described in Sect. 2. To incorporate a region containing continuously distributed fibres, connecting opposite faces of the RVE a smaller volume of interest (VOI) was identified (Fig. 14). Utilising the 3D structure tensor approach, voxel-based fibre vectors (Fig. 15) were extracted from the input image stack and the distributions of their azimuth and elevation angles were plotted as histograms (Fig. 16). A structured 8-noded hexahedral mesh was built as per the methodology described in Sect. 4**.** From the extracted discrete-fibre vector field, a mean fibre vector direction representative of the RVE was calculated for implementing models 1A and 1B which rely on the definition of a mean fibre direction $\overline{\user2{n}}_{0}$. Furthermore, model 1B requires the extraction of a concentration parameter b from the set of discrete-fibre directions. This was accomplished in Mathematica® by calculating the maximum likelihood estimate using the FindDistributionParameters function using the “MaximumLikelihood” option for the ParameterEstimator setting.

By means of Eq. (26), the dispersion parameter of the GOH model, κ, was determined. The statistical parameters of the collagen fibre architecture in the VOI are collected in Table 1. For model 1C, an ASCII file containing fibre vector components at each integration point of every finite element was written to be subsequently read within a UMAT user subroutine using the UEXTERNALDB subroutine that handles file operation in Abaqus®. Importantly for large-scale FE computations, the use of UEXTERNALDB is necessary for handling parallel file operations within a UMAT subroutine on multiple compute nodes, in contrast to multiple cores on a single compute node.

**Table 1:** Statistical parameters characterising the fibrous architecture of the collagen in the VOI

Mean azimuth angle θ (°)	Mean elevation angle ϕ (°)	Concentration parameter b	Dispersion parameter κ
134.05	75.04	5.6734	0.0467

Each of the three models was subjected to the same boundary and loading conditions which consisted of uniaxial tension along the three principal directions of the RVE and pseudo-homogeneous conditions on three lateral sides of the RVE. The underlying goal was to extract an equivalent homogenised stress–strain curve for the RVE. The constitutive parameters used for the three models are listed in Table 2.

**Table 2:** Constitutive parameters of the transversely isotropic hyperelastic model used in models 1A, 1b, and 1C (Ní Annaidh, et al. 2012b)

μ[kPa]	$k_{\,1}$[kPa]	$k_{\,2}$	κ	ν
201.4	49,061.04	0.1327		0.495

### Influence of interpolation method on RVE’s micromechanics

**Fig. 17:**
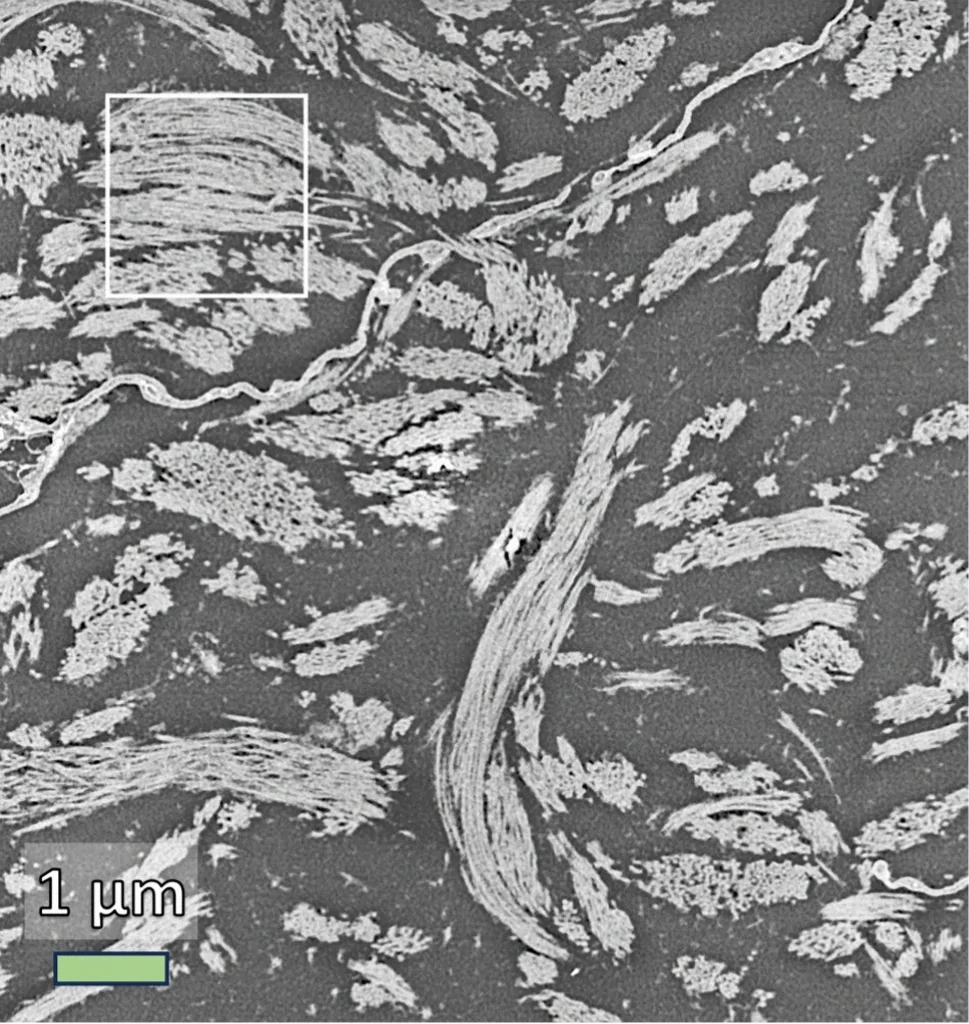
Selection of a volume of interest (VOI), denoted here by a white square (dimensions of VOI: 226 × 226x37 pixels, 1.808 × 1.808 × 1.85 μm^3^). The ROI was extracted from the SBF-SEM images presented in Sect. 2

**Fig. 18:**
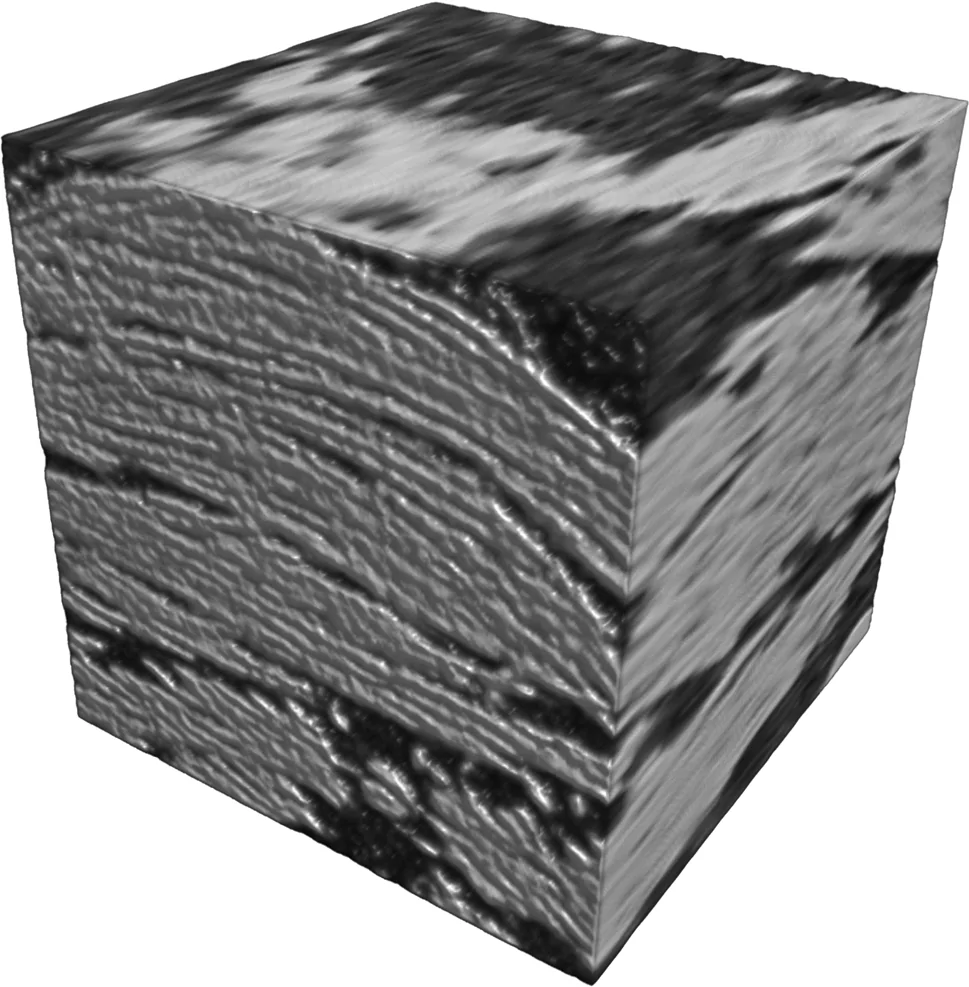
Volume rendering of the extracted collagen fibre architecture corresponding to the VOI described in Fig. 17. Visualisation was conducted in the software application Dragonfly (Comet Technologies Canada Inc., Montréal, Canada)

The fibre pixel ratio (see Sect. 4.2) inherently conditions the integration of fibres and their volume fraction into the RVE’s finite element mesh. It is therefore essential to understand the sensitivity of the mechanical response of the RVE to this parameter. In order to conduct a parametric analysis exploring these aspects, a new VOI was selected from the original dataset (see Fig. 17 for volume rendering of the VOI and Fig. 18 for the corresponding extracted fibre architecture).

From this image stack, two models were generated: model 2A: no downsampling of the fibre vector field was applied; model 2B: a 1/2 × 1/2 × 1 downsampling scheme was applied to the fibre vector field (see Sect. 4.4 for a description of the downsampling procedure). For model 2A and model 2B, the pixel ratio can, respectively, take the following values: 1/4, 1/2, 3/4, and 1, and 1/9, 2/9, 1/3, 4/9, 5/9, 2/3, 7/9, 8/9, and 1. Both models were subjected to the same uniaxial extension and pseudo-homogeneous boundary conditions and their mechanical responses compared. The constitutive parameters and formulation utilised in these analyses are identical to those outlined in Sect. 6.2.

**Fig. 19:**
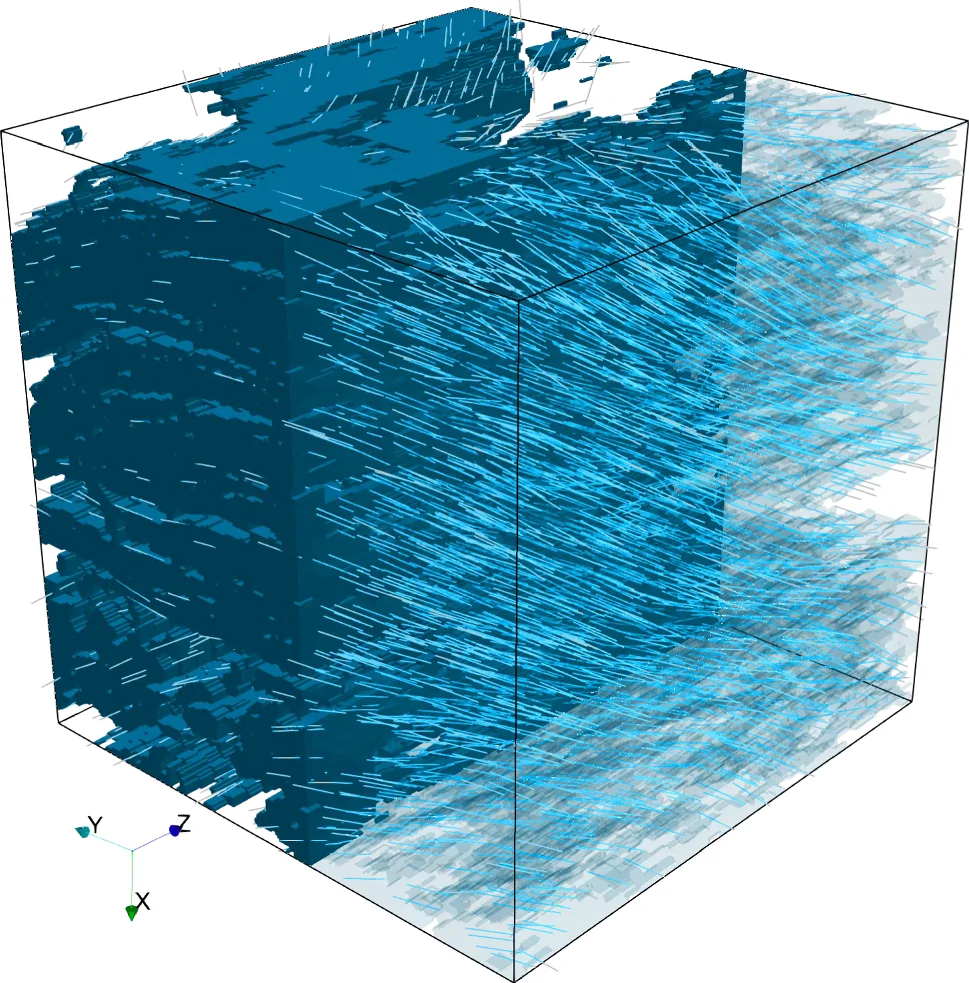
Visualisation of the extracted collagen fibre architecture using streamlines traced by plotting the tangents to the local fibre vectors in ParaView. The fibre extraction algorithm settings were σ=1.5 and $\, \rho = 6$ while the mesh dimensions were 225 × 225 × 36 (1.8 × 1.8 × 1.8 μm^3^). The fibre phase is coloured in mulberry

**Fig. 20:**
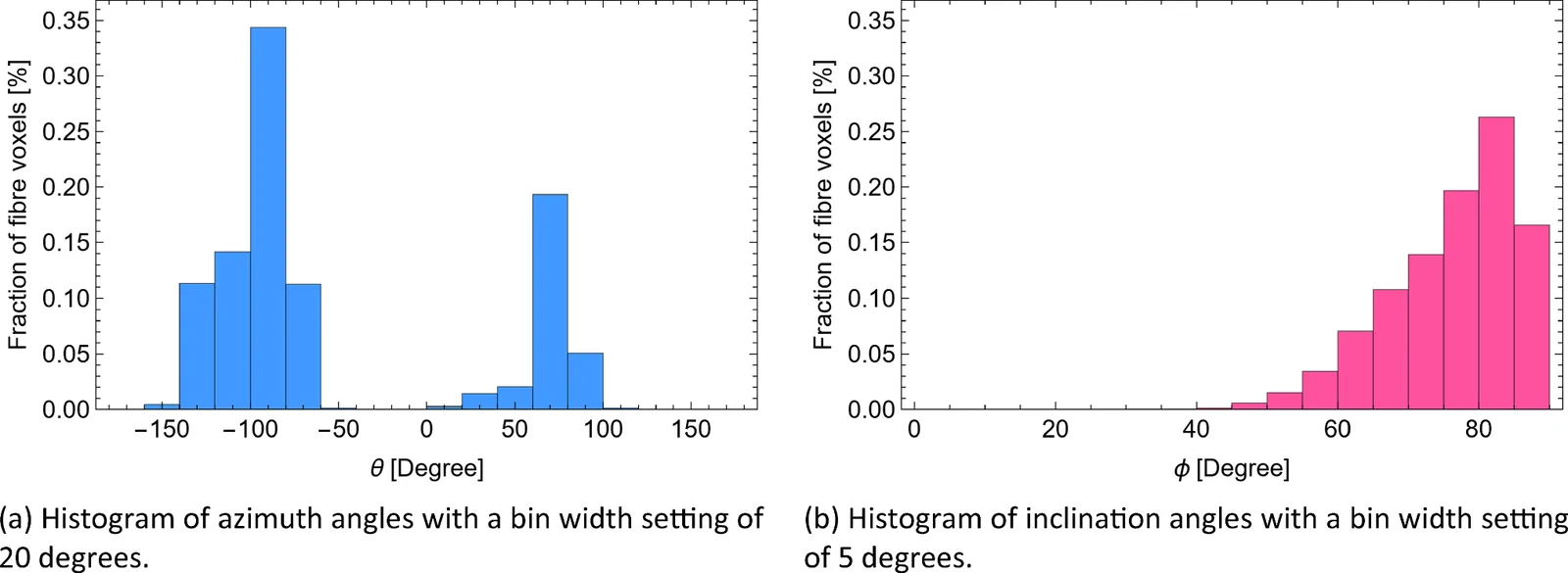
Histograms of azimuth and inclination fibre angles extracted from skin image subvolume shown in Fig. 17

By using 3D structure tensor analysis with input parameters σ=1.5 and ρ=6, the extracted fibre vectors (Fig. 19) were converted to azimuth and inclination angles that were then plotted as histograms (Fig. 20). There are two peak values of -90° and 70° in the histogram of azimuth angles and one peak value of 85° in the inclination angle histogram.

### The role of explicit fibre/fibre bundle unfolding on RVE mechanics

It is broadly recognised that the typical strain-hardening response of biological soft tissues is the macroscopic manifestation of multiple microstructural deformation mechanisms consisting of the progressive and sequential uncrimping of collagen and elastic fibres within a viscous solid matrix upon application of a macroscopic tensile load (Bancelin, et al. 2015; Fung 1981; Jayyosi, et al. 2017; Wahlsten, et al. 2023). Naturally, this is a simplified conceptual representation of what really is a complex cascade of multiphysics and multiscale mechanisms (Buehler 2006a, 2006b; Marino and Wriggers 2017). As collagen fibres progressively unfold, they align along the loading direction (Bancelin, et al. 2015; Jayyosi, et al. 2017; Krasny, et al. 2017) and stresses rise nonlinearly according to what is often approximated as an exponential function. This microstructural aspect of continuum fibre-reinforced constitutive models of biological tissues of the GOH-type (Gasser, et al. 2006) is *intrinsically* captured through the use of an exponential function of the trace of the generalised structure tensor, ${\boldsymbol{H}}:{\boldsymbol{I}}$, or that of its deviatoric counterpart $\overline{\user2{H}}:{\boldsymbol{I}}$ (see Eq. (22)). One could argue that the approach presented in Sect. 3 that captures *local* fibre orientation within an RVE unlike conventional GOH-type modelling that assumes spatially uniform mean vector orientation within an RVE should not use an exponential function for the fibre strain energy. Indeed, this would effectively amount to “double-counting” the microstructural unfolding mechanisms of the collagen fibres. The macroscopic exponential behaviour should arise from purely structural mechanism associated with local fibre deformation rather than through the nonlinear material behaviour of individual fibres. To test this hypothesis, it is proposed here to use a very simple quadratic strain energy functional form featuring a single elastic modulus-like constitutive parameter a to represent the mechanical response of (dispersed or not) collagen fibre bundles:

26 $$\bar{\psi }_{f}^{2} (\bar{\boldsymbol{C}},\boldsymbol{H}) = \frac{1}{2}a\left\langle {\boldsymbol{H}:\bar{\boldsymbol{C}} - 1} \right\rangle ^{2}$$

By using a such a strain energy function to model the material behaviour of fibres in combination with an RVE featuring spatially non-uniform local fibre orientation at element or element integration point level, one could segregate the nonlinear mechanical behaviour of fibres arising from geometric effects. The constitutive parameter a of $\bar{\psi }_{f}^{1}$ can be approximated from the GOH fibre strain energy function $\overline{\psi}_{f}$ as:

27 $$a = \left. {\frac{{\partial \overline{\psi}_{f} (\overline{\boldsymbol{C}},\boldsymbol{H})}}{\partial \lambda }} \right|_{{\lambda = \lambda^{*} }} = \left. {\frac{{\partial \frac{{k_{1} }}{{2k_{2} }}\left\{ {\exp \left[ {k_{2} \left\langle {\kappa \overline{I}_{1} + (1 - 3\kappa )\overline{I}_{4} - 1} \right\rangle^{2} } \right] - 1} \right\}}}{\partial \lambda }} \right|_{{\lambda = \lambda^{*} }}$$

where $\lambda^{*}$ is the stretch along the mean fibre direction at which the mechanical behaviour of the fibres can be assumed to be linear as they are fully taut. This typically corresponds to the part of the stress–strain curve that follows the heel region (Pissarenko, et al. 2019). After algebraic manipulation, one obtain the equivalent elastic modulus-like constitutive parameter a:

28 $$a = \frac{{2\,k_{1} \left( {\lambda^{*} - 1} \right)}}{{\lambda^{*3} }}\left( {\kappa - \lambda^{*3} + 2\,\kappa \lambda^{*3} } \right)\left[ {2\kappa \left( {1 + \lambda^{*} + \lambda^{*2} } \right) - \lambda^{*} \left( {1 + \lambda^{*} } \right)} \right]\exp \left[ {k_{2} \left\langle {\kappa \,(\lambda^{*2} + \frac{2}{{\lambda^{*} }}) + (1 - 3\kappa )\,\lambda^{*2} - 1} \right\rangle^{2} } \right]$$

Importantly, and as rightly pointed out by Wahlsten et al. (Wahlsten, et al. 2023), the concept of elastic modulus applied to biological soft tissues is not unambiguously defined. Indeed, the strict definition of elastic modulus would be the slope of the stress–strain curve in the limit of vanishing strains, and for a stress-free reference configuration. The existence of such a mechanical configuration is not guaranteed and, in any case, would remain challenging to characterise for any in vivo biological sample, particularly in the light of structural heterogeneities. Even ex vivo, a stress-free configuration is not guaranteed as skin is a multiscale and multiphase structure subject to complex residual strain patterns (Flynn 2019, Flynn, et al. 2013, Ní Annaidh and Destrade 2019) and mechanical processes (e.g. effect of osmotic pressure, (Sachs, et al. 2024; Wahlsten, et al. 2023)).

Another critical point raised by Wahlsten et al. (Wahlsten, et al. 2023) is that the definition of the elastic modulus as the tangent stiffness in the post-heel linear part of the strain–stress curve—where stress is in the 5-15 MPa range—generally means that loading conditions are well beyond physiological in vivo tension conditions. By considering the GOH constitutive parameters identified by (Ní Annaidh, et al. 2012b), the critical post-heel region stretch at which the skin stress–strain response becomes linear is approximately $\lambda^{*} =$ 1.925. This level of extension is far beyond physiological conditions. If one injects this stretch value into Eq. (29) and replaces the constitutive parameters by those listed in Table 2, the corresponding tensile elastic modulus-like parameter is obtained as a = 1350.04 MPa which is within the upper range/order of magnitude of values collagen fibre’s elastic modulus reported in the literature (Marino and Wriggers 2017) and references therein. Using optical tweezer characterisation techniques (Dutov, et al. 2016) showed that single type I collagen fibres extracted from rat tail tendons exhibited a tensile modulus in the 100–360 MPa range. In the light of findings of (Dutov, et al. 2016) and those of (Wahlsten, et al. 2023), it seems more appropriate to consider collagen fibre’s equivalent elastic modulus within the 25–500 MPa range. Accordingly, we propose to run model 1C using the constitutive Eq. (34) for the range of values $a = \{ 25,50,100,200,300,400,500\}$[MPa] which are listed in Table 3 and for the same constitutive parameters $\{ \mu ,\nu ,\kappa \}$ listed in Table 2. Additional pairs $\{ a> 500\,[MPa],\lambda ^{*} \}$ are provided for the simple purpose of comparison with regards to the level of equivalent stretch $\lambda^{*}$.

**Table 3:** Elastic fibre threshold stretch $\lambda^{*}$ calculated from the elastic tensile modulus of collagen fibres a using Eq. (29)

a[GPa]	0.025	0.05	0.075	0.1	0.2	0.3	0.4	0.5	0.6	0.7	0.8	0.9	1
$\lambda^{*}$	1.108	1.191	1.257	1.313	1.470	1.570	1.642	1.670	1.741	1.777	1.810	1.836	1.860

One can define a *fully incompressible form* of the total strain energy function, $\psi^{[2a]}$:

29 $$\psi^{[2a]} = \psi^{[2a]} (\boldsymbol{\overline{C},H}) = \overline{\psi}_{g} (\overline{\boldsymbol{C}}) + \overline{\psi}_{f}^{[2]} (\overline{\boldsymbol{C}},\boldsymbol{H}) + \left. {U(J)} \right|_{\,J = 1}$$

and a *compressible form* $\psi^{[2b]}$:

30 $$\psi^{[2b]} = \psi^{[2b]} (\boldsymbol{\overline{C},H}) = \overline{\psi}_{g} (\overline{\boldsymbol{C}}) + \overline{\psi}_{f}^{[2]} (\overline{\boldsymbol{C}},\boldsymbol{H}) + U(J)$$

where the volumetric strain energy U(J) is defined as:

31 $$U(J) = \frac{1}{4}K\left[ {\left( {J - 1} \right)^{2} + \left( {\log J} \right)^{2} } \right]$$

Finally, the two strain energy functions can be explicitly specified:

32 $$\psi^{[2a]} (\boldsymbol{\overline{C},H}) = \frac{\mu }{2}(\overline{I}_{1} - 3) + \frac{1}{2}a\left\langle {\kappa \overline{I}_{1} + (1 - 3\kappa )\overline{I}_{4} - 1} \right\rangle^{2}$$

33 $$\psi^{[2b]} (\boldsymbol{\overline{C},H}) = \frac{\mu }{2}(\overline{I}_{1} - 3) + \frac{1}{2}a\left\langle {\kappa \overline{I}_{1} + (1 - 3\kappa )\overline{I}_{4} - 1} \right\rangle^{2} + \frac{1}{4}K\left[ {\left( {J - 1} \right)^{2} + \left( {\log J} \right)^{2} } \right]$$

The scalar-valued components of the derivative of $\psi^{[2a]}$ with respect to its invariant arguments are:

34 $$\begin{gathered} \frac{{\partial \psi^{[2a]} }}{{\partial \overline{I}_{1} }} = \frac{{\partial \psi^{[2b]} }}{{\partial \overline{I}_{1} }} = \frac{\mu }{2} + a\,\kappa \left\langle {\kappa \overline{I}_{1} + (1 - 3\kappa )\overline{I}_{4} - 1} \right\rangle \hfill \\ \frac{{\partial \psi^{[2a]} }}{{\partial \overline{I}_{4} }} = \frac{{\partial \psi^{[2b]} }}{{\partial \overline{I}_{4} }} = a\,(1 - 3\kappa )\left\langle {\kappa \overline{I}_{1} + (1 - 3\kappa )\overline{I}_{4} - 1} \right\rangle \hfill \\ \frac{{\partial \psi^{[2a]} }}{\partial J} = \frac{1}{2}K\left( {J - 1 + \frac{\log J}{J}} \right) \hfill \\ \end{gathered}$$

The nominal stress tensor ${\boldsymbol{P}}$ associated with $\overline{\psi }^{[2]}$ is expressed as follows:

35 $${\boldsymbol{P}} = 2{\boldsymbol{F}}.\left( {\frac{{\partial \psi^{[2]} }}{{\partial {\boldsymbol{C}}}}} \right) = 2{\boldsymbol{F}}.\left( {\frac{{\partial \psi^{[2]} }}{{\partial I_{i} }}\frac{{\partial I_{i} }}{{\partial {\boldsymbol{C}}}}} \right) = 2\overline{\user2{F}}.\left( {\frac{{\partial \psi^{[2]} }}{{\partial \overline{I}_{1} }}{\boldsymbol{I}} + \frac{{\partial \psi^{[2]} }}{{\partial \overline{I}_{4} }}{\mathbf{\mathcal{S}}}_{0} } \right) + pJ\overline{\user2{F}}^{{ - {\mathrm{T}}}}$$

where p, the hydrostatic pressure, enters the constitutive equation as a reaction to the kinematic constraint of incompressibility:

36 $${\boldsymbol{P}} = 2\left( {\frac{{\partial \psi^{[2]} }}{{\partial \overline{I}_{1} }}\overline{\user2{F}} + \frac{{\partial \psi^{[2]} }}{{\partial \overline{I}_{4} }}\overline{\user2{F}}.{\mathbf{\mathcal{S}}}_{0} } \right) + pJ\overline{\user2{F}}^{{ - {\mathrm{T}}}}$$

The incompressible version of the nominal stress tensor ${\boldsymbol{P}}$ is given as follows:

37 $${\boldsymbol{P}}_{i} = 2\,\overline{\user2{F}}\left\{ {\left[ {\frac{\mu }{2} + a\,\kappa \left\langle {\kappa \overline{I}_{1} + (1 - 3\kappa )\overline{I}_{4} - 1} \right\rangle } \right]{\boldsymbol{I}} + a\,(1 - 3\kappa )\left\langle {\kappa \overline{I}_{1} + (1 - 3\kappa )\overline{I}_{4} - 1} \right\rangle {\mathbf{\mathcal{S}}}_{0} } \right\} + pJ\overline{\user2{F}}^{{ - {\mathrm{T}}}}$$

while the compressible form of ${\boldsymbol{P}}$ is:

38 $${\boldsymbol{P}}_{c} = 2\,\overline{\user2{F}}\left\{ {\left[ {\frac{\mu }{2} + a\,\kappa \left\langle {\kappa \overline{I}_{1} + (1 - 3\kappa )\overline{I}_{4} - 1} \right\rangle } \right]{\boldsymbol{I}} + a\,(1 - 3\kappa )\left\langle {\kappa \overline{I}_{1} + (1 - 3\kappa )\overline{I}_{4} - 1} \right\rangle {\mathbf{\mathcal{S}}}_{0} } \right\} + K\,J\left( {J - 1 + \frac{\log J}{J}} \right)\overline{\user2{F}}^{{ - {\mathrm{T}}}}$$

## Results

### Determination of the composite mechanical response of the RVE

For each simulated mechanical tests (Sects. 6.2 and 6.3), the nodal forces at each face of the RVE subjected to a displacement boundary condition were recorded and summed at the end of every converged displacement increment to determine the total reaction force experienced by the sample ${\boldsymbol{f}} = \{ f_{x} ,f_{y} ,f_{z} \}$. The surface area of the cross section where nodal forces were measured, ${\mathcal{A}}_{\,0}^{i}$, was determined in the unloaded configuration. ${\mathcal{A}}_{\,0}^{i}$ is the cross section normal to the direction i. The equivalent nominal stress in the direction i was then calculated as $P_{i} = f_{i} /{\mathcal{A}}_{\,0}^{i}$ while the corresponding equivalent Green–Lagrange strain $E_{i}$ was computed as

39 $$E_{i} = \frac{1}{2}\left[ {\left( {1 + \frac{{\Delta u_{i} }}{{{\mathcal{L}}_{\,0}^{\,i} }}} \right)^{2} - 1} \right]$$

where $\Delta u_{i}$ and ${\mathcal{L}}_{\,0}^{\,i}$ are, respectively, the increment of displacement and undeformed length of the sample along the i-th principal direction. The resulting strain–stress is the set of pairs $\{ (E_{i} ,P_{i} ),i = 1..p\}$ where p is the number of displacement increments in the finite element analysis.

### Micromechanical finite element analyses of models 1A, 1B and 1C

In models 1A and 1B, following the standard GOH homogenisation (Gasser, et al. 2006), matrix and fibre contributions are superposed in the continuum, and therefore, at every integration point, there is no geometric separation between the two phases. The fibrous and matrix contributions are smeared throughout the domain, and their relative importance is controlled by the ratio of the material parameters $k_{1} /\mu$ (fibre-to-matrix stress scaling) rather than by a geometric volume fraction. Model 1C is different as every element is classified as either a fibre element or a matrix element based on the Otsu threshold applied to the voxel intensity. Matrix elements have only the neo-Hookean ground-substance energy $\overline{\psi}_{g}$; “fibre” elements have both $\overline{\psi}_{g}$ (isotropic ground substance) and $\overline{\psi}_{f}$ (anisotropic fibre energy). The geometric fibre volume fraction $\nu_{f}^{{{\mathrm{1C}}}} \,$ of model 1C is therefore the ratio of fibre-classified elements to total elements, and takes a distinct value for each FRT.

To be meaningfully comparable models, 1A, 1B, and 1C should have identical fibre volume fractions. This ensures that the volume integral of the fibre strain energy is of comparable order of magnitude across the three models. This could be simply achieved by appropriately scaling the constitutive parameter $k_{1}$. If $\nu_{f}^{{{\mathrm{1C}}}}$ is the fibre volume fraction of model 1C, then the new scaled material parameter $k_{1}$ should be:

40 $$\hat{k}_{1} = \nu_{f}^{{{\mathrm{1C}}}} \,k_{1}$$

**Fig. 21:**
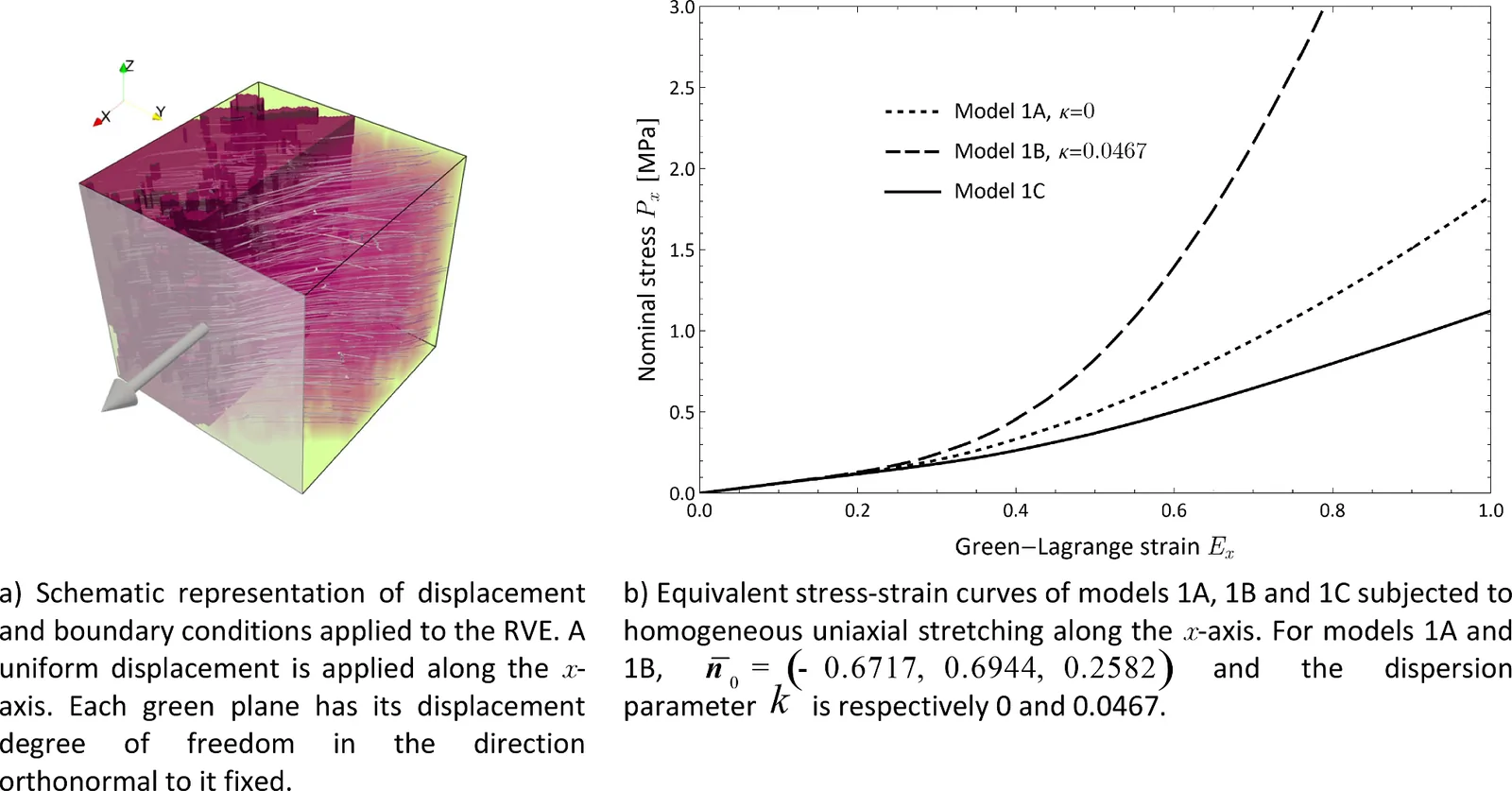
Mechanical response of the RVE for models 1**A** (uniform fibre orientation), 1**B** (uniform fibre orientation with dispersion), and 1**C** (element-based unique fibre orientation) under homogeneous uniaxial extension along the *x*-axis

**Fig. 22:**
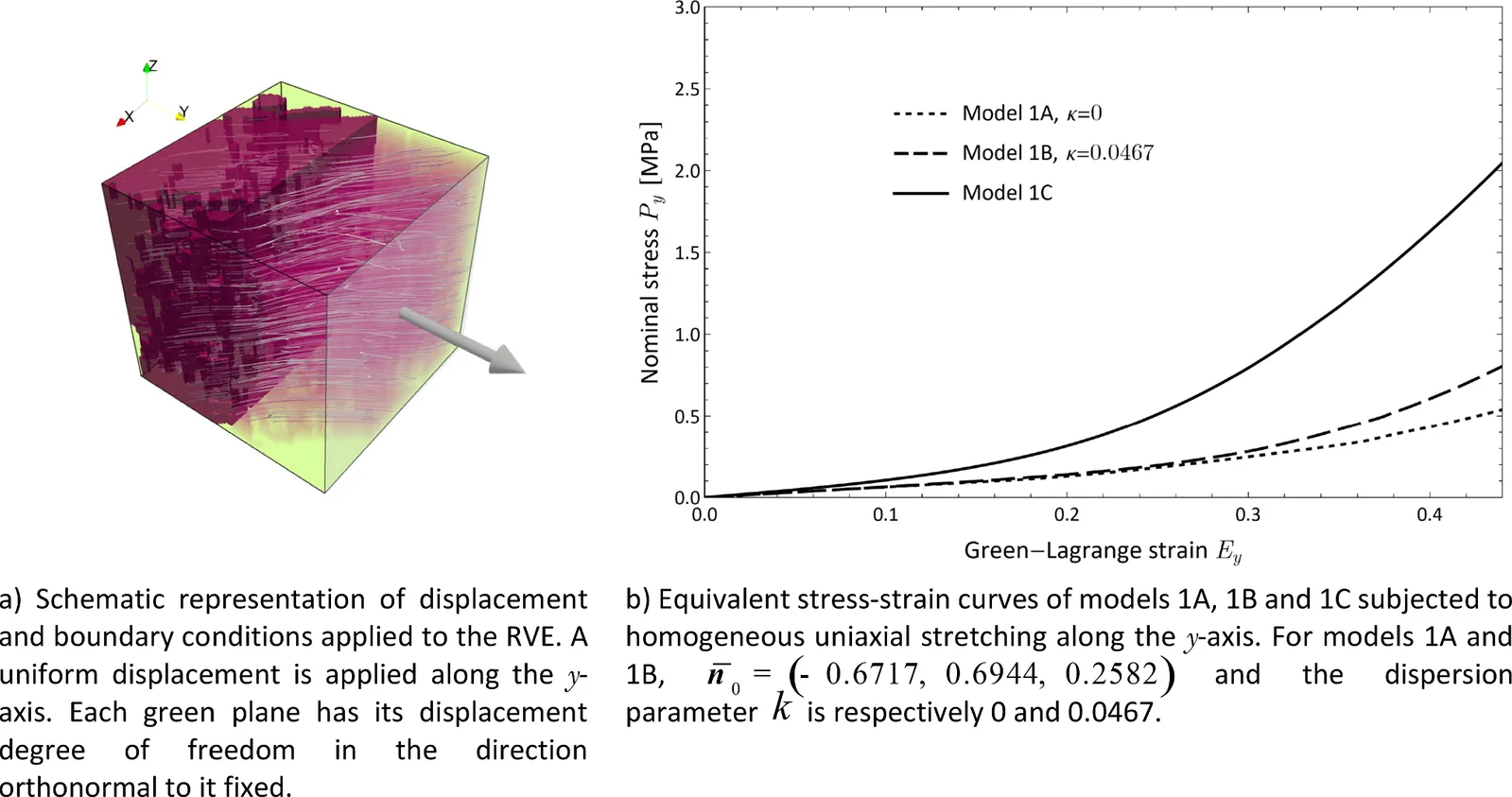
Mechanical response of the RVE for models 1**A** (uniform fibre orientation), 1**B** (uniform fibre orientation with dispersion), and 1**C** (element-based unique fibre orientation) under homogeneous uniaxial extension along the *y*-axis

The computed equivalent strain–stress curves for the RVE of models 1A, 1B, and 1C for uniaxial extension along the *x*-axis, *y*-axis, and *z*-axis are, respectively, shown in Figs. 21, 22**,** and 23. Figure 21 shows that when stretching occurs along the *x*-axis model 1B exhibits the stiffest response with notable strain hardening, followed by model 1A and model 1C. It is striking to note that the inclusion of fibre angle dispersion in model 1B (κ=0.0467) has a significant effect on RVE’s stiffness when compared to that of model 1A. This could be explained by the fact that dispersed fibres may be closely aligned with the direction of load and would therefore start to bear loads at comparatively lower strain level. This observation is consistent with results reported by Gasser et al. (Gasser, et al. 2006). Accounting for local fibre direction in model 1C results in the softer response of all three models, highlighting again how relatively minor local deviations from a mean fibre direction could alter the overall mechanical response of the RVE.

**Fig. 23:**
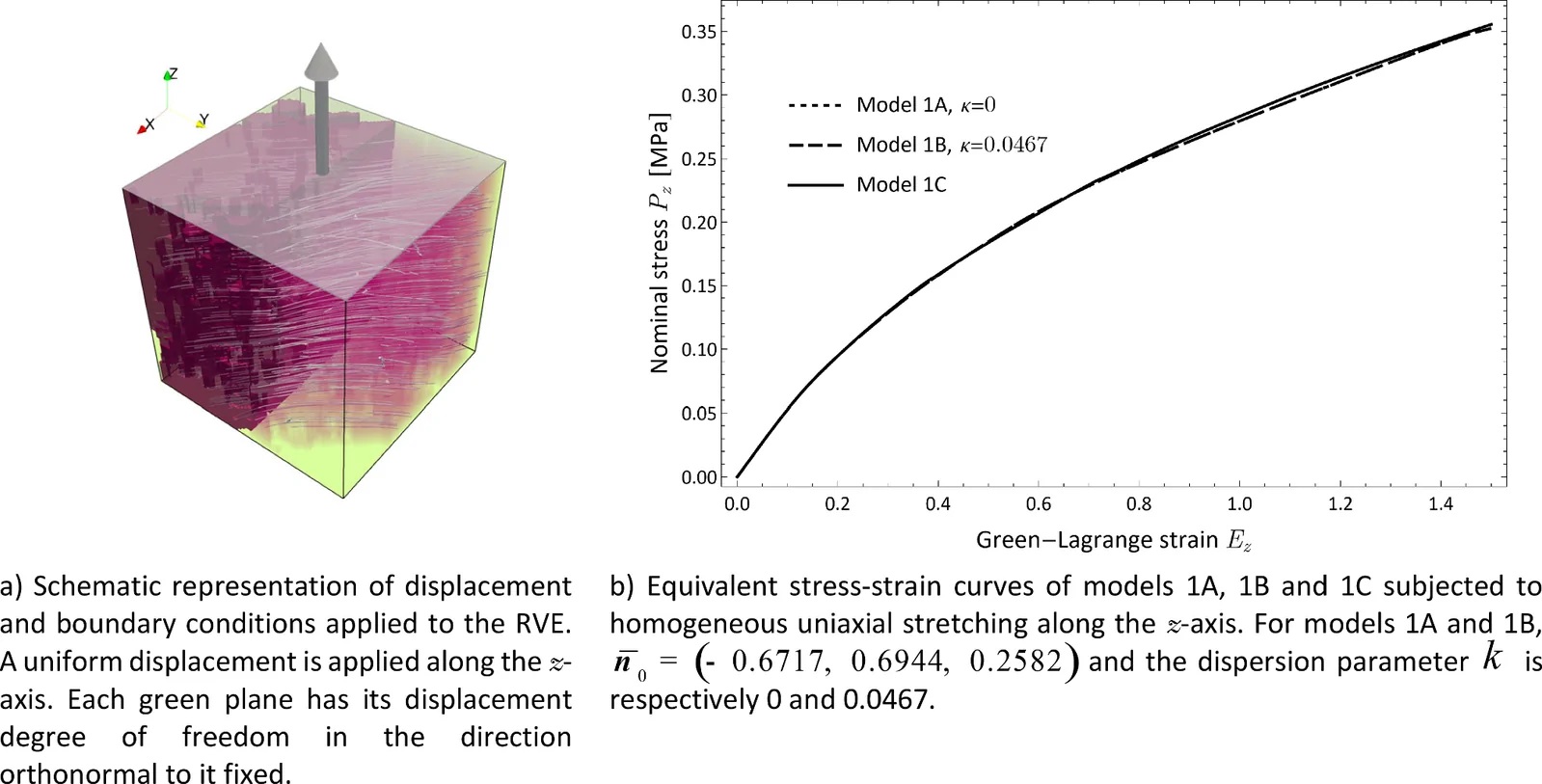
Mechanical response of the RVE for models 1**A** (uniform fibre orientation), 1**B** (uniform fibre orientation with dispersion), and 1**C** (element-based unique fibre orientation) under homogeneous uniaxial extension along the *z*-axis

The trends observed in Fig. 21 in terms of the stiffest response are reversed for the case of a uniaxial extension along the *y*-axis (Fig. 22). In this case, the stiffest response is obtained for model 1C, followed by model 1B and 1A. Moreover, at equivalent Green–Lagrange strain (e.g. 0.44) model 1C experiences a significantly higher equivalent uniaxial stress along the *y*-axis (2.1 MPa) than models 1A and 1B do (respectively, 0.5 and 1 MPa). These differences are closely related to the underlying microstructural characteristics of the collagen fibre network in relation to loading orientation. Fibres in both models 1A and 1B are uniformly oriented at an about 44° azimuthal angle with respect to the *y*-axis while model 1B additionally features a degree of fibre dispersion around this mean fibre orientation. This implies that for a given deformation applied to the RVE, collagen fibres in model 1B are likely to bear load at a lower equivalent RVE strain level, and thus experience equivalent stress levels with minimum fibre rotation. Fibres in model 1C are likely to align non-uniformly with the loading direction. As a result, model 1C requires a lesser extension to generate the same equivalent stress, suggesting a stiffer global mechanical response under the same global (i.e. at the RVE level) deformation conditions. There are no significant differences for the equivalent stress–strain curves of models 1A, 1B, and 1C when subjected to stretch along the *z*-axis (Fig. 23). This can be attributed to the mostly in-plane fibre orientation distribution (i.e. *xy*-plane). As shown in Fig. 13, the preferred mean material direction in models 1A and 1B forms an approximately 75° inclination angle with the *z*-axis. Despite each fibre element having its own orientation, most fibres in model 1C are also nearly parallel to the *xy*-plane. The mechanical response of these models is influenced not only by their structural orientation but also by the loading direction. Since the stretching direction is roughly perpendicular to the mean fibre orientation in all three models, the fibres are, on average, not recruited, and therefore do not bear any load.

**Fig. 24:**
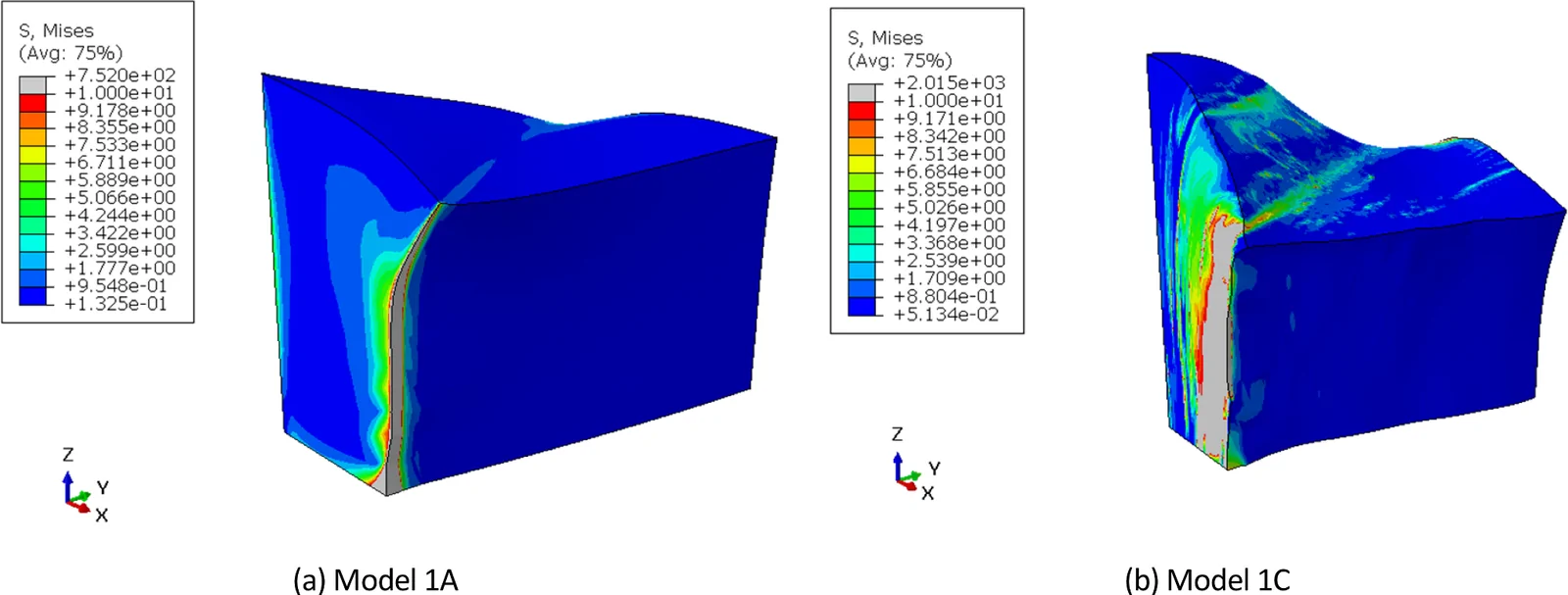
Von Mises stress distribution for model 1A and model 1C at approximately 44.6% Green–Lagrange strain along the *y*-direction. The maximum stress value on the colour scale was set to 10 MPa for both models

**Fig. 25:**
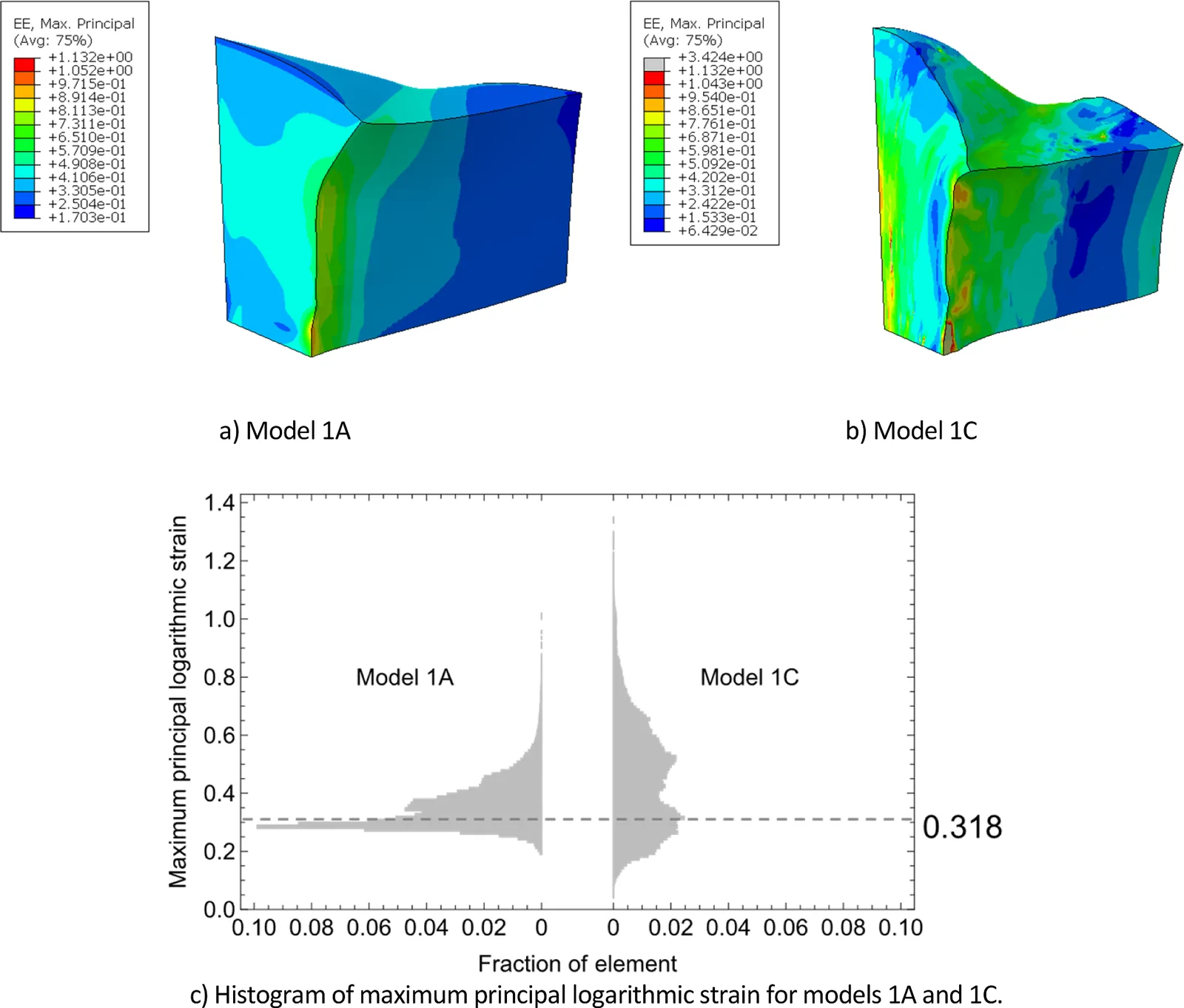
Colour plot of maximum principal strains for** a**) model 1A and** b**) model 1C, and** c**): corresponding histograms, at approximately 44.6% equivalent Green–Lagrange strain along the *y*-direction (i.e. RVE’s stretch of 1.375 and $\ln (1.375) \simeq 0.318$. The maximum strain value on the colour scale was set to 1.13

The load is mainly born by the isotropic matrix resulting in similar mechanical responses across the three models (Fig. 23). This finding is similar to the stretch response of models incorporating isotropic orientation distribution, as reported by Gasser et al. (Gasser, et al. 2006) who found that the response is independent of mean fibre orientation. As intuitively expected, despite the application of pseudo-homogeneous boundary conditions, the presence of oriented fibres not aligned with loading directions results in non-homogeneous deformation patterns and stress distribution. In addition to the effect of global/local fibre orientation, the assumption of full or near incompressibility also influences the model’s deformation. The von Mises stress distribution (Fig. 24) and the maximum principal strains (Fig. 25**-c**) observed in models 1A and 1C further illustrate this finding.

**Fig. 26:**
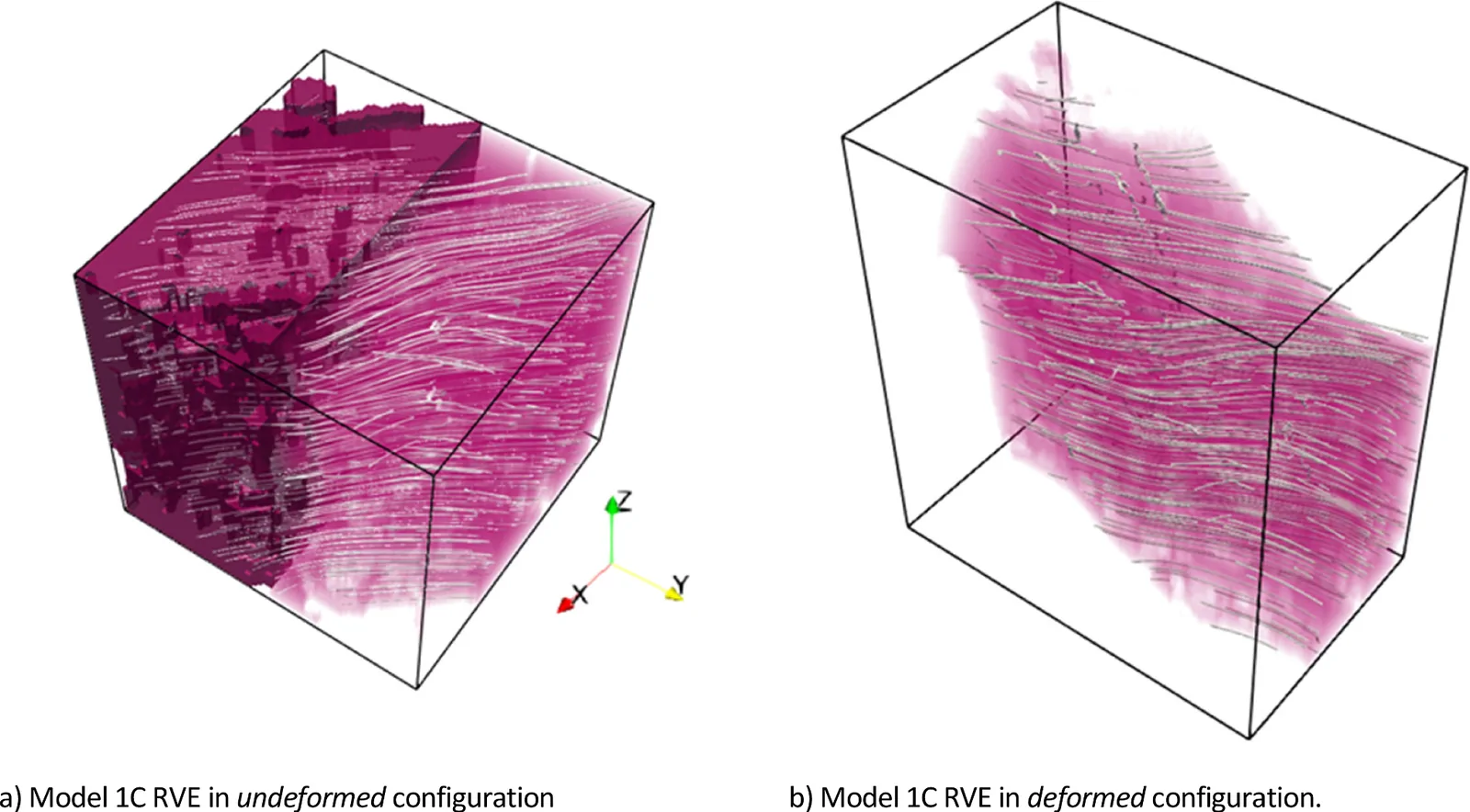
Visualisation of fibre orientation in model 1C RVE in the undeformed and deformed configurations after application of pseudo-homogeneous uniaxial tension along the *y*-axis. The corresponding equivalent Green–Lagrange strain was about 44.6%. Streamlines of the fibre vector field were traced in ParaView through the use of the Stream Tracer filter. Mulberry colours indicate the fibre phase of the RVE delimited by a black outline

Under the imposed RVE logarithmic strain of 0.318 along the *y*-axis, both models 1A and 1C exhibit the same peak logarithmic strain value of around 0.3 (Fig. 25**-c**). However, model 1C features a second peak at around 0.5 and, overall, exhibits a broader strain distribution than that of models 1A (and 1B). In models 1A and 1B, the fibre phase is uniformly distributed in the RVE volume whereas in model 1C, there are two explicitly distinct fibre and matrix phases (Fig. 26). Consequently, for a given RVE’s stretching, the soft isotropic matrix elements in model 1C experience larger local strains than the same element featuring a continuum implicitly made of matrix and stiffer fibre phase in models 1A and 1B.

**Fig. 27:**
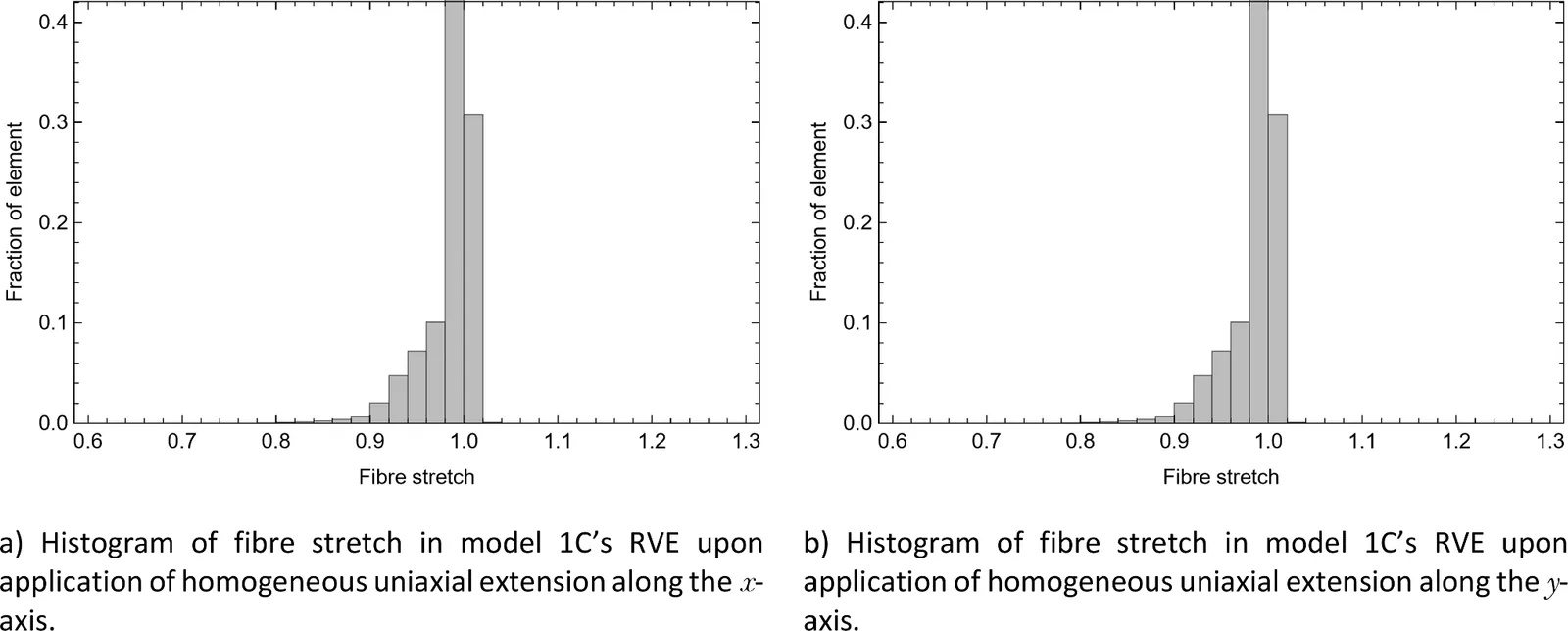
Normalised histograms of fibre stretch in model 1C’s RVE after application of load corresponding to a 44.6% Green–Lagrange strain. Bin width was set to 0.02 for both analyses

Figure 26 illustrates the deformation of model 1C and its underlying collagen fibre structures by showing its *undeformed* and *deformed* configuration after application of pseudo-homogeneous uniaxial tension along the *y*-axis. However, according to the histogram of fibre stretch distribution shown in Fig. 27-b, not all fibres were subjected extension along their respective axes. When the equivalent Green–Lagrange strain was around 44.6% along the *y*-axis, only about 50% of the fibres were stretched. This finding can be attributed to the non-uniform fibre orientation distribution and the loading direction not perfectly aligned with multiple fibre orientations. The equivalent Green–Lagrange strain $E_{i}$ of the RVE is calculated based on the displacement and measures the material’s relative deformation in terms of change of length only. When the fibre direction is not aligned with the loading direction, the corresponding fibre stretch value will not be the same as the value defined as $\lambda_{\,i} = \sqrt {2E_{i} + 1}$ which is derived from the Eq. (40). This can explain why some fibre stretch values are less than the 1.375 stretch value obtained upon application of load corresponding to a 44.6% Green–Lagrange strain along *y*-axis.

It is relevant to point out that models 1A and 1B distribute the fibre energy homogeneously whereas model 1C distributes it heterogeneously. Even after matching the integrated fibre stiffness (see Sect. 7.2), the spatially heterogeneous distribution in model 1C leads to multifold differences in RVE-level stress response.

### Influence of interpolation method on RVE’s micromechanics

The details of this analysis which aimed at comparing the effects of fibre vector field interpolation methods on the micromechanics of the RVE were described in Sects. 4.4 and 6.3**.**

#### Full-resolution mesh—Model 2A

**Fig. 28:**
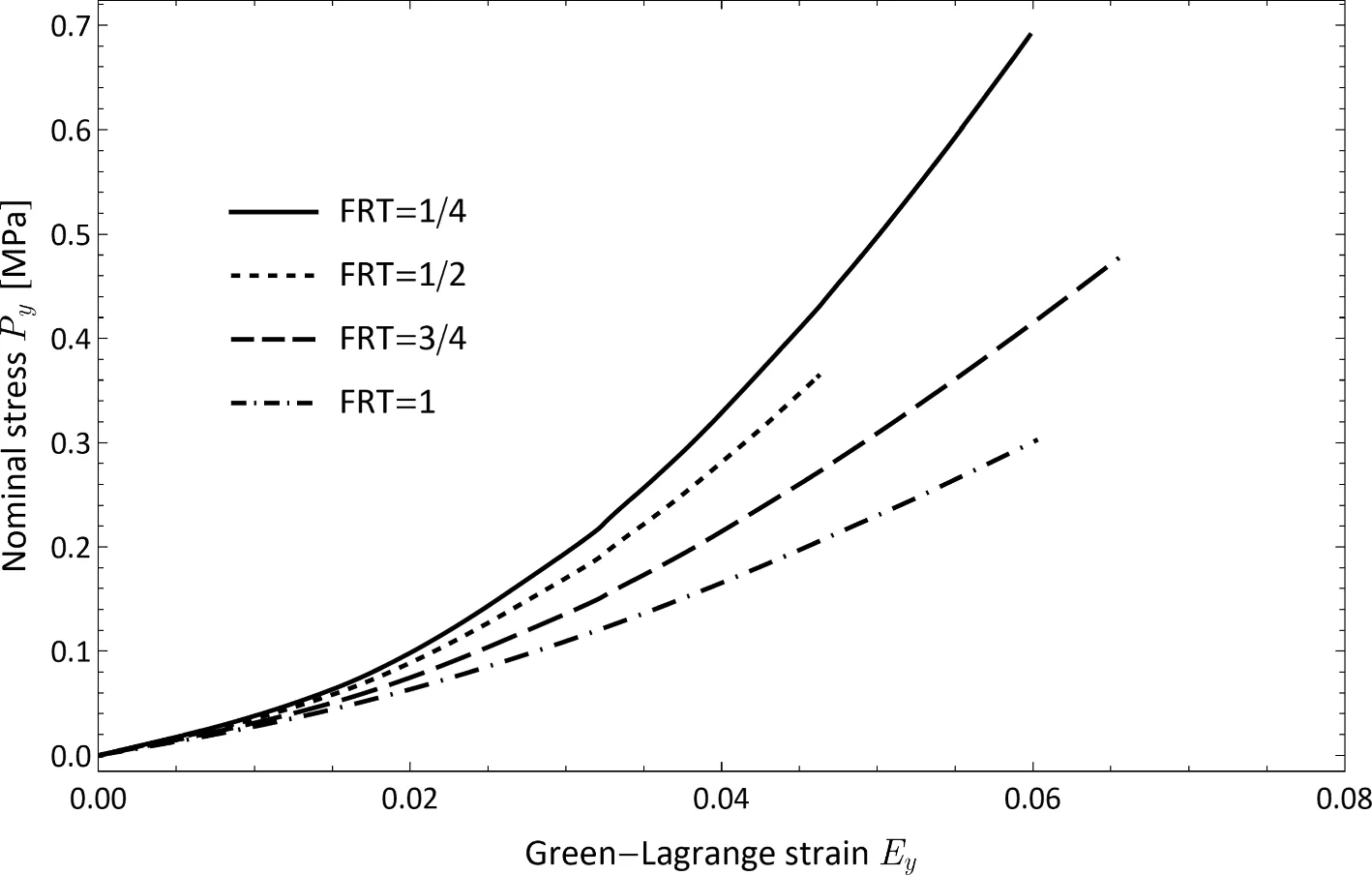
Equivalent nominal stress-Green–Lagrange strain curves of the full-resolution mesh (model 2A) with applied uniaxial tensile boundary condition along the *y*-direction. FRT denotes fibre ratio threshold of model 2A that employed Ní Annaidh et al*.*’s constitutive parameters (Annaidh, et al. 2012)

Model 2A was subjected to a pseudo-homogeneous uniaxial tension along the *y*-axis, and the corresponding equivalent nominal stress-Green–Lagrange strain curves were collected for each of the four possible fibre ratio thresholds (FRT): 1/4, 2/4, 3/4 and 4/4 (Fig. 28). FRT conditions how much volume of the RVE is occupied by elements featuring a fibre strain energy contribution (i.e. fibre volume fraction). The lower the FRT the larger the fibre volume fraction. Therefore, it is expected that a lower FRT would result in a stiffer RVE’s response as shown in Fig. 28. FRT is therefore a critical parameter of the fibre assignment algorithm.

**Fig. 29:**
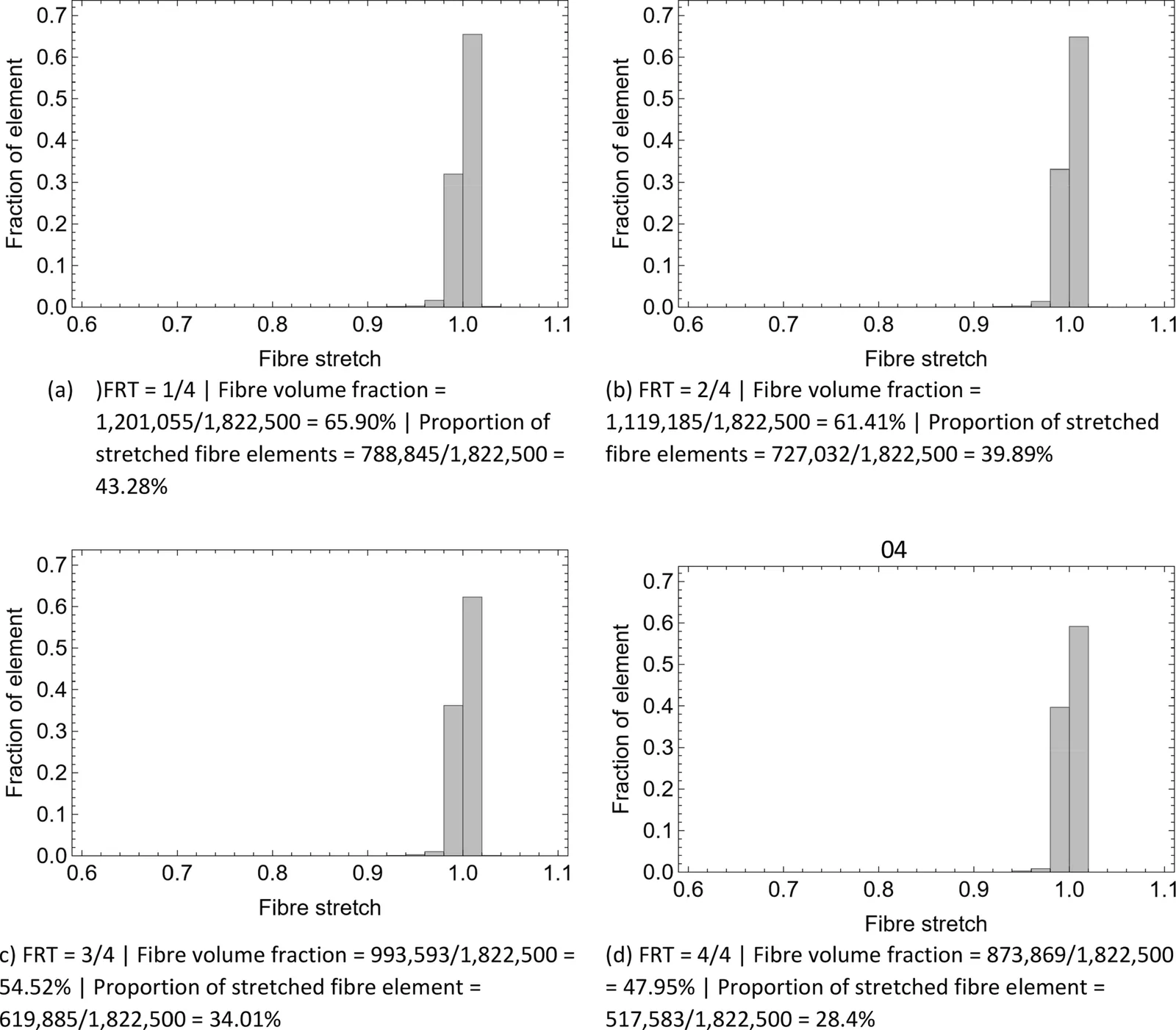
Histograms of fibre stretches for model 1A for the four possible values of FRT. The corresponding Green–Lagrange strain is 0.03 for all analyses. The histogram bin width was set to 0.02

The contribution of fibres to the overall equivalent mechanical response can be inferred from the histogram of fibre stretches shown in (Fig. 29). Here, a parameter, called “proportion of stretched fibre elements”, is introduced. This parameter is defined as the ratio of the total number of stretched fibre elements (i.e. with a stretch along the local fibre axis value exceeding 1 to the total number of all elements in the model (i.e. 1,822,500 for Model 2A)). The proportion of stretched fibre elements was 43.28%, 39.89%, 34.01%, and 28.4% for the following respective FRT values: 1/4, 1/2, 3/4, and 1, thereby providing additional quantitative insight to the results shown in Fig. 28.

It is generally accepted that in biological soft tissues most collagen fibres remain crimped and intertwined during the very early stage of deformation (normally considered when the stretch is below 1.1), contributing minimally to the tissue’s stiffness (Kalra, et al. 2016). Additionally, at this stage, the initial slope measured from the stress–strain curve of the overall tissue (i.e. tangent stiffness) is considered to be mainly the stiffness of the isotropic part of the extracellular matrix (i.e. ground substance). Figure 28 shows that the initial slope of the equivalent stress–strain curve was mildly influenced by the fibre volume fraction of the RVE. Our models featuring element-wise or integration point-wise local fibre directions lead to more heterogeneous along-the-fibre stretch distributions when compared to the equivalent distributions arising in classical continuum fibre-reinforced models featuring uniformly distributed fibre directions with or without dispersion (Gasser, et al. 2006).

#### Vector-downsampled mesh—model 2B

**Fig. 30:**
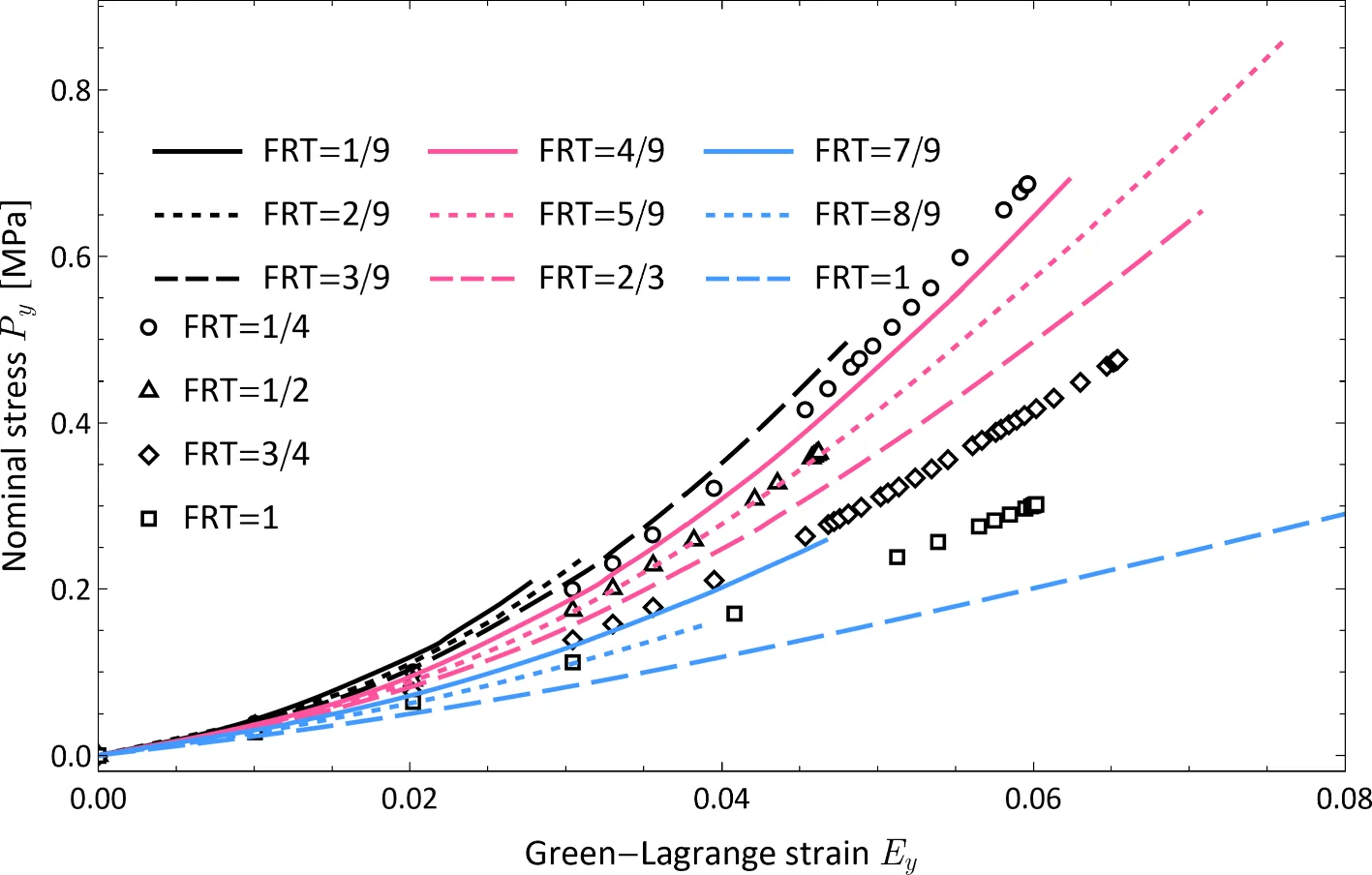
Equivalent stress–strain curves of the vector-downsampled model 2B with an applied uniaxial tensile boundary condition along *y*-direction. VD_FRT represents fibre ratio threshold of the vector-downsampled FE mesh that employed Ní Annaidh et al*.*’s constitutive parameters (Annaidh, et al. 2012). The stress–strain relationship of full-resolution FE mesh (model 2A) is also plotted in scatter for comparison

Model 2B was subjected to the same pseudo-homogeneous uniaxial tension along the *y*-axis as was model 2A. The corresponding equivalent nominal stress-Green–Lagrange strain curves were collected for each of the nine possible FRT: 1/9, 2/9, 1/3, 4/9, 5/9, 2/3, 7/9, 8/9, and 1, and plotted against the responses of model 2A (Fig. 30). The general trend for the mechanical response of model 2B to FRT exhibits a similar trend to that of model 2A. The FRT conditions the integration of fibres and their volume fraction into the downsampled finite element mesh. A lower FRT of the vector-downsampled mesh (VD_FRT) leads to a higher fibre volume fraction, resulting in more fibres bearing load under uniaxial stretching along the *y*-axis. For a Green–Lagrange strain lower than 3%, the mechanical response corresponding to VD_FRT of 1/9 is the stiffest while that corresponding to VD_FRT of 9/9 is the softest.

As the full-resolution mesh/model, model 2A was considered the reference against which the accuracy of model 2B and associated VD_FRT was evaluated. In terms of stress–strain curve response of the full-resolution and vector-downsampled RVE, the sets (FRT = 2/4;VD_FRT = 5/9) and (FRT = 4/4;VD_FRT = 8/9) had a closely matching response, respectively, with 4.8 and 6.2% relative difference while the other two sets including (FRT = 1/4;VD_FRT = 4/9) and (FRT = 3/4;VD_FRT = 7/9) exhibited a larger error: respectively, 19.4 and 16.8% relative difference.

The aim of downsampling is to reduce model size in order to decrease computing expenses. However, it is also essential to maintain accuracy of the computed micromechanical response of the RVE and assess the impact of downsampling on this. Computing times for models 2A and 2B and their associated FRTs, VD_FRTs, and reached strain levels are listed in Table 4.

**Table 4:** Comparison of computational costs for model 2A and 2B with different fibre ratio thresholds in terms of used RAM and running time

Model name	Cores/Job used RAM	Green–Lagrange strain level	Computing time
Model 2A_FRT = 1/4	120/ 185.83 GB	0.06	09:51:22
Model 2A_FRT = 2/4	120/ 188.29 GB	0.046	09:31:20
Model 2A_FRT = 3/4	120/ 195.33 GB	0.065	13:22:47
Model 2A_FRT = 4/4	120/ 184.82 GB	0.060	10:53:05
Model 2B_VD_FRT = 1/9	120/ 43.54 GB	0.028	01:28:53
Model 2B_VD_FRT = 2/9	120/ 42.78 GB	0.031	01:55:49
Model 2B_VD_FRT = 3/9	120/ 43.33 GB	0.048	01:42:16
Model 2B_VD_FRT = 4/9	120/ 44.26 GB	0.062	02:22:31
Model 2B_VD_FRT = 5/9	120/ 42.90 GB	0.076	01:29:00
Model 2B_VD_FRT = 6/9	120/ 43.56 GB	0.071	01:57:17
Model 2B_VD_FRT = 7/9	120/ 43.80 GB	0.047	02:16:40
Model 2B_VD_FRT = 8/9	120/ 43.65 GB	0.039	01:25:40
Model 2B_VD_FRT = 9/9	120/ 43.45 GB	0.082	01:42:02

When employing 120 computing cores to run the analyses for models 2A and 2B, with each core assigned 4 GB RAM, the solving time of the vector-downsampled model (i.e. model 2B) was significantly lower than that of full-resolution model (model 2A). Additionally, the convergence properties of the nonlinear finite procedures for models 2A and 2B are also different. For the set (FRT = 2/4;VD_FRT = 5/9), model 2B performed better than model 2A in terms of convergence. For the set (FRT = 3/4;VD_FRT = 7/9), the convergence performance of the downsampled model was not as good as that of the full-resolution model. This indicates that fibre orientations in model 2B for VD_FRT = 5/9 may be smoother than for other VD_FRTs. Severe discontinuities in the fibre vector field are compounded by the exponential mechanical response of fibres and the discontinuous non-smooth tension–compression switch in the strain energy function (Eq. (22)).

### The role of explicit fibre/fibre bundle unfolding on RVE mechanics

**Fig. 31:**
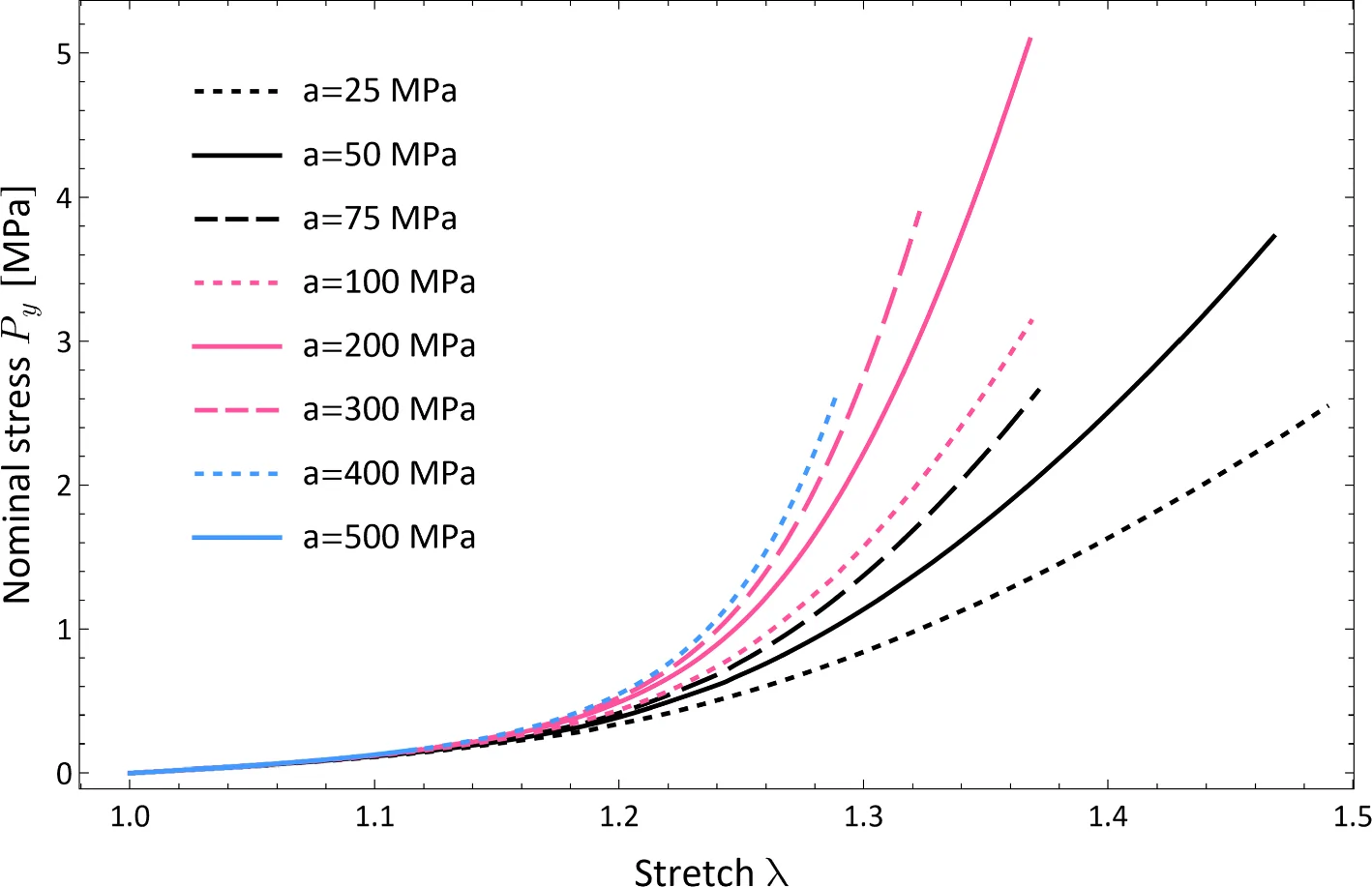
Equivalent stretch-nominal stress curves of model 1C subjected to homogeneous uniaxial stretching along the *y*-axis as a function of the elastic modulus of collagen fibres a

The results of the FE analyses of model 1C described in Sect. 6.4 are presented as stretch-nominal stress curves given as a function of the elastic modulus of collagen fibres a (Fig. 31). Although the strain energy function associated with collagen fibre elasticity is quadratic (Eq. (27)—and therefore the corresponding stress function is linear—Fig. 31 clearly shows that the mechanical response of the RVE features the typical exponential behaviour observed for biological tissues. Strain hardening monotonically increases with the modulus of elasticity of collagen fibres, a. This clearly highlights the fundamental role of fibre orientation when considered at the local level within an RVE rather than when considered spatially uniform within an RVE. Our voxel-based FE analyses demonstrates that the exponential stress response of the RVE stems from structural mechanism associated with local fibre deformation rather than through the nonlinear *material* behaviour of individual fibres or fibre bundles. This result is consistent with the long-established view that the J-shaped stress–strain response of collagenous tissues arises primarily from progressive fibre recruitment and reorientation (Bancelin, et al. 2015, Diamant, et al. 1972, Fung 1981, Morin, et al., 2021, Pissarenko and Meyers 2019, Wahlsten, et al. 2023). Our voxel-based computational analyses with a purely quadratic fibre energy reproduce the characteristic exponential-like stiffening. This quantitatively confirms, at the voxel level and for image-based RVEs of human dermis, that the macroscopic nonlinearity can be recovered without invoking a nonlinear material law at the fibre scale.

## Discussion

In this study, motivated by the quest for enabling a better mechanistic understanding of the micromechanics of the dermis, a fundamental component of the skin, we developed an integrated methodology pipeline from high-resolution 3D imaging modalities capturing the intricate architecture of collagen fibres to large-scale nonlinear finite element analyses of RVE of biological samples. To extract local fibre orientation at voxel level, we employed a 3D structure tensor eigenanalysis which proved robust and fast on SBF-SEM images using minimal image pre-processing and the open-source code developed by Jeppesen and colleagues (Jeppesen, et al. 2021). The workflow further integrates automatic generation of ready-to-run Abaqus/Standard® FE analysis input files to be used in concert with a custom-made library of user material subroutines UMAT that code various invariant-based transversely isotropic hyperelastic constitutive models. Ultimately, various voxel-based FE models were built and analysed to gain novel insight into the mechanical effects on tissue ROIs of capturing local fibre orientation at voxel/integration point level in contrast to approaches assuming a spatially uniform orientation distribution (i.e. constant in the ROI/RVE) like for most models found in the literature.

The key take-away message from models 1A, 1B, and 1C (Sect. 7.1) is that capturing fibre and matrix volume fractions together with local fibre orientation is critical in modulating the mechanical response of a RVE. Comparing the FE analysis results of these three models demonstrates that accounting for heterogeneous fibre orientations within the RVE could lead to multifold increase in the stress response. Of course, this result must be considered in the light of the particular strain energy density used representing the elastic response of fibres and its relation to the microstructural mechanisms responsible for the observed macroscopic stress exponential behaviour (see Sect. 6.4). Both models 1A and 1B feature a spatially uniform fibre orientation distribution but the relatively low fibre dispersion of model 1B is sufficient to lead to a significantly different response (κ=0.0467) to that of model 1A depending on misalignment of mean fibre vector and load direction. The analytical form of fibre dispersion integration, whether through angular integration (Lanir 2017) or structure tensor approaches (Gasser, et al. 2006), offers a convenient way to capture tissue anisotropy in an averaged sense. However, these continuum formulations fundamentally rely on homogenisation assumptions that preclude accurate prediction of fibre-level strain distributions and long-distance force transmission along individual fibres (He, et al. 2024). The affine deformation assumption which implies that fibre stretch relates directly to macroscopic strain through structure tensors breaks down when fibre reorientation, fibre–fibre interactions, or matrix constraint effects become significant. He et al. (He, et al. 2024) demonstrated that even when continuum models match global displacement responses, they may fail to capture local strain patterns and fibre-level mechanics critical for understanding mechanobiological phenomena. This observation is also corroborated by our numerical results. Direct fibre models overcome these limitations by explicitly representing fibre geometry, enabling accurate prediction of both tissue-level strain heterogeneity and fibre-level strain transmission (He, et al. 2024) despite limitations in accurately capturing these features. The approach naturally captures non-affine deformations, fibre–fibre interactions mediated through the surrounding matrix, and long-range mechanical force transmission along continuous fibre paths. However, the computational expense increases substantially with the number of explicitly modelled fibres, limiting application to relatively small tissue domains or requiring high-performance computing resources.

Although our voxel-level fibre orientation assignment approach uses a spatially heterogeneous fibre vector field (models 1C, 2A-B) within a RVE, the deformation within each fibre element is still affine, i.e. the fibre stretch is computed directly from the local deformation gradient (see paragraph following Eq. (15)), which is the standard affine-kinematic assumption (Chandran and Barocas 2006; Jayyosi, et al. 2017). We retain locally-affine element-wise kinematics with global non-affinity emerging from heterogeneity, rather than fully non-affine discrete-fibre kinematics. The implications are multifold. Fibre–matrix force transfer is enforced kinematically at the element level which means that no sliding, delamination, or fibre–fibre interaction via the matrix is resolved. Long-range fibre tension transmission along a continuous fibre trajectory is only captured as far as contiguous fibre-classified elements form a percolating path of aligned vectors.

Where the element-level vector field is noisy or discontinuous, continuity of fibre tension is lost. (This is the well-known non-affine "strain transmission" effect identified by He et al. (He, et al. 2024) and by Chandran & Barocas (Chandran and Barocas 2006).). Poisson effects in our model are driven by the neo-Hookean matrix and the anisotropic fibre contribution, not by the geometric "volume-densification by bending/buckling" mechanism observed in discrete-fibre networks (Morin, et al., 2021; Vader, et al. 2009). Our model is therefore expected to underestimate the large Poisson's ratios reported at high stretch in soft tissues (Dwivedi, et al. 2022).

Another notable output of our study is that, through image-based micromechanical FE analyses, we confirmed how the typical “J-curve” of collagenous soft tissues can arise purely from microstructural effects associated with the progressive deformation of the local architecture of collagen fibres/bundles upon application of boundary and loading conditions to the RVE. This observation supports the consensual view that the progressive recruitment and unfolding of collagen fibres is the key mechanism behind the strain-hardening effect (Bancelin, et al. 2015, Diamant, et al. 1972, Fung 1981, Morin, et al., 2021, Pissarenko and Meyers 2019, Wahlsten, et al. 2023). It is important to reiterate that the results presented in Sect. 7.4 were obtained using a quadratic function for the fibre strain energy, thus leading to a linear stress function of the first deviatoric invariant of the Cauchy–Green deformation tensor.

The approach proposed in this study is a rational compromise between biophysical accuracy and technological tractability. Exact fibre geometries (e.g. diameter and length of individual fibres) and fibre network characteristics (e.g. interconnections and fusion of fibres) are not captured due to current limitations in image acquisition techniques and image-processing algorithms. However, the averaged voxel-level local fibre orientation and extracted fibre phases are accounted for in our methodology, leading to new insights into the micromechanics of the dermis owing to a better representation of local anisotropy. As mentioned earlier, the mechanical response of RVEs is highly dependent on whether spatially uniform or spatially heterogeneous fibre orientation (i.e. local fibre orientation) distributions are used with multifold variations (see Sect. 7.1). This calls into questions the use of spatially uniform fibre orientation distributions in FE models of collagenous-rich soft tissues aiming at gaining a mechanistic insight into the micromechanics associated with fibre-level mechanobiological phenomena such as mechanotransduction (Di, et al. 2023). The novel approach offers a new level of biofidelity compared to the usual single orientation distribution per family of fibres adopted when modelling collagenous tissues with continuum fibre-reinforced models (Alastrué, et al. 2010; Gasser, et al. 2006).

Our finer approach in terms of capturing local collagen fibre orientation provides a quantitative framework to test the hypothesis that spatially heterogeneous fibre orientation modulates the local mechanical environment sensed by dermal fibroblasts. Fibroblast mechanosensing is driven by local stress and strain at the cell scale (Ayad, et al. 2019; Hinz 2016; Tomasek, et al. 2002). Model 1C exhibits markedly broader and more heterogeneous strain distributions (Fig. 26**-c**) than for the equivalent models 1A / 1B with spatially uniform fibre orientation distributions, potentially suggesting that the classical homogenised GOH model may systematically underestimate the cellular-scale strain heterogeneity to which fibroblasts are exposed.

Our framework could also serve as the microscale input to a future multiscale mechanobiological model in which fibroblast activity is coupled to the local collagen architecture, as has been proposed for wound healing (Buganza Tepole and Kuhl 2016; Sohutskay, et al. 2021). Additionally, it provides image-based statistical descriptors of the collagen architecture (orientation histograms, concentration parameters) that could be used as input to structure tensor-based mechanobiological models (Cyron and Humphrey 2017) or correlated with age, disease, or treatment states in a longitudinal setting. Alterations in the structural and biochemical organisation of collagen fibres can reveal underlying physiological and pathological processes (Wang, et al. 2023). For example, changes within the extracellular matrix such as increase in collagen content and fibre alignment have been shown to be correlated with myocardial infarcted tissues (Quinn, et al. 2016). In skin, ageing is also marked by significant evolution of collagen density and orientation (Limbert, et al. 2019; Pailler-Mattei, et al. 2013). The first part of our methodology (i.e. image acquisition, processing and analysis) could simply be used to analyse 3D collagen organisation in a wide range of biological soft tissues by calculating structural metrics that could reflect particular normal or pathological states.

The framework could prove valuable in longitudinal animal studies where skin samples could be taken at various time points to study the effects of extrinsic ageing factors (e.g. UV radiations) and the subsequent effects of mitigating treatments (Fisher, et al. 1996; Marcos-Garcés, et al. 2014). It is important to emphasise that, in its presented form, our modelling approach does not account for interstitial fluid phase and there are no explicit cells that is fibroblasts and keratinocytes are not resolved, and strains are computed in an acellular matrix.

Although the proposed methodology offers the ability to account for local spatial collagen fibre structural characteristics through high-resolution imaging techniques and integrate that information into a robust FE modelling workflow, the resulting FE meshes would contain millions of elements, which is a significant challenge for nonlinear solving procedures in terms of computational cost. For example, analysing model 2 (see Sect. 6.3) with a fibre ratio threshold of 3/4 on the University of Southampton Iridis 5 supercomputer using 120 cores and 474 GB RAM required up to 13 h of running time. Therefore, it is necessary to consider methods to reduce mesh size (e.g. image or vector field downsampling) while preserving accurate microstructural information. From conducting multiple tests (not included in this study), it was established that our method to resample the full-resolution mesh/fibre vector field from full-resolution images (see Sect. 4.4) appears to be superior to the approach which consists of firstly downsampling images and then applying the fibre extraction algorithm and voxel-based mesh generation. The part of the resampling algorithm to decide whether a voxel is associated with fibre or matrix phase is based on FRT which has been shown to have a significant influence on the mechanical response of the RVE. In principle, the fibre vector field downsampling method can be applied multiple times to reduce mesh size by 4 at each pass.

SB-FSEM imaging inherently limits the accuracy of voxel-based models. This approach offers high-resolution in the image plane (i.e. 8 nm in the *x*–*y* plane) but is restricted by the *z*-distance between two consecutive images. In our study, we used a microtome which can achieve 50 nm slice thickness while a 25 nm minimum thickness can be achieved utilising current commercial microtomes. The mean diameters of collagen fibres in the dermis were found to be 56.2 ± 2.5 nm (papillary dermis) and 62.8 ± 4.3 nm (reticular dermis) (Barton and Marks 1984). In a multiage group (20 s, 40 s, 60 s) study using second-harmonic-generation imaging of reticular dermal collagen in human facial cheek, Ogura et al. (Ogura, et al. 2019) reported values of 134.7 ± 4.1 μm, 126.9 ± 2.2 μm, and 124.7 ± 4.7 μm for subjects, respectively, in their 20 s, 40 s, and 60 s. In the light of these data, our anisotropic voxel-based FE models relying on a 50 nm image “thickness” seem to be adequate for capturing the collagen network in skin dermis with sufficient accuracy. While offering a much lower optical resolving power than that of SBF-SEM, µCT imaging provides the advantage of isotropic voxels by design.

Chemical fixation, heavy-metal staining, dehydration, and resin embedding required for SBF-SEM imaging all perturb the native state of soft tissues. Typical tissue shrinkage reported in the literature for glutaraldehyde-fixed, ethanol-dehydrated, resin-embedded tissue is in the range 4–17% linearly, with the largest contributions arising from ethanol dehydration rather than from chemical fixation itself; Goggin et al. (Goggin, et al. 2020) provide a detailed discussion of these effects and of strategies for their mitigation in the SBF-SEM context but for bone. In skin specifically, the dermis contains approximately 60–70% water by wet weight (Wiig and Swartz 2012), so resin replacement of the aqueous phase necessarily alters the relative packing and apparent volume fraction of the fibrous phase. This aspect of sample preparation has consequences on our modelling approach. Shrinkage is approximately isotropic at the sub-bundle scale, so the local fibre vector field which is what our method extracts should be representative of the in vivo orientation distributions. This is consistent with recent correlative light–electron microscopy studies in skin (Starborg, et al. 2013) and aorta (Niestrawska, et al. 2016) that report preserved angular distributions after rOTO processing. The fibre pixel ratio threshold (Sect. 4) is sensitive to this bias, and this is why we treat the fibre pixel ratio as a parameter and performed/reported a sensitivity analysis in Sects. 6.3 / 7.3, rather than taking a single fixed value as the in vivo truth. Interstitial fluid effects are by construction absent in the ex vivo resin-embedded state. Our RVE analyses are therefore solid-phase only. Comparison of our computed stress–strain responses with in vivo or fully hydrated ex vivo data would require the use of a poroelastic/biphasic extension, as recently proposed by Sachs et al. (Sachs, et al. 2024) in their quadriphasic model of the dermis and by (Dwivedi, et al. 2022) for skin stress relaxation.

Accurate and reliable characterisation of macroscopic tissue/building blocks properties across multiple length scale remains an extremely challenging endeavour (Pissarenko and Meyers 2019; Wahlsten, et al. 2023) because of inherent technological limits of what material/structural features/length scale can be practically characterised experimentally, significant intra- and inter-individual biophysical variability, and because of modelling assumptions that must be made for conducting material identification procedures whether they rely on analytical or computation models (Ní Annaidh, et al. 2012b; Weickenmeier, et al. 2015).

Classical RVE-based multiscale computational homogenisation procedures provide explicit connection between microstructural features and macroscopic response, enabling inverse parameter identification for constituent properties difficult to measure directly (Mazhari and Shafieian 2024). Microstructural parameters derived from histology ensure physical interpretability, facilitating investigation of disease-induced changes or treatment effects at the constituent level. Embedded element techniques (EETs) combine a reinforcement mesh (i.e. fibres) superimposed on a background mesh (i.e. matrix) which permits to account for mechanical interactions between the two phases (Mazhari and Shafieian 2024). Besides the advantage of reduced computational cost compared to FE^2^ methods, through kinematic coupling EET models do not lead to discontinuity in the strain field (Goudarzi and Simone 2019).

The assumption of perfectly periodic microstructure may not capture local heterogeneities and irregular fibre arrangements present in biological tissues. That is the reason why we followed our approach in this study. Periodic boundary conditions, while ensuring kinematic compatibility, impose homogeneous loading on RVE faces, thus assuming affinity of deformation that place constraints on fibre displacements at boundaries and possibly oversimplifying complex kinematics (He, et al. 2024). Additionally, current RVE implementations typically employ fixed volume fractions, neglecting strain-dependent changes in fibre volume during large deformations (Morin, et al., 2021).

A fundamental limitation of our approach is that at the voxel scale considered here (8 × 8 × 50 nm^3^) we are below the characteristic length at which the classical continuum fibre-reinforced homogenisation was originally conceived for (Gasser, et al. 2006; Humphrey and Yin 1987). In our model 1C, each fibre element represents a short segment of a real collagen fibre, and the only deformation energy associated with its anisotropy is an axial stretch energy. The true bending mechanics of a curved fibre segment is therefore not resolved by our formulation, and deviations from the real non-affine kinematics of the fibre network are expected. A direct comparison with an explicit discrete-fibre model, as done recently for the optic nerve head by He et al. (He, et al. 2024), would be needed to quantify the magnitude of this limitation for skin, and this constitutes an important direction for future work.

A major limitation of using a high-resolving power imaging technique such as SBF-SEM or µCT in combination with voxel/subvoxel-level fibre assignment is that, at this scale (smaller than individual collagen fibre diameter (Barton and Marks 1984; Ogura, et al. 2019), the basics of continuum mechanics, namely the assumption of continuity of fundamental physical properties (e.g. density), is no longer satisfied. This limitation constrained the choice of ROI to define RVEs for the FE analyses. It was essential to ensure that individual fibres and fibre bundles were continuously connecting two opposing faces of the RVE so that fibres could effectively be mechanically recruited upon loading of the RVE. It is clear by looking at high-resolution images of the human dermis that geometrical discontinuities of the collagen network are present. The fact that images are acquired every 50 nm along the *z*-axis could potentially contribute to apparent discontinuities in the 3D collagen network. One should keep in mind that collagen networks in the dermis are not simple aggregates of fibres “floating” in a soft matrix but are rather complex multilevel structures interacting with other constituents and structures such as elastin fibres and microvessels. Pure elasticity is only a part of the complex coupled physical phenomena operating in biological structures. The role of interstitial fluid and its interaction with porous solid structures in multiscale tissue mechanics deserves greater attention. These phenomena and other effects such as osmotic pressure gradients and electrochemical effects (Sachs, et al. 2024) remain incompletely integrated into most constitutive models. The physics of solids and liquids at microscopic and sub-microscopic scales is governed by small-range electromagnetic interactions (Israelachvili 2011) which could play a significant role in observed macroscopic tissue mechanics.

These aspects are generally not fully accounted for in most constitutive models of biological soft tissues and including them could offer novel insight into the multiscale mechanical behaviour of soft tissue assemblies.

Experimental validation remains critical for multiscale model credibility. Multimodal approaches combining mechanical testing with real-time microstructural imaging (e.g. multiphoton microscopy, second-harmonic generation) under controlled loading enable simultaneous characterisation of macromechanical response and microstructural evolution (Allain, et al. 2019; Jayyosi, et al. 2017; Niestrawska, et al. 2022, 2023). These datasets provide essential information for model development, parameter identification, and validation of predicted microstructural deformations.

The existence of residual tension lines in skin (Langer 1861; Ní Annaidh, et al. 2012b) complicates the characterisation of skin mechanical properties and the subsequent data interpretation, particularly as these lines are body location-specific and individual/age-dependent. Accurately capturing these aspects in FE models considering the complexity of the multilayer and multiscale nature of skin and that of the structural characteristics of the collagen network is of course a tremendous scientific and technological challenge that will continue to drive the development of advanced research tools from imaging and image processing to experimental characterisation of biophysical properties.

## Conclusion

In this paper, an integrative methodology is presented, combining a high-resolution imaging technique (SBF-SEM), a 3D fibre orientation extraction algorithm, and a software workflow to automatically generate voxel-based FE meshes (in VTK and Abaqus ® formats) that distinguish between fibre and matrix phases, and produce corresponding Abaqus® analysis input files. Importantly, a fibre vector field corresponding to the spatially-varying local orientation of collagen fibres/fibre bundles within each voxel of the imaged volume and extracted from the imaging modalities is defined at finite element centroid or integration point level. This new level of accuracy for characterising and integrating collagen network architecture in FE model of biological soft tissues offers a novel quantitative insight into the micromechanics of these structural materials. The approach is used in combination with invariant-based continuum fibre-reinforced constitutive models which offer a practical and straightforward framework to capture local directions of anisotropy. Departing from classical use of these structure tensor-based models, our study showed how the inclusion of spatially non-uniform fibre orientation distributions led to multifold differences in the stress response of an RVE when compared to spatially uniform fibre orientation distributions with or without fibre dispersion. Additionally, it was shown that the strain distribution in the RVE has a much wider range and lower peak strain values when using spatially-varying fibre orientation distributions. One could argue that this observation is likely to be more in line with natural biological systems which generally tend to favour smooth gradation of structural and material properties.

The general methodology and techniques presented in this paper are generic and should prove particularly useful for researchers interested in biological soft tissue mechanics and its multiple applications.

## Supplementary Information

Below is the link to the electronic supplementary material.Supplementary file1 (PDF 1098 KB)

## Data Availability

The data that support the findings of this study are available from the corresponding author upon reasonable request.
