# Supplementary material for: Integrating serial block-face SEM with voxel-based finite element analysis for high-fidelity micromechanical modelling of anisotropic soft tissues: application to human dermis
Source: Biomech Model Mechanobiol. 2026 Jun 26;25(4):73. doi: 10.1007/s10237-026-02090-6 (PMC13303350; doi:10.1007/s10237-026-02090-6)
Supplement: Supplementary file 1 — Supplementary file1 (PDF 1098 KB) [file 10237_2026_2090_MOESM1_ESM.pdf]

13. Supplementary material 1 – Validation of fibre extraction method

To assess the performance of the 3D structure tensor extraction algorithm for the analysis of biological images that was described in **section 3**, several synthetic image datasets containing fibre-like structures with dimensions of 90×90×90 voxels were created in Mathematica® (Wolfram Research, Inc., Champaign, IL, USA). 3D straight and curved cylindrical splines were generated and embedded into a unit dimensions 3D image stack to mimic fibre structures The tensor extraction algorithm was then applied on the image stacks and histograms of the azimuth and elevation angles θ and ϕ were plotted. For image stacks containing straight fibres, the mean values of the extracted fibre angles were compared to those that were defined as input parameters to the synthetic image generation in Mathematica®. The influence of the parameters controlling the structure tensor extraction algorithm, σ and ρ (see **section 3.1**), were selected according to equation (10).

13.1Structure tensor extraction for a single synthetic fibre

13.1.1 Straight fibre

First, a single straight fibre of 0.02 [unitless] radius was considered (**Figure 32**). The relative percentage errors between the input and measured output angles were respectively 0.01% and 2.96% for the azimuth and elevation angles, suggesting that the structure tensor extraction this algorithm works well for a single straight fibre. The procedure was repeated by generating a single straight fibre with a 0.04 [unitless] radius. In that case the relative errors for azimuth and elevation angles were respectively 0.03% and 6.31%.

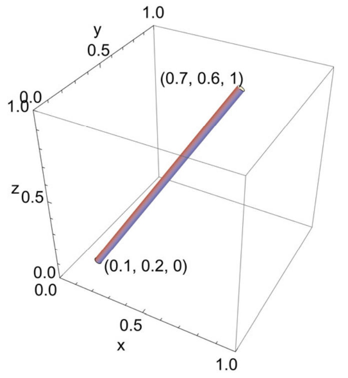

a) Synthetic solid straight fibre of 0.02 radius generated in Mathematica®. $\theta \simeq 56.33^{\circ},\ \phi \simeq 35.80^{\circ}$

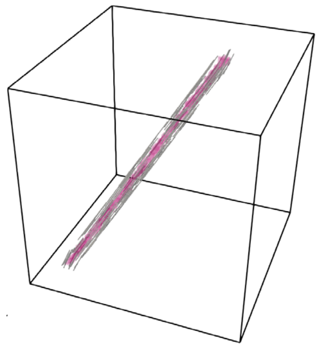

b) Visualisation of the single fibre and its extracted voxel-level fibre vectors in ParaView.

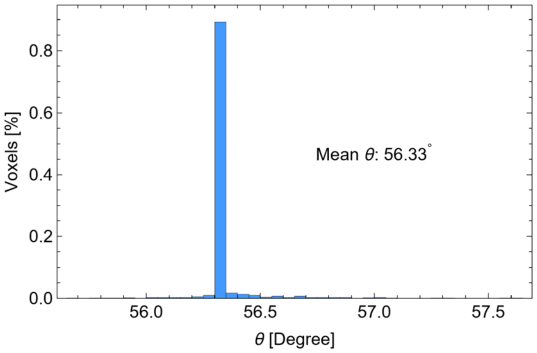

c) Histogram of azimuth angles of extracted fibre vectors. The relative percentage error of mean θ between the input and measured output angle is 0.01 %.

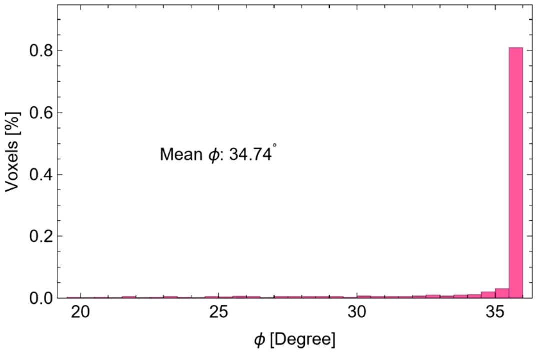

d) Histogram of inclination angles of extracted fibre vectors. The relative percentage error of mean ϕ between the input and measured output angle is is 2.96 %.

Figure 32. Synthetic generation of a single straight solid fibre embedded in a unit cube image stack and statistics of fibre orientation after application of voxel-based structure tensor extraction algorithm. The parameters of the structure tensor analysis are $\sigma = 1,\ \rho = 4$.

13.1.2 Curved fibre

Similarly to the procedure used for a single straight fibre a curved fibre of 0.02 [unitless] radius was generated (**Figure 33**)

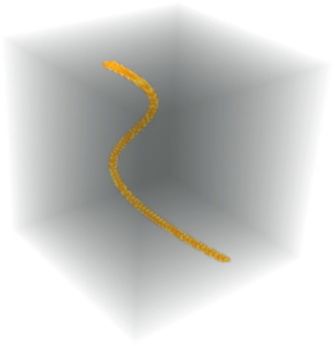

a) Synthetic solid curved fibre of 0.02 radius generated in Mathematica®.

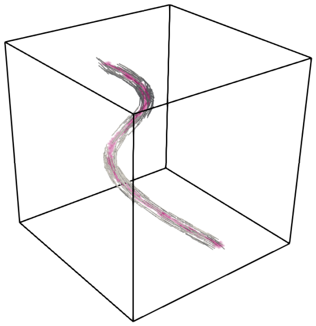

(b) Visualisation of the fibres and their extracted voxel-level fibre vectors in ParaView based on (a). $\sigma = 1, \ \rho = 4$

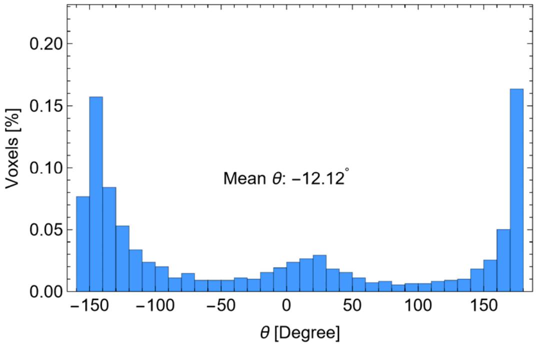

(c) Histogram of azimuth angles of extracted fibre vectors.

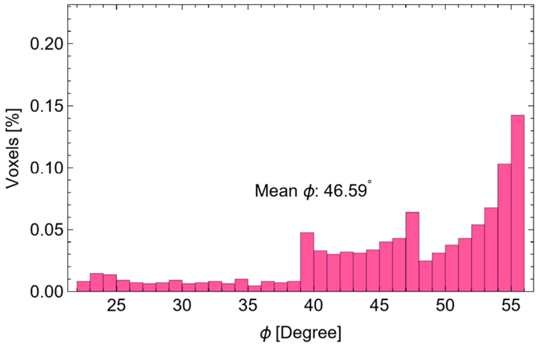

(d) Histogram of inclination angles of extracted fibre vectors.

Figure 33. Synthetic generation of a single curved solid fibre embedded in a unit cube image stack and statistics of fibre orientation after application of voxel-based structure tensor extraction algorithm. The parameters of the structure tensor analysis are $\sigma = 1, \ \rho = 4$.

13.2 Structure tensor extraction for multiple synthetic fibres

In order to evaluate the performance of the structure tensor extraction algorithm in the presence of multiple fibres, two additional types of image stacks embedded in the unit cube were created in Mathematica®. Sets of straight fibres of non-uniform length (**Figure 34**) and combined straight and curved fibres (**Figure 35**) were created. Two normal distribution functions were employed for azimuth (mean = 45°) and inclination (mean = 65°) angles, both having a standard deviation of 10°. These distributions were used to generate lists of azimuth and inclination angles from which solid fibres were generated.

In order to evaluate the influence of the Gaussian kernel parameters used in the structure tensor extraction technique, ρ and σ , a simple parametric sensitivity analysis was devised for the case presented in **Figure 34** (set of straight fibres). The results are collected in **Figure 36**. When σ varies and $\rho = 4\sigma$ an increase in σ will result in a lower measured fibre dispersion (**Figure 36-a-b**). While the calculated mean values of azimuth and elevation angles are similar to those of the input fibres generated in Mathematica®, the extracted angle dispersion is significantly lower, suggesting that the parameters σ and ρ have a significant influence on the ability of the structure tensor extraction algorithm to faithfully capture fibre dispersion. A similar trend could be observed in the bar chart (**Figure 36-c**) when only ρ changes but σ remains constant. This result is to be expected as σ is the size of the Gaussian kernel used to reduce the influence of noise on structure tensor extraction and the synthetic images generated in Mathematica® are noise-free.

Figure 36-d demonstrates that, for the set of straight fibre considered, the recommended setting for the parameter ρ , $\rho = 4\sigma$ (Jeppesen, et al., 2021) is indeed appropriate for maximising the precision of the structure tensor algorithm.

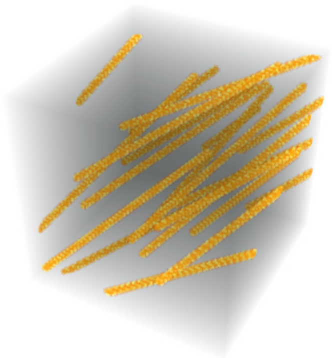

a) Synthetic solid straight fibres of 0.02 radius generated in Mathematica®. $\theta \simeq 42.91^{\circ},\ \phi \simeq 61.69^{\circ}$

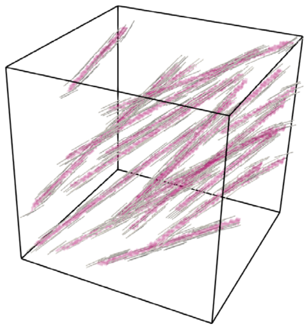

b) Visualisation of the fibres and their extracted voxel-level fibre vectors in ParaView.

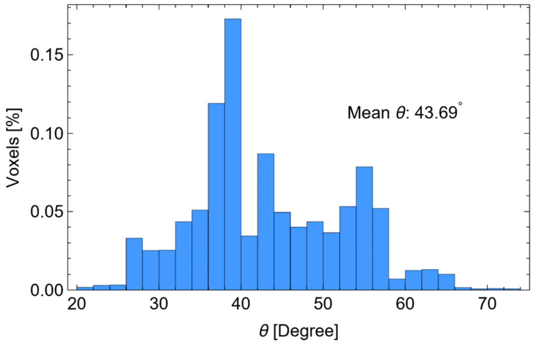

c) Histogram of azimuth angles of extracted fibre vectors. The relative percentage error of mean θ between the input and measured output angles is 1.85%.

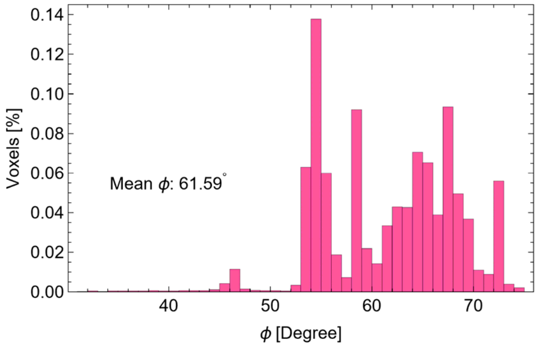

d) Histogram of inclination angles of extracted fibre vectors. The relative percentage error of mean ϕ between the input and measured output angles is is 0.16 %.

Figure 34. Synthetic generation of multiple straight solid fibres embedded in a unit cube image stack and statistics of fibre orientation after application of voxel-based structure tensor extraction algorithm. The parameters of the structure tensor analysis are $\sigma = 1,\ \rho = 4$.

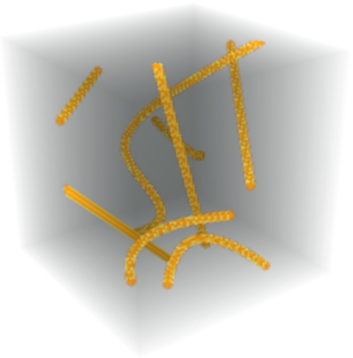

a) Synthetic solid straight and curved fibres of 0.02 radius generated in Mathematica®.

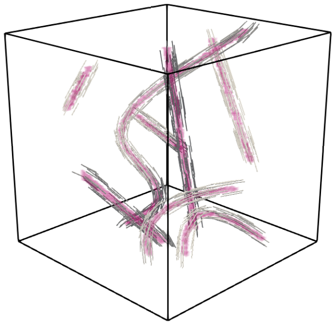

b) Visualisation of the fibres and their extracted voxel-level fibre vectors in ParaView.

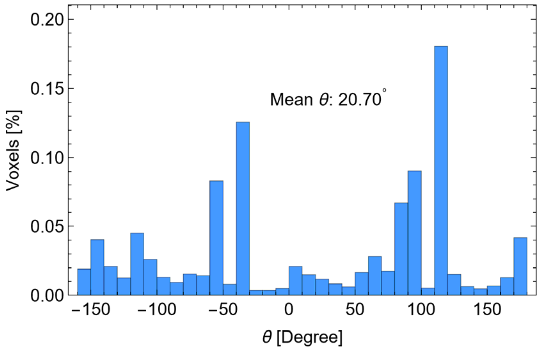

c) Histogram of azimuth angles of extracted fibre vectors. Mean: $\bar{\theta} \simeq 20.70\%$.

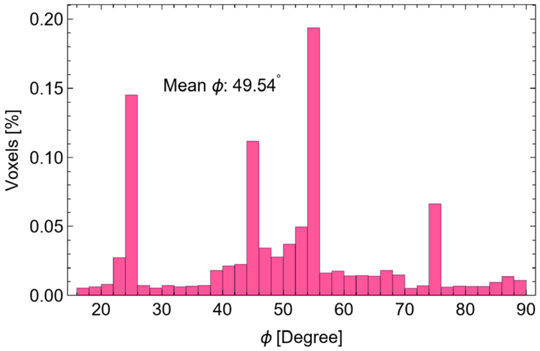

d) Histogram of inclination angles of extracted fibre vectors. Mean: $\bar{\phi} \simeq 49.54\%$.

Figure 35. Synthetic generation of multiple straight and curved solid fibres embedded in a unit cube image stack and statistics of fibre orientation after application of voxel-based structure tensor extraction algorithm. The parameters of the structure tensor analysis are $\sigma = 1,\ \rho = 4$.

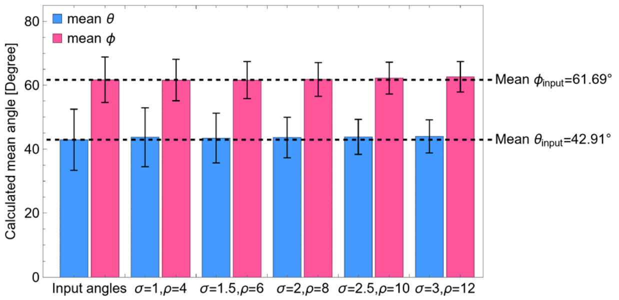

a) Bar chart of the mean values and standard deviation of azimuthal and elevation fibre angles calculated after structure tensor extraction presented for different values of the Gaussian kernels parameters $\sigma,\ \rho=4\sigma$.

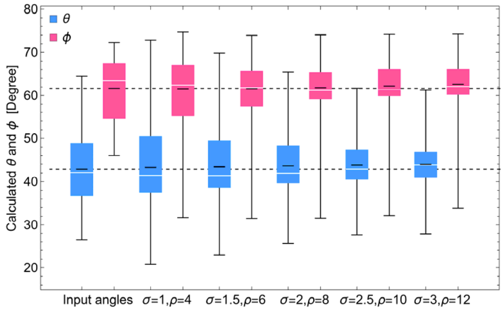

b) Box whisker chart of the distribution of the azimuthal and elevation fibre angles calculated after structure tensor extraction, presented for different values of the Gaussian kernels parameters $\sigma,\ \rho=4\sigma$.

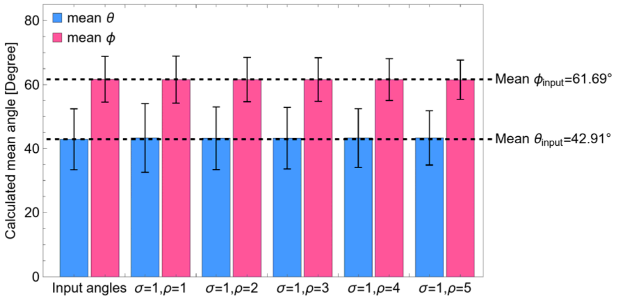

c) Bar chart of the mean values and standard deviation of azimuthal and elevation fibre angles calculated after structure tensor extraction presented for different values of the Gaussian kernel ρ while $\sigma=1$ is kept constant.

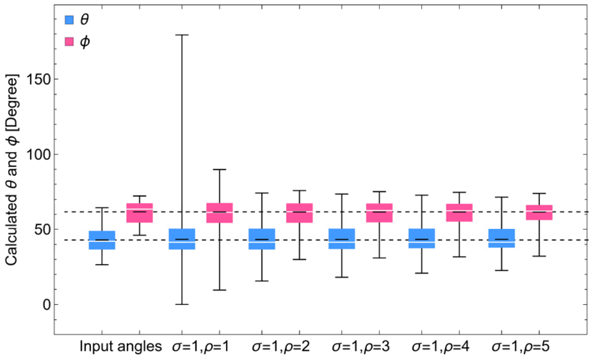

d) Box whisker chart of the distribution of the azimuthal and elevation fibre angles calculated after structure tensor extraction, presented for different values of the Gaussian kernel ρ while $\sigma=1$ is kept constant.

Figure 36. Influence of the Gaussian kernel parameters σ and ρ of the 3D structure tensor extraction algorithm on the directional statistics of the set of generated straight fibres (**Figure 34**). Short black line located in each box represents the mean value for each group of data.

14. Supplementary material 2 – Finite element Lagrange shape functions

14.1 Shape function of 8-noded hexahedral element

The topological characteristics of a 8-noded tri-linear interpolation hexahedron are visually represented in **Figure 37**.

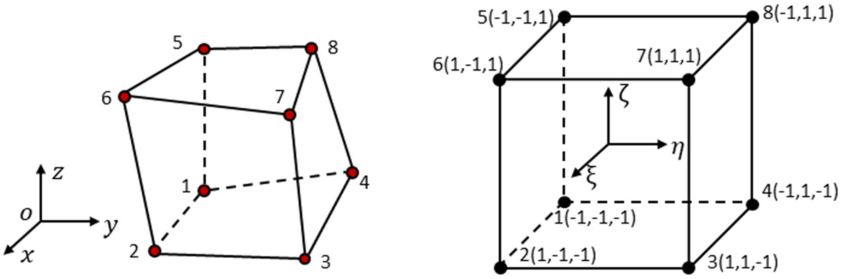

Figure 37. Cartesian global and parametric coordinate systems of 8-noded hexahedral element.

The 8 shape functions of the hexahedral element are given in the parametric coordinate system as (Zienkiewicz, et al., 2025):

$$
\begin{aligned}
N_1 &= \frac{1}{8}(1-\xi)(1-\eta)(1-\zeta); & N_5 &= \frac{1}{8}(1-\xi)(1-\eta)(1+\zeta) \\
N_2 &= \frac{1}{8}(1+\xi)(1-\eta)(1-\zeta); & N_6 &= \frac{1}{8}(1+\xi)(1-\eta)(1+\zeta) \\
N_3 &= \frac{1}{8}(1+\xi)(1+\eta)(1-\zeta); & N_7 &= \frac{1}{8}(1+\xi)(1+\eta)(1+\zeta) \\
N_4 &= \frac{1}{8}(1-\xi)(1+\eta)(1-\zeta); & N_8 &= \frac{1}{8}(1-\xi)(1+\eta)(1+\zeta)
\end{aligned}
\tag{42}
$$

14.2 Shape function of 27-noded hexahedral element

The topological characteristics of a 8-noded tri-linear interpolation hexahedron are visually represented in **Figure 38**.

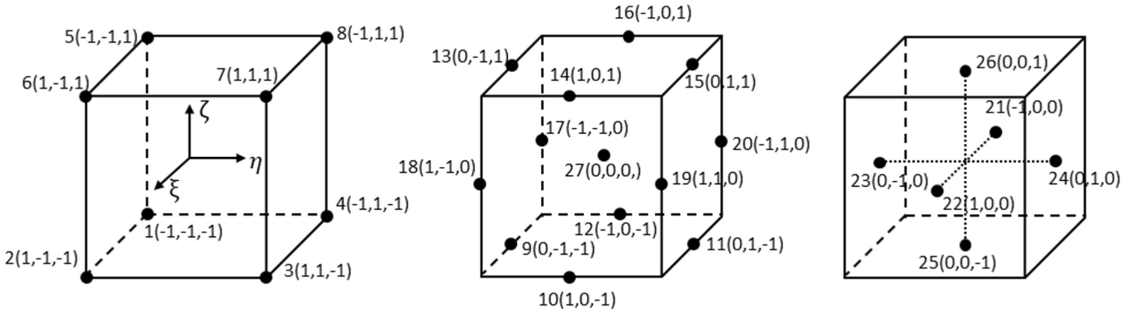

Figure 38. Parametric coordinate system of 27-noded hexahedral element. For sake of visibility the 27 nodes are indicated on 3 different parametric cubes.

The corresponding shape functions are given in the parametric coordinate system as (Zienkiewicz, et al., 2025):

$$
\begin{aligned}
&N_1 = \frac{1}{8}(1-\xi)(1-\eta)(1-\zeta) && N_{10} = \frac{1}{4}\zeta\xi\left(1-\eta^2\right)\left(\zeta-1\right)\left(\xi+1\right) && N_{19} = \frac{1}{4}\eta\xi\left(1-\zeta^2\right)\left(\eta+1\right)\left(\xi+1\right)\\
&N_2 = \frac{1}{8}(1+\xi)(1-\eta)(1-\zeta) && N_{11} = \frac{1}{4}\zeta\eta\left(1-\xi^2\right)\left(\zeta-1\right)\left(\eta+1\right) && N_{20} = \frac{1}{4}\eta\xi\left(1-\zeta^2\right)\left(\eta+1\right)\left(\xi-1\right)\\
&N_3 = \frac{1}{8}(1+\xi)(1+\eta)(1-\zeta) && N_{12} = \frac{1}{4}\zeta\xi\left(1-\eta^2\right)\left(\zeta-1\right)\left(\xi-1\right) && N_{21} = \frac{1}{2}\xi\left(1-\zeta^2\right)\left(1-\eta^2\right)\left(\xi-1\right)\\
&N_4 = \frac{1}{8}(1-\xi)(1+\eta)(1-\zeta) && N_{13} = \frac{1}{4}\zeta\eta\left(1-\xi^2\right)\left(\zeta+1\right)\left(\eta-1\right) && N_{22} = \frac{1}{2}\xi\left(1-\zeta^2\right)\left(1-\eta^2\right)\left(\xi+1\right)\\
&N_5 = \frac{1}{8}(1-\xi)(1-\eta)(1+\zeta) && N_{14} = \frac{1}{4}\zeta\xi\left(1-\eta^2\right)\left(\zeta+1\right)\left(\xi+1\right) && N_{23} = \frac{1}{2}\eta\left(1-\zeta^2\right)\left(1-\xi^2\right)\left(\eta-1\right)\\
&N_6 = \frac{1}{8}(1+\xi)(1-\eta)(1+\zeta) && N_{15} = \frac{1}{4}\zeta\eta\left(1-\xi^2\right)\left(\zeta+1\right)\left(\eta+1\right) && N_{24} = \frac{1}{2}\eta\left(1-\zeta^2\right)\left(1-\xi^2\right)\left(\eta+1\right)\\
&N_7 = \frac{1}{8}(1+\xi)(1+\eta)(1+\zeta) && N_{16} = \frac{1}{4}\zeta\xi\left(1-\eta^2\right)\left(\zeta+1\right)\left(\xi-1\right) && N_{25} = \frac{1}{2}\zeta\left(1-\eta^2\right)\left(1-\xi^2\right)\left(\zeta-1\right)\\
&N_8 = \frac{1}{8}(1-\xi)(1+\eta)(1+\zeta) && N_{17} = \frac{1}{4}\eta\xi\left(1-\zeta^2\right)\left(\eta-1\right)\left(\xi-1\right) && N_{26} = \frac{1}{2}\zeta\left(1-\eta^2\right)\left(1-\xi^2\right)\left(\zeta+1\right)\\
&N_9 = \frac{1}{4}\zeta\eta\left(1-\xi^2\right)\left(\zeta-1\right)\left(\eta-1\right) && N_{18} = \frac{1}{4}\eta\xi\left(1-\zeta^2\right)\left(\eta-1\right)\left(\xi+1\right) && N_{27} = \left(1-\zeta^2\right)\left(1-\eta^2\right)\left(1-\xi^2\right)
\end{aligned}
\tag{43}
$$
